# Supplementary material for: Species Discrimination of Three Odontomachus (Formicidae: Ponerinae) Species in Thailand Using Outline Morphometrics
Source: Insects. 2022 Mar 14;13(3):287. doi: 10.3390/insects13030287 (PMC8955869; doi:10.3390/insects13030287)
Supplement: Supplementary file 1 [file insects-13-00287-s001.zip › insects-1586006-supplementary.pdf]

Table S1. Specimens used in this study.

Species	Localities	Date
Odontomachus monticola	N Thailand, Chiang Mai Prov., Doi Chiang Dao W.S.	26 AUG 2014
	N Thailand, Chiang Mai Prov., Doi Chiang Dao W.S.	29 NOV 2013
	N Thailand, Chiang Mai Prov.,, Mae Sala Luang	8 MAR 2008
	N Thailand, Chiang Mai Prov.,, Doi Suthep-Pui N.P.	20-24 DEC 2004
	W Thailand, Tak Prov., Thung Yai Naresuan East W.S.	19 FEB 2015
	NE Thailand, Loei Prov., Phu Luang W.S.	14 APR 2010
	NE Thailand, Loei Prov., Phu Luang W.S.	11 APR 2008
	NE Thailand, Loei Prov., Phu Luang W.S.	9 APR 2008
	Central Thailand, Uthai Thani Prov., Ban Rai District	23 OCT 2013
Odontomachus rixosus	N Thailand, Prayao Prov., Nammin Forest Park	29 JUL 2017
	W Thailand, Kanchanaburi Prov., Thong Phaphum Dist.	21 DEC 2003
	E Thailand, Chachoengsao Prov., Khao Ang Reu Nai W.S.	27 FEB 2003
	E Thailand, Chachoengsao Prov., Khao Ang Reu Nai W.S.	21 AUG 2003
	E Thailand, Chachoengsao Prov., Khao Ang Reu Nai W.S.	24 APR 2003
	E Thailand, Chachoengsao Prov., Khao Ang Reu Nai W.S.	26 OCT 2002
	E Thailand, Trat Prov., Koh Kud	23 OCT 2016
	E Thailand, Trat Prov., Koh Kud	18 APR 2009
	S Thailand, Nakhon Si Thammarat Prov., Khao Nan N.P.	16 APR 2007
	S Thailand, Nakhon Si Thammarat Prov., Khao Nan N.P.	26 NOV 2006
	S Thailand, Nakhon Si Thammarat Prov., Khao Luang N.P.	20 MAY 2003
	S Thailand, Trang Prov., Khao Chong B.G.	10 MAR 2007
	S Thailand, Phuket Prov., Tha Lang Dist.	7 APR 2012
	S Thailand, Songkhla Prov., Had Yai Dist.	19 OCT 2011
Odontomachus simillimus	NE Thailand, Srisaket Prov., Phanom Dongrak	13 SEP 2018
	Central Thailand, Uthai Thani Prov., Ban Rai	6 FEB 2014
	Central Thailand, Bangkok, Bang Kean	25 JUL 2003
	S Thailand, Narathiwat Prov., Hala-Bala W.S.	6 NOV 2002

Remark: All colonies were collected from rotting logs in primary forest.

Table S2. Coordinates of head and pronotum of three *Odontomachus* species.

**HEAD Monticola Rixosus Simillimus 25 33 22 (80)**
41 2.2069087313708557 1.6041088987910737 1.3774656072195253 3.120381273497292 1.293130978206085
2.955727950185337 1.1766688714732387 2.819186169877862 1.0762705036000955 2.6665806507106846
0.9758721357269523 2.505943262113655 0.935712788577695 2.31317839579722 1.0039836787314325 2.096317921191231
1.0762705036000955 1.87142557715539 1.1485573284687587 1.6385013636896977 1.1365095243239816
1.4216408890837082 1.1204457854642786 1.2369078921971246 1.144541393753833 1.0762705036000955
1.1887166756180159 0.8714578331388833 1.2409238269120504 0.6867248362522997 1.321242521210565
0.5100237087955677 1.481879909807594 0.32529071190898406 1.6987403844135835 0.25300388704032095
1.9196167937344988 0.26906762590002387 2.1003338559061566 0.29717916890450397 2.301130591652443
0.3774978632030186 2.5742141522673925 0.32529071190898406 2.8392658434524907 0.32930664662390985
2.995887297334594 0.39757753677764723 3.1123494040674404 0.5260874476552706 3.2127477719405837
0.7549957264060372 3.236843380230138 0.9919358745866552 3.285034596809247 1.1847007409030903 3.285034596809247
1.4176249543687824 3.232827445515212 1.634485428974772 3.208731837225658 1.8232343605762813 3.2488911843749153
2.020015161607642 3.297082400954024 2.22884376678378 3.3372417481032812 2.393497090095735 3.3171620745286527
2.550118543977838 3.252907119089841 2.6505169118509815 3.148492816501772 2.819186169877862 3.028014775054
2.963759819615188 2.927616407180857 3.0561263180584803 2.8111543004480106 3.180620294221178 1.3774656072195253
3.120381273497292
40 1.9380242013482063 1.2176438611363627 1.4018270454223898 2.3071033060807045 1.3444028050074968
2.1753653427759496 1.293734357582591 2.1044295163810816 1.216042738197736 1.9862031390563017
1.1653742907728302 1.878110451216503 1.0876826713879748 1.7463724879117484 1.1011942573679496
1.621390317596981 1.1383511188128805 1.4727628718172578 1.1856416697427925 1.3444028050074968
1.236310117167698 1.2565774961376603 1.2565774961376603 1.1923974627327798 1.2430659101576855
1.0809268783979873 1.2430659101576855 0.9255436396282767 1.259955392632654 0.7431372288986163
1.3038680470675723 0.5303297497140126 1.3376470120175095 0.3411675459943648 1.4491175963523018
0.21956327217459118 1.6653029720318993 0.1317379633047547 1.8578430722465409 0.11822637732477986
2.0267378969962264 0.1587611352647044 2.182121135765937 0.14862744577972326 2.3611496500006037
0.17565061773967294 2.5131549922753207 0.24658644413454087 2.600980301145157 0.3546791319743396
2.6516487485700626 0.513440267239044 2.675294024035019 0.7296256429186415 2.695561403004981 0.9356773291132578
2.682049817025006 1.1248395328329055 2.661782438055044 1.2498217031476728 2.719206678469937 1.4018270454223898
2.773253022389836 1.5808555596570566 2.800276194349786 1.7767735563666918 2.7800088153798237
1.8612209687415344 2.7023171959949686 1.9625578635913459 2.6178697836201255 2.074028447926138
2.546933957225258 2.1787432392709434 2.4591086483554214 2.263190651645786 2.4084402009305155 2.35777175350561
1.4018270454223898 2.3071033060807045 NA NA
33 1.9937354143352695 1.4092696240831764 1.3043376374217446 2.8932580320991423 1.1818089502700049
2.7667768066521856 1.0434701099373958 2.608675274843489 0.9486091908521779 2.375475515425662
0.8616533483573949 2.197611292140879 0.8735109632430471 2.0316046837417474 0.940704114261743
1.8063100009143553 1.0395175716421783 1.5612526266108762 1.0869480311847872 1.395246018211745
1.0632328014134826 1.154141182203483 1.0711378780039176 0.8458431951765253 1.114615799251309
0.6047383591682634 1.2094767183365267 0.32015560191261005 1.38734094162131 0.12648122544695706
1.5968254712678327 0.06719315101869593 1.7509744647813117 0.08300330419956556 2.0474148369226173
0.16600660839913112 2.335950132473488 0.11462361056130482 2.6719158875669677 0.14229137862782668
2.877447878918273 0.2687726040747837 2.98811895118436 0.47825713372130635 3.0474070256126216
0.8181754271100034 3.063217178793491 1.1027581843656569 3.043454487317404 1.3715307884404404 3.082979870269578
1.6719236988769635 3.1738882510595783 1.9604589944278343 3.2213187106021874 2.1501808325982696
3.169935712764361 2.3201399792926183 3.051359563907839 2.5612448153008804 2.8458275725565336
2.7667768066521856 2.7114412705191415 2.956498644822621 1.3043376374217446 2.8932580320991423 NA NA NA NA
NA NA NA NA NA NA NA NA NA NA NA NA
39 2.06986980973503 1.5564828335892087 1.3550568918178194 3.1794858997773536 1.2611824685894766
3.016226033293279 1.0734336221327907 2.8366401801607966 0.9999666822149571 2.677461810338824 0.914255525231081

2.5590984071378697 0.8367068157308825 2.3999200373158973 0.8489513057171881 2.2652306474665354
0.9305812389592254 2.0489113243751365 0.9877221922286515 1.8611624779184508 1.0162926688633647
1.6244356715165427 0.9713962055802441 1.3917903617767362 0.9632332122560403 1.212204508644254
1.0244556621875682 0.8897662723382067 1.1305745754022167 0.5754910293563631 1.248937978603171
0.34284571961655674 1.4162793417493476 0.19999333644299142 1.64076165816495 0.1550968731598709
1.8325920012837378 0.15917836982197275 2.0203408477404237 0.18774884645668583 2.106052277644563
0.20815632976719517 2.3509420773706746 0.1673413631461765 2.5999133737588886 0.1550968731598709
2.8407216768228984 0.22448231641560262 2.9754110666722604 0.33876422295445485 3.1182634498458257
0.5550835460458537 3.2121378730741683 0.881603279014003 3.2733603230056962 1.1999600186579487
3.2366268530467797 1.4489313150461625 3.2039748797499645 1.6774951281238668 3.2529528396951872
1.975444384457303 3.342745766261428 2.2407416674939244 3.3509087595856317 2.42849051395061 3.2529528396951872
2.6325653470557033 3.163159913128946 2.7713362335671667 3.04071501326589 2.918270113402834 2.950922086699649
3.0203075299553808 2.8407216768228984 3.163159913128946 1.3550568918178194 3.1794858997773536 NA NA NA NA
40 2.333254306938495 1.6647707753701744 1.6707253062028729 3.172732047001022 1.5719632191366932
3.016358742479571 1.4114748276541513 2.9381720902188455 1.3127127405879715 2.806489307463939 1.259216610093791
2.654231089903579 1.1604545230276113 2.514318133226491 1.1481092621443387 2.3867504374326756
1.2221808274439736 2.1892262633003163 1.2880222188214268 1.8888249151406864 1.3209429145101532
1.711876175813781 1.3456334362766982 1.5472726973701483 1.3003674797046991 1.349748523237789
1.2756769579381542 1.1275338273388846 1.3127127405879715 0.9217794792843437 1.3744390450043338
0.6707591746578037 1.4526256972650595 0.46088973964217184 1.6048839148254197 0.2715957394319941
1.8682494803352323 0.22221469589890427 2.102809437117409 0.2551353915876308 2.25918274163886
0.3086315220818115 2.5102030462654 0.23867504374326756 2.8517552640359383 0.20986943501563182
3.123351003467932 0.2757108263930849 3.3085299167170192 0.43208413091453607 3.3908316559388356
0.6542988268134404 3.3867165689777448 0.8806286096734355 3.419637264666471 1.209835566560701 3.374371308094472
1.4937765668759677 3.41140709074429 1.7036460018915995 3.477248482121743 1.9711266543625028 3.571895482226832
2.2427223937944967 3.571895482226832 2.3455995678217674 3.543089873499196 2.4937426984210367 3.477248482121743
2.6624612638257603 3.407292003783199 2.8229496553083027 3.2797243079893836 2.9381720902188455
3.176847133962113 3.0945453947402966 3.1192359165068417 3.176847133962113 1.6707253062028729 3.172732047001022
NA NA
35 2.034124075462336 1.6819459438213666 1.3809805109543645 3.0871337584997276 1.2846330334459204
2.9546559769256167 1.15215525187181 2.7980913259743954 1.0357353832157734 2.6415266750231736
0.9233299927892553 2.4849620240719523 0.8751562540350333 2.3444552860388046 0.9032576016416628
2.1878906350875833 0.9755182097729957 1.9189205937098435 1.0156629920681808 1.6820663781682519
1.0357353832157734 1.505429336069438 0.9835471662320328 1.3127343810525498 0.9795326880025143
1.0357353832157734 1.0196774702976994 0.8069101241332187 1.0798946437404768 0.5379400827554791
1.3809805109543645 0.24488317200062856 1.6539650305616225 0.22481078085303607 2.0072391147592508
0.24488317200062856 2.320368416661694 0.20875286793496206 2.633497718564137 0.2569266066891841
2.8181637171219878 0.42553469232896113 2.9385980640075426 0.7306350377723673 3.010858672138876
1.0758801655109582 3.0148731503683943 1.3287922939706238 3.0028297156798387 1.569660987741734
3.0710758455816536 1.722211160463437 3.1272785407949124 1.882790289644177 3.235669452991912 2.1437313745628797
3.2557418441395045 2.324382894891212 3.167423323090097 2.4889765023014707 3.091148236729246 2.633497718564137
3.042974497975024 2.7499175872201733 2.930569107548506 2.858308499417173 2.778018934826803 3.091148236729246
1.3809805109543645 3.0871337584997276 NA NA NA NA NA NA NA NA NA NA NA NA
40 2.2928594840370415 1.4710170475484776 1.6732536554309578 2.9560814579280255 1.5417837253613826
2.784772155110094 1.4142977325666428 2.633382538666341 1.258924178848054 2.4620732358484094 1.1672936215268348
2.314667556679492 1.1593257469771636 2.187181563884752 1.1832293706261774 1.9959525746926425
1.2509563042983827 1.75293240092767 1.2668920533977253 1.6174785335832593 1.246972367023547 1.3744583598182867
1.207132994275191 1.1155024369539719 1.2230687433745335 0.9680967577850542 1.2111169315500265
0.7210926467452461 1.3186832379705882 0.525879720278301 1.466088917139506 0.36253829201004084
1.621462470858095 0.24700411103980807 1.7808199618515195 0.20716473829145193 1.9879847001429714
0.21114867556628752 2.2668603093814643 0.25098804831464366 2.493944734047094 0.1991968637417807
2.673221911414697 0.2031808010166163 2.8285954651332856 0.28285954651332856 2.856483026057135
0.3226989192616847 2.995920830676382 0.525879720278301 3.1552783216698064 0.8485786395399858

3.1951176944181623 1.0915988133049581 3.2070695062426693 1.3585226107189443 3.2030855689678335
1.4302334816659854 3.2827643144645458 1.5856070353845744 3.370410934510929 1.8087075227753686
3.4301699936334638 1.9999365119674781 3.4739933036566555 2.1314064420370533 3.4421218054579703
2.2668603093814643 3.374394871785765 2.3903623649013683 3.3465073108619157 2.49792867132193 3.310651875388395
2.6254146641166693 3.187149819868491 2.7210291587127244 3.055679889798916 2.920226022454505 1.6732536554309578
2.9560814579280255 NA NA
38 2.4958513585160333 1.4385185197600086 1.6947654492781108 3.020055966486112 1.5863325887792743
2.8393345323213848 1.493963855761747 2.74294976743353 1.393563059003565 2.5903405563610935 1.293162262245383
2.4136351540666934 1.2289057523201468 2.2690580067349115 1.2690660710234194 2.088336572570184
1.3574187721706195 1.8072143416472746 1.4297073458365106 1.6264929074825472 1.4377394095771652
1.4096271864848742 1.4297073458365106 1.0923606687290193 1.5019959195024015 0.7469819278808735
1.5863325887792743 0.5020039837909096 1.71082957675942 0.2891542946635639 1.899583074664802
0.16064127481309107 2.120464827532802 0.11646492423949102 2.3132343573085112 0.09638476488785465
2.5742764288797844 0.16867333855374564 2.7027894487302575 0.12851301985047286 2.8232704048400756
0.11646492423949102 3.032104062097094 0.14457714733178195 3.1445529544662576 0.20080159351636384
3.3453545479826214 0.35742683645912765 3.4578034403517854 0.5702765255864733 3.51001185466604
0.755013991621528 3.5501721733693126 0.9357354257862555 3.5622202689802944 1.2610340072827648
3.5180439184066943 1.5421562382056744 3.5341080458880034 1.7630379910736744 3.59836455581324
1.9999838714229838 3.654589001997822 2.2248816561613114 3.64254090638684 2.4578115046402935 3.558204237109967
2.598372620101748 3.449771376611131 2.7389337355632026 3.2971621655386945 2.91965516972793 3.148568986336585
3.0923445401520033 1.6947654492781108 3.020055966486112 NA NA NA NA NA NA
36 2.2640934553001797 1.7859386093275267 1.4535188486428963 3.395948582738403 1.3345945792084775
3.206550672157662 1.1672196814859621 3.083221800151598 1.0967460403396398 2.8762054792842764
0.9910355786201565 2.7088305815617613 0.9822263734768663 2.4357452221197624 1.0306770017649627
2.290393337255473 1.101150642911285 2.0569494009582803 1.1451966686277364 1.8323146698043782 1.131982860912801
1.5239924897892185 1.10555524548293 1.1892426943441878 1.2244795149173489 0.819256078325996 1.2993577586353162
0.5593845265989328 1.4711372589294767 0.3832004237331272 1.700176592655024 0.2862991671569341
1.9468343366671519 0.2862991671569341 2.2507519141106664 0.33474979544503064 2.5899063121273422
0.26868075687035353 2.832159453567825 0.2995129748718695 3.052389582150082 0.41403264173464316
3.184527659299436 0.5770029368855133 3.2417874927308232 0.8236606808976412 3.294642723590565
1.1231736557695107 3.334284146735371 1.352212989495058 3.3078565313055 1.6297029515087018 3.334284146735371
1.9248113238089262 3.400353185310048 2.197896683250925 3.488445236742951 2.51943267098102 3.479636031599661
2.6956167738468255 3.3563071595935967 2.8453732612827602 3.3122611338771453 3.0083435564336307
3.2505966978741134 3.140481633582985 3.0964356078665336 3.2241690824442424 2.99072514614705 3.4091623904533384
1.4535188486428963 3.395948582738403 NA NA NA NA NA NA NA NA NA NA
37 2.333820563938088 1.5534992484150338 1.5409706651085526 3.0040731317142795 1.4016275730508645
2.770468536205802 1.2581861547561854 2.5737488768302423 1.1598263250684053 2.3770292174546825
1.1229413889354878 2.196702863027086 1.168022977542387 1.9794915724665718 1.237694523571231 1.7827719130910118
1.2786777859411396 1.5450689913455435 1.2540878285191945 1.315562722074057 1.2540878285191945
1.049171516669653 1.2704811334671577 0.852451857294093 1.315562722074057 0.6311422404965881 1.4467091616577636
0.3729476875661657 1.631133842322351 0.25819455293042237 1.9139183526747183 0.18852300690157825
2.1598179268941684 0.2213096167975049 2.3524392600327375 0.25819455293042237 2.6393220966220956
0.21311296432352325 2.8852216708415455 0.27868618411537655 3.0286630891362245 0.43442258112102816
3.1311212450609953 0.6352405667335789 3.2253827485117847 0.9057300983749738 3.2458743796967386
1.2008095874383138 3.208989443563821 1.4262175304728095 3.184399486141876 1.4958890765016535 3.233579400985766
1.6885104096402226 3.311447599488592 1.9508032888076359 3.360627514332482 2.1557196006571773 3.409807429176372
2.3278493026107925 3.3278409044365556 2.4630940684314897 3.266366010881693 2.557355571882279
3.2540710321707205 2.651617075333068 3.168006181193913 2.7827635149167746 3.0614496990321514 2.852435060945619
2.9753848480553438 2.9753848480553438 1.5409706651085526 3.0040731317142795 NA NA NA NA NA NA NA NA
36 2.357065234588299 1.8518851144391524 1.6695873238445955 3.431684763258416 1.4977828235017479
3.2510697757185 1.290736374370624 3.070454788178583 1.171794797210191 2.916271262229874 1.1101213868307072
2.7532772490840953 1.123337117626311 2.5550412871500403 1.1673895536116565 2.3391843508218475
1.1850105280057945 2.1277326580921887 1.1894157716043292 1.889849503771323 1.101310899633638 1.555050990282697

1.1189318740277763 1.3524097847501075 1.1497685792175183 1.0792846816409654 1.268710156377951
0.709244219364063 1.4757566055090752 0.5682764242109573 1.7092345162314064 0.47136106504319714
1.9735491321434795 0.48017155224026625 2.352400081617451 0.5550606934153536 2.6431461591207315
0.45814533424759346 2.8986502878357356 0.4405243598534552 3.167370147346343 0.5814921550065609
3.277501237309707 0.7709176297435467 3.3612008656818637 1.044042732852689 3.4228742760613473
1.3039521051662275 3.436090006856951 1.5594562338812317 3.4008480580686746 1.7885289010050283
3.405253301667209 1.9559281577493413 3.436090006856951 2.0308172989244286 3.506573904433504 2.286321427639433
3.629920725192471 2.471341658777884 3.603489263601264 2.6916038387046117 3.506573904433504 2.8810293134415974
3.4669267120466927 3.0484285701859104 3.3127431860979835 3.167370147346343 3.110101980565394 3.378821840076002
1.6695873238445955 3.431684763258416 NA NA NA NA NA NA NA NA NA NA
34 2.2790567197925395 1.6804188997073883 1.5695503823916357 3.2173608946532144 1.4391168326083974
3.056492849920554 1.252162077919089 2.9086681601662168 1.2130320129841174 2.7695390403974294
1.1173807431430758 2.6173665656503178 1.0739028932153298 2.4521507359248824 1.1086851731575267
2.1956314213511803 1.182597518034695 1.960851031741351 1.2478142929263143 1.7652007070664935 1.260857647904638
1.4912902525216927 1.217379797976892 1.2434665079335396 1.2825965728685111 0.9825994083670628
1.3434655627673557 0.699993383836713 1.486942467528918 0.41738735930636295 1.747809567095395
0.29130159451589915 1.952155461755802 0.29999716450144837 2.2391092712789264 0.4217351442991376
2.5738887157225716 0.32608387445809606 2.93040708513009 0.29130159451589915 3.1434485497760463
0.40869178932081374 3.32605551947258 0.6695588888872906 3.3651855844075516 0.9652082683959644
3.391272294364199 1.1782497330419204 3.417359004320847 1.3130310678179336 3.3651855844075516
1.6738972222182265 3.4347501442919453 1.9782421717124494 3.491271349198015 2.221718131307828 3.547792554104085
2.469541875895981 3.517358059154663 2.560845360744248 3.4086634343352977 2.717365620484134 3.247795389602637
2.9695371500650616 3.034753924956681 3.2390998196170875 1.5695503823916357 3.2173608946532144 NA NA NA NA
NA NA NA NA NA NA NA NA NA NA
36 2.43543113652688 1.7037603505706327 1.6942585295389916 3.305550790878909 1.4890261818886497
3.100318443228567 1.3798600395214466 2.9736857180826113 1.3274602911851892 2.8033865359897745
1.2139275031232981 2.6898537479278835 1.1615277547870406 2.5457544400031753 1.174627691871105
2.3099555724900167 1.253227314375491 2.1090898705343633 1.3143603541011248 1.855824420242452
1.3492935196586298 1.641858781202734 1.3099937084064368 1.3230936454905011 1.2925271256276842
0.9912285726942038 1.384226685216135 0.6375302714244658 1.4890261818886497 0.35806494696442603
1.6898918838443033 0.1921324105662774 1.9693572083043431 0.14409930792470804 2.235722595680319
0.1921324105662774 2.4365882976359723 0.20959899334502988 2.895086095578225 0.13973266223001993
3.1658181286488887 0.1964990562609655 3.3666838306045426 0.3536983012697379 3.454016744498305
0.5894971687828965 3.5151497842239383 0.8383959733801195 3.576282823949572 1.1265945892295355
3.5544495954761315 1.4715595991098973 3.571916178254884 1.785958089127442 3.6636157378433345
2.0610567678927936 3.7553152974317854 2.344888738047522 3.7640485888211614 2.554487731392552
3.6636157378433345 2.711686976401324 3.5981160524230127 2.890719449883537 3.519516429918627 3.0173521750294925
3.357950539215166 3.1527181915648246 3.2182178769851464 3.309917436573597 1.6942585295389916 3.305550790878909
NA NA NA NA NA NA NA NA NA NA
37 2.3098769057229416 1.824416662743451 1.6184054863255692 3.394704190829243 1.499985572691991
3.201723590833782 1.3464782772410564 3.0613740635643563 1.2719175908791738 2.890323077204743
1.1534976772455956 2.7061143226636215 1.1184102954282389 2.5833084863028737 1.1315680636097476
2.3947138090345823 1.188585059062952 2.2543642817651564 1.2412161317889867 2.0745414499512043 1.3113908954237
1.8552453135927258 1.3070049726965303 1.631563254507078 1.2938472045150216 1.3464782772410564
1.3245486636052086 1.078936990883713 1.3815656590584129 0.815781627253539 1.499985572691991 0.5263107272603478
1.6666506363244347 0.3859611999909217 1.9210341545002694 0.3333301272648869 2.1271725226772387
0.33771604999205646 2.3640123499443955 0.37718935453658253 2.6227817908474 0.33771604999205646
2.929796381749269 0.3596456636279043 3.201723590833782 0.5175388818060086 3.293827968104343 0.7192913272558086
3.394704190829243 0.986832613613152 3.412247881737921 1.1841991363357824 3.3990901135564124 1.7192817090504693
3.4385634181009386 2.0087526090436607 3.52628187264433 2.2806798181281738 3.596456636279043 2.4912041090323127
3.587684790824704 2.671026940846265 3.4999663362813127 2.802604622661352 3.4429493408281084 2.9341823044764386
3.377160499920565 3.0569881408371864 3.232425049923969 3.184179899925104 3.12277698174473 3.3508449635575475
1.6184054863255692 3.394704190829243 NA NA NA NA NA NA NA NA

41 2.2046782842924655 1.8142734481583276 1.561163274366512 3.359132213945922 1.438375151663528
3.1924911902775865 1.2454223874159815 3.096014808153813 1.20156948645063 2.938144364678548 1.1796430359679544
2.872365013230521 1.074396073651111 2.749576890527537 1.074396073651111 2.639944638114158 1.0963225241337866
2.490844774831963 1.1621018755818138 2.3242037511636275 1.20156948645063 2.1093245364334052 1.232266517126376
1.9339129325719995 1.2673488378986573 1.7628866188071288 1.2366518072229111 1.5743191446561176
1.2191106468367707 1.3243576091536142 1.2629635478021222 1.0261578825892241 1.319972319057079
0.7454993164109749 1.4032928308912467 0.5218495214876824 1.5962455951387933 0.40783197897776857
1.7848130692898045 0.4122172690743037 1.9602246731512103 0.4560701700396552 2.2014156284606434
0.5437759719703581 2.429450713480471 0.4604554601361904 2.6531005084037633 0.4034466888812335
2.9469149448716183 0.40783197897776857 3.1091706784434185 0.5613171323564987 3.205647060567192
0.7893522173763263 3.245114671436008 1.017387302396154 3.29773815259443 1.232266517126376 3.2889675724013596
1.469072182339274 3.2582705417256137 1.6927219772625663 3.2845822823048243 1.9032159018962533
3.363517504042457 2.188259758171038 3.455608596069695 2.4119095530943304 3.477535046552371 2.5741652866661306
3.4249115653939493 2.740806310334466 3.3372057634632464 2.8899061736166614 3.2801969922082894
3.082858937864208 3.15302357940877 3.1574088695053053 3.0521619071884616 3.293352862497895 1.561163274366512
3.359132213945922
35 2.319016673994786 1.762120336974373 1.5477675713302808 3.243355641046038 1.4434236901170034
3.0303202169022634 1.3129938386004067 2.856413748213468 1.2303882659732288 2.682507279524672
1.1477826933460509 2.5129484725530964 1.1477826933460509 2.36512797416762 1.18691164880103 2.2694794163887826
1.2521265745593284 2.1216589180033063 1.3260368237520666 1.865146876687333 1.3738611026414853
1.612982497088579 1.3564704557726057 1.391251749510365 1.391251749510365 1.1521303550632709 1.4955956307236422
0.8347510497062188 1.612982497088579 0.6217156255624443 1.8347132446667935 0.4434614951564288
2.139049564872186 0.4217231865703293 2.3520849890159603 0.4391138334392089 2.4738195170981174
0.46519980374252823 2.695550264676332 0.46085214202530833 2.9477146442750852 0.49128577404584756
3.1390117598327607 0.591281993541905 3.2694416113493574 0.7651884622307007 3.360742507410975
0.9999621949605747 3.4085667863003937 1.2521265745593284 3.399871462865954 1.5564628947647206
3.365090169128195 1.9955767282039296 3.4259574331692733 2.291217724974882 3.491172358927572 2.5129484725530964
3.4868246972103516 2.6694642943730127 3.4085667863003937 2.8129371310412687 3.317265890238776
2.973800614578405 3.204226685591059 3.112925789529441 2.9824959380128444 3.3346565371076555 1.5477675713302808
3.243355641046038 NA NA NA NA NA NA NA NA NA NA NA NA
37 2.3311330196982385 1.6984265316047762 1.6429193285750614 3.1858726662579895 1.5820704645537629
3.0467895484950214 1.5212216005324644 2.964208947323259 1.3908311776296818 2.8468575667107547
1.329982313608383 2.7121207963778793 1.2734797970171774 2.6078084580556533 1.1691674586949512
2.364413001970459 1.225669975286157 2.2122908419172123 1.2995578815977338 1.990627122982482 1.3169432713181048
1.7993878360584006 1.3430213558986615 1.5864168119838558 1.334328661038476 1.3212896187481975
1.3256359661782904 1.0213916460717976 1.3821384827694962 0.7432254105458611 1.5386069902528354
0.408556658428719 1.742885319467195 0.26947354066575085 1.9732417332621108 0.2347027612250088
2.168827367616285 0.2347027612250088 2.3687593494005514 0.26947354066575085 2.6251938477760244
0.21731737150463776 2.890321041011682 0.25643449837547255 3.1337164970968767 0.3694395315578842
3.268453267429752 0.5519861236217799 3.355380216031607 0.8518840962981801 3.4162290800529056
1.1517820689745801 3.4162290800529056 1.3821384827694962 3.390150995472349 1.6429193285750614
3.4509998594936477 1.9645490384019253 3.568351240106152 2.25575431621814 3.572697587536245 2.468725340292685
3.48577063893439 2.6382328900663023 3.446653512063555 2.7295061860982504 3.3249557840209576 2.9250918204524243
3.1945653611181752 3.055482243355207 3.1206774548065983 3.1641409291075258 1.6429193285750614
3.1858726662579895 NA NA NA NA NA NA NA NA
36 1.9062061855670103 1.3386907216494845 1.4192439862542956 2.5670103092783507 1.3127147766323024
2.457044673539519 1.2199312714776633 2.3539518900343643 1.1134020618556701 2.3024054982817868
1.092783505154639 2.2061855670103094 1.0206185567010309 2.0859106529209623 0.9759450171821306
1.9828178694158076 0.9862542955326461 1.8144329896907216 1.0309278350515463 1.6460481099656357
1.0756013745704467 1.443298969072165 1.0996563573883162 1.2852233676975946 1.0756013745704467
1.0515463917525774 1.0687285223367697 0.7972508591065293 1.1134020618556701 0.5463917525773195
1.1890034364261168 0.3436426116838488 1.388316151202749 0.19243986254295534 1.5910652920962198
0.14432989690721648 1.8041237113402062 0.15807560137457044 1.9518900343642611 0.1958762886597938

2.1718213058419242 0.140893470790378 2.529209621993127 0.15807560137457044 2.6597938144329896
0.2920962199312715 2.745704467353952 0.5017182130584192 2.8247422680412373 0.7285223367697594
2.845360824742268 0.9896907216494846 2.821305841924399 1.1821305841924399 2.8350515463917527
1.3402061855670102 2.8969072164948453 1.5326460481099657 2.9587628865979383 1.7766323024054982
2.9690721649484537 2.0034364261168385 2.8900343642611683 2.1305841924398625 2.8419243986254297
2.2646048109965635 2.70446735395189 2.3848797250859106 2.5876288659793816 2.515463917525773 1.4192439862542956
2.5670103092783507 NA NA NA NA NA NA NA NA NA NA
39 2.4437948955741717 1.7139589501929264 1.736154493919108 3.3446505691676935 1.617006636493287
3.1744393442736634 1.4382848503545551 3.021249241869036 1.3276475541734356 2.8255063332409014
1.2212655386146667 2.655295108346871 1.195733854880562 2.451041638474035 1.246797222348771 2.251043449223549
1.3063711510616818 2.02976885686131 1.35317923790754 1.7829625807649663 1.3148817123063832 1.57870911089213
1.3148817123063832 1.2978605898169802 1.3616897991522416 0.9957356656300766 1.4084978859980999
0.7957374763795911 1.5319010240462718 0.5787181646397027 1.7574308970308619 0.399996378500971
1.910620999435489 0.36595413352216494 2.0893427855742206 0.38297525601156795 2.327638500425863
0.4042516591233217 2.557423654032804 0.3702094141445157 2.7829535270173937 0.3616988528998142
2.9276330681773195 0.37446469476686645 3.0638020480925436 0.4553150265915308 3.2425238342312754
0.625526251485561 3.365926972279447 0.9148853338054123 3.434011462237059 1.12764936492295 3.4467773041041116
1.4170084472428013 3.434011462237059 1.6382830396050407 3.434011462237059 1.7616861776532124
3.4808195490829177 1.961684366903698 3.5531593196628806 2.170193117398885 3.625499090242843 2.391467709761124
3.629754370865194 2.5744447765222067 3.5659251615299326 2.693592633948028 3.510606513439373 2.846782736352655
3.4127350591253056 2.9957175581349316 3.3063530435665367 3.123375976805454 3.1872051861407154
3.2765660792100815 1.736154493919108 3.3446505691676935 NA NA NA NA
39 2.166531498633876 1.636506423963107 1.3546884845286409 3.196552007657487 1.264945714260182
3.0085195366188113 1.1367417567338123 2.8717686485906837 1.0598193822179904 2.713650434308161
0.9615296814477735 2.542711824273001 0.8846073069319517 2.380320144739599 0.9615296814477735
2.1580999516938917 1.0854601737232643 1.893145106139394 1.1239213609811753 1.6751983783445654
1.0854601737232643 1.4743455115532527 1.0555459169671113 1.2093906659987552 1.0555459169671113
1.0042643339565636 1.0854601737232643 0.6837544401406389 1.1581090829882073 0.4615342470949313
1.311953832019851 0.2777752413071346 1.5256270945638006 0.17948554053691773 1.7264799613551134
0.16666514478428074 1.901692036641152 0.18375900578779672 2.162373416944771 0.1923059362895547
2.4572425192554213 0.15384474903164377 2.773478947820467 0.17948554053691773 2.9828787451135375
0.35897108107383546 3.1409969593960603 0.67948097488976 3.2008254729083663 0.9487092856951366
3.235013194915398 1.205117200747876 3.209372403410124 1.3974231370374308 3.153817355148697 1.5982760038287436
3.1751846814030924 1.7991288706200563 3.277747847424188 2.046989855171038 3.358943687190889 2.2563896524641085
3.3888579439470417 2.452969054004542 3.3333028956856148 2.572626081029154 3.273474382173309 2.628181129290581
3.243560125417156 2.816213600329257 3.123903098392544 2.9230502316012315 3.0256133976223274 3.0427072586258435
2.9059563705977154 3.2179193339118823 1.3546884845286409 3.196552007657487 NA NA NA NA
38 2.2716633227117096 1.628327277323524 1.6539937155160862 3.1307738186554492 1.6075806265602777
3.004192666957789 1.4725607314161073 2.8987083738764063 1.4092701555672775 2.7805659656252573
1.350198951441703 2.632887955311321 1.257372773530086 2.476771201550874 1.2531534018068307 2.3037769608974057
1.30800523420915 2.0885890030113843 1.3712958100579797 1.9113753906346609 1.3966120403975117
1.6961874327486395 1.3670764383347245 1.455683244523086 1.3333214645486817 1.2278371714672986
1.388173296951001 0.9367005225626815 1.4345863859068095 0.654002617104575 1.5485094224347031
0.4430340309418088 1.6835293175788735 0.367085339923213 1.8438654430625758 0.33754973786042575
2.021079055439299 0.33333036613717043 2.2067314112625334 0.3628659681999577 2.3037769608974057
0.37130471164646833 2.5611586360159806 0.33754973786042575 2.894489002153151 0.37130471164646833
3.0463863841903427 0.49366649162087267 3.1476513055484703 0.7046350777836388 3.1982837662275343
0.9071649204998943 3.248916226906598 1.1054753914928943 3.2446968551833426 1.350198951441703 3.206722509674045
1.5949225113905119 3.2446968551833426 1.873401045125363 3.3333036613717044 2.1392214636904483
3.4008136089437895 2.3797256519160017 3.350181148264726 2.527403662229938 3.282671200692641 2.6497654422043424
3.248916226906598 2.7847853373485125 3.143431933825215 2.907147117322917 3.0168507821275554 3.080141357976385
1.6539937155160862 3.1307738186554492 NA NA NA NA NA NA
38 1.9837083034681176 1.4465727128509673 1.4322248237597477 2.7948530448304028 1.3479763047150566

2.659322818541117 1.2490758693147672 2.53844450860743 1.1721533084478752 2.4871628013628353 1.1428494757366785
2.3662844914291483 1.0512749985141883 2.2051134115175652 0.996330312180694 2.0842351015838783
1.0329601030696902 1.9413789171167934 1.0512749985141883 1.8314895444498052 1.117208622114381
1.6446776109159251 1.1648273502700761 1.4065839701374503 1.1245345802921805 1.2087830993368713
1.1208716012032807 0.9853413749139952 1.1684903293589757 0.7875405041134163 1.2161090575146707
0.6117175078462349 1.3113465138260605 0.4249055743123548 1.4725175937376433 0.3076902434675673
1.6483405900048247 0.2490825780451735 1.8021857117386082 0.2490825780451735 2.0659202061393804
0.30036428528976805 2.3516325750735496 0.26373449440077196 2.5714113204075266 0.2783864107563704
2.6776377139856153 0.3223421598231657 2.8058419820971015 0.40292769977895715 2.8937534802306923
0.5934026124017369 2.9670130620086845 0.8534741277136092 3.00730583198658 1.1062196848476824 2.981664978364283
1.3186724720038596 2.963350082919785 1.494495468271041 3.025620727431078 1.758229962671813 3.113532225564669
2.0549312688726813 3.0732394555867732 2.1758095788063687 2.9670130620086845 2.3662844914291483
2.8937534802306923 2.5237925922518314 2.754560274852507 2.6849636721634145 2.6776377139856153
2.7948530448304028 1.4322248237597477 2.7948530448304028 NA NA NA NA NA NA
36 2.159268813712979 1.6563664347600522 1.4748847791272701 3.1997500292930607 1.337395520056084
3.0205973583821213 1.2249043080887498 2.891440781678886 1.116579437305391 2.687290063664094 1.0374189548098596
2.5747988516967597 0.9915892017861309 2.478973004465327 1.0082545665220322 2.291487651186437
1.0832487078335882 2.0706715684357437 1.1457438255932184 1.8081920738452975 1.1624091903291198
1.5123818497830483 1.154076507961169 1.2290706492727252 1.1624091903291198 0.9957555429701062
1.2082389433528484 0.7707731190354378 1.2998984494003059 0.5166263068129421 1.5248808733349744
0.32497461235007646 1.7123662266138646 0.2541468122224957 1.9665130388363603 0.26664583577442175
2.224826192242831 0.27081217695839704 2.478973004465327 0.24998047103852036 2.6456266518243408
0.24998047103852036 2.8372783462872064 0.28747754169429846 3.0122646760141705 0.4791292361571641
3.166419299821258 0.7957711661392899 3.2497461235007647 1.0832487078335882 3.2455797823167893
1.3123974729522319 3.2080827116610116 1.6123740381984564 3.2664114882366664 1.9206832858126315
3.3580709942841236 2.183162780403078 3.4163997708597784 2.4123115455217214 3.3580709942841236
2.5747988516967597 3.2580788058687156 2.7664505461596254 3.116423205613554 2.9497695582545402
3.0247636995660967 3.0705934525898253 2.9372705347026145 3.203916370477036 1.4748847791272701
3.1997500292930607 NA NA NA NA NA NA NA NA NA NA
43 2.30047652217684 1.587538131548079 1.5141722029234126 3.0650763460794685 1.4529523025356734
2.9344738919189584 1.3713257686853548 2.840603377991092 1.2733739280649723 2.7508141907557415
1.2529672946023926 2.6161304099027154 1.15501545398201 2.477365302357174 1.085632900209239 2.3508441748891795
1.1142021870568508 2.175347127110994 1.1795034141371057 1.987606099255261 1.2529672946023926
1.7794584379369485 1.3019432149125838 1.5794734300036675 1.3141871949901316 1.420301688995546
1.3060245416050997 1.2448046412173608 1.3223498483751635 0.9795184062038248 1.363163115300323
0.7672894181929961 1.41622036230303 0.5509791034896514 1.5223348563084445 0.387726035789014 1.7019132307791456
0.2693675617060518 1.869247625172299 0.23671694816592434 2.0937205932606755 0.22447296808837652
2.277380294423893 0.2693675617060518 2.4569586688945937 0.33058746209379086 2.705919597138066
0.2856928684761156 2.958961852074054 0.2775302150910837 3.1344588998522394 0.3714007290189502
3.2364920671651376 0.5305724700270718 3.301793294245393 0.8040213584256395 3.346687887863068
1.0162503464364683 3.3875011547882274 1.2121540276772331 3.3793385014031956 1.4039763822254823
3.3140372743229407 1.5835547566961834 3.293630640860361 1.673343943931534 3.3221999277079726 1.861084971787267
3.3834198280957115 2.0814766131831277 3.4323957484059027 2.2447296808837653 3.4528023818684823
2.3630881549667273 3.424233095020871 2.530422549359881 3.342606561170552 2.6732689835979384 3.2773053340902973
2.8120340911434805 3.089564306234564 2.9712058321516017 2.9385552186114743 3.154865533314819
1.5141722029234126 3.0650763460794685
41 2.373276859504132 1.608285123966942 1.7231404958677685 3.0289256198347108 1.615702479338843
2.9132231404958677 1.524793388429752 2.8388429752066116 1.43801652892562 2.760330578512397 1.3677685950413223
2.603305785123967 1.2520661157024793 2.3925619834710745 1.2479338842975207 2.2231404958677685
1.3016528925619835 2.0702479338842976 1.359504132231405 1.859504132231405 1.4090909090909092
1.6446280991735538 1.4049586776859504 1.4504132231404958 1.3760330578512396 1.2355371900826446
1.4132231404958677 0.9958677685950413 1.43801652892562 0.8264462809917356 1.5082644628099173
0.5619834710743802 1.6239669421487604 0.4214876033057851 1.8305785123966942 0.30578512396694213

1.975206611570248 0.30165289256198347 2.190082644628099 0.34710743801652894 2.384297520661157
0.40082644628099173 2.5702479338842976 0.3347107438016529 2.8801652892561984 0.30991735537190085
3.0991735537190084 0.4214876033057851 3.1735537190082646 0.5867768595041323 3.2231404958677685
0.743801652892562 3.293388429752066 0.9380165289256198 3.322314049586777 1.1239669421487604 3.3181818181818183
1.322314049586777 3.28099173553719 1.487603305785124 3.3057851239669422 1.6735537190082646 3.347107438016529
1.884297520661157 3.4214876033057853 2.12396694214876 3.458677685950413 2.268595041322314 3.446280991735537
2.396694214876033 3.355371900826446 2.553719008264463 3.3181818181818183 2.677685950413223 3.206611570247934
2.7975206611570247 3.1115702479338845 2.9008264462809916 3.041322314049587 2.9958677685950414
1.7231404958677685 3.0289256198347108
36 1.720221535803051 1.3054074100914579 1.190304292927946 2.6332022294132758 1.0380560694139063
2.3875289596519846 0.9480912100647011 2.2698826051184087 0.8858078458998667 2.1418556898906935
0.8166041079389397 2.0172889615610248 0.8062235472448006 1.8650407380469851 0.8546661638174495
1.7024119538388063 0.9100291541861912 1.4809599923638397 0.9446310231666548 1.2871895260732438
0.9204097148803303 1.103799620476787 0.9134893410842376 0.8996485934920522 0.9584717707588402
0.7197188747936417 0.9896134528412573 0.5259484085030459 1.0311356956178137 0.3702399980909599
1.1384014894572505 0.2352927090671521 1.2802691522771512 0.14186766281990054 1.4671192447716543
0.09342504624725156 1.7335536359212236 0.10380560694139063 1.961925971192283 0.07958429865506615
2.176457558871157 0.1141861676355297 2.3460067168754284 0.22491214837301304 2.491334566593375
0.44290392294993336 2.563998491452349 0.6781966320170855 2.595140173534766 0.9619319576568865
2.5536179307582096 1.1972246667240387 2.563998491452349 1.3806145723204954 2.615901294923044
1.5986063468974157 2.692025406680064 1.8373592428626142 2.712786528068342 1.9446250367020512
2.6470429770054613 2.0934130733180445 2.570918865248441 2.2525816706281767 2.4671132583070507
2.422130828632448 2.373688212059799 2.5536179307582096 2.3287057823851964 2.6228216687191366 1.190304292927946
2.6332022294132758 NA NA NA NA
42 1.8597166519318544 1.4330143320152555 1.3081884928422216 2.6608964893675555 1.2396969487143568
2.5376117099373987 1.1506579413481322 2.407477776094455 1.092440128839447 2.3013158826962643
1.0410714707435482 2.1814556804725007 0.9554570405837168 2.06502005545513 0.9588816177901102
1.9622827392633324 0.9999765442668291 1.7842047245308834 1.065043511188301 1.599277555385648
1.1027138604586266 1.448596158304345 1.1129875920778063 1.287641029603862 1.092440128839447 1.1438087869353457
1.092440128839447 1.0034011214732224 1.109563014871413 0.8492951471855261 1.1677808273800985
0.6575188236275041 1.215724908269604 0.4862899633078416 1.3047639156358284 0.35615602946489805
1.438322426685165 0.26711702209867355 1.5958529781792548 0.21917294120916803 1.7259869120221982
0.21574836400277478 1.873243731897108 0.2362958272411343 2.1129641363446354 0.22602209562195452
2.34925996358577 0.2910890625434263 2.5136396694926457 0.4280721507991563 2.575282059207724 0.5856027022932458
2.616376985684443 0.7568315626129083 2.6643210665739487 0.9314850001389641 2.6745947981931284
1.1677808273800985 2.626650717303623 1.3492834193189407 2.6300752945100165 1.5068139708130301
2.667745643780342 1.708864025990232 2.7191143018762407 1.8903666179290743 2.74993549673378 1.9931039341208718
2.732812610701814 2.1163887135510286 2.671170220986735 2.20200314371086 2.61295240847805 2.2910421510770846
2.554734595969365 2.4143269305072415 2.4828184746351067 2.503365937873466 2.424600662126421 2.599254099652477
2.366382849617736 2.6882931070187017 1.3081884928422216 2.6608964893675555
40 1.8257748210822227 1.2953612892401112 1.3448742637535887 2.6130976627336566 1.2508027478951769
2.4632800634035936 1.1462788413858307 2.3517878964602907 1.0731121068292884 2.2124226877811624
0.9999453722727459 2.118351171922751 0.9651040701029638 2.0068590049794484 0.9720723305369202
1.8640096660833416 1.034786674442528 1.6270888113288235 1.0661438463953319 1.4737870817817822
1.0591755859613754 1.3309377428856757 1.06962797661231 1.202024924857482 1.0487231953104408 0.9651040701029638
1.0765962370462665 0.8118023405559226 1.1288581903009396 0.616691048405143 1.1567312320367653
0.41461149582040685 1.2368662270272641 0.28569867779221314 1.3831996961403488 0.19511129215077969
1.5155966443855209 0.16723825041495402 1.6375412019797582 0.18465890149984507 1.836136624347516
0.20904781301869252 1.9824700934606008 0.18117477128286685 2.170613125177424 0.1637541201979758
2.3273989849414436 0.21601607345264895 2.456311802969637 0.33796063104688623 2.5259944073092013
0.5191354023297531 2.5782563605638744 0.7037943038295982 2.6235500533845912 0.9093579866313125
2.6235500533845912 1.1149216694330268 2.6130976627336566 1.2926123104989153 2.640970704469482
1.4981759933006298 2.693232657724155 1.686319025017453 2.721105699459981 1.801295322177734 2.7420104807618504

1.9406605308568623 2.700200918158112 2.069573348885056 2.602645272082722 2.205454427347206 2.5294785375261797
2.3831450684130946 2.456311802969637 2.491153105139419 2.3901133288470513 2.5956770116487653
1.3448742637535887 2.6130976627336566
41 1.8571944444444444 1.3279930555555555 1.2951388888888888 2.642361111111111 1.2222222222222223
2.5069444444444446 1.1215277777777777 2.375 1.0486111111111112 2.2534722222222223 1.0 2.1493055555555554
0.9236111111111112 2.0381944444444446 0.9131944444444444 1.8958333333333333 0.9548611111111112
1.7326388888888888 1.0069444444444444 1.5729166666666667 1.0347222222222223 1.3680555555555556 1.03125
1.1666666666666667 1.0138888888888888 0.9930555555555556 1.0416666666666667 0.7847222222222222
1.1076388888888888 0.5798611111111112 1.1597222222222223 0.40625 1.2326388888888888 0.28125 1.3263888888888888
0.1909722222222222 1.4791666666666667 0.11805555555555555 1.6215277777777777 0.125 1.9097222222222223
0.1527777777777778 2.142361111111111 0.13194444444444445 2.3541666666666665 0.1840277777777778
2.4895833333333335 0.2916666666666667 2.5520833333333335 0.4027777777777778 2.5833333333333335
0.5520833333333334 2.6284722222222223 0.7083333333333334 2.6909722222222223 0.8611111111111112
2.6944444444444446 0.9722222222222222 2.670138888888889 1.1527777777777777 2.6597222222222223
1.3993055555555556 2.6944444444444446 1.5868055555555556 2.7465277777777777 1.7743055555555556 2.78125
1.9444444444444444 2.7465277777777777 2.079861111111111 2.6666666666666665 2.2152777777777777
2.579861111111111 2.4027777777777777 2.5 2.5 2.4270833333333335 2.6145833333333335 2.3784722222222223
2.6840277777777777 1.2951388888888888 2.642361111111111
37 1.8772110726643598 1.4523979238754325 1.4152249134948096 2.612456747404844 1.3460207612456747
2.4844290657439445 1.2629757785467128 2.3529411764705883 1.1833910034602075 2.2422145328719725
1.1038062283737025 2.141868512110727 1.0311418685121108 2.0207612456747404 1.055363321799308
1.8823529411764706 1.0830449826989619 1.7647058823529411 1.1280276816608996 1.5916955017301038
1.1487889273356402 1.439446366782007 1.1453287197231834 1.301038062283737 1.1211072664359862
1.0865051903114187 1.1384083044982698 0.903114186851211 1.193771626297578 0.6920415224913494 1.273356401384083
0.4844290657439446 1.356401384083045 0.36678200692041524 1.560553633217993 0.25259515570934254
1.6955017301038062 0.2422145328719723 1.8996539792387543 0.2422145328719723 2.1557093425605536
0.23875432525951557 2.3771626297577853 0.3391003460207612 2.519031141868512 0.5224913494809689
2.612456747404844 0.7820069204152249 2.667820069204152 1.0311418685121108 2.643598615916955 1.245674740484429
2.6539792387543253 1.3944636678200693 2.7024221453287196 1.5916955017301038 2.7474048442906573
1.7750865051903115 2.792387543252595 1.9169550173010381 2.7750865051903113 2.0346020761245676
2.7335640138408306 2.110726643598616 2.671280276816609 2.2422145328719725 2.57439446366782 2.3806228373702423
2.4982698961937717 2.508650519031142 2.43598615916955 2.615916955017301 1.4152249134948096 2.612456747404844
NA NA
38 1.8934259042591524 1.5350050490001226 1.3878212659699134 2.640418921409425 1.3024168803717648
2.5478975036780973 1.249039139372922 2.4447005377470012 1.2134539787070269 2.3486206039490845
1.1707517859079526 2.23118957375163 1.1031399806427518 2.127992607820534 1.0675548199768565 2.039029706155796
1.063996303910267 1.882454999225857 1.0960229485095727 1.7579069368952236 1.1351666252420574
1.5977737138986952 1.1636347537747735 1.4056138463028611 1.1565177216415945 1.1885443662409003
1.152959205575005 1.0355281753775507 1.2098954626404372 0.7793150185831053 1.2703902357724592
0.5480114742547864 1.398496814169682 0.40211231552461596 1.5657470692993896 0.3024738656601093
1.7294388083625074 0.28823980139375127 1.8788964831592674 0.30603238172669883 2.1173170596207656
0.2775642531939827 2.3699717003486214 0.3665271548587207 2.5265464072785604 0.5942721831204502
2.6083922768101195 0.8469268238483061 2.6439774374760145 1.1209325609756993 2.6297433732096565
1.2846243000388173 2.6297433732096565 1.4340819748355773 2.6795625981419096 1.6048907460318742
2.729381823074163 1.7579069368952236 2.7614084676734687 1.8504283546265512 2.7507329194737 1.9429497723578788
2.7080307266746257 2.120875575687355 2.647535953542604 2.2276310576850404 2.587041180410582 2.3592961521488527
2.519429375145381 2.4482590538135907 2.490961246612665 2.537221955478329 2.4482590538135907 2.611950792876709
1.3878212659699134 2.640418921409425
41 1.7450714285714288 1.282921768707483 1.2993197278911566 2.496598639455782 1.2142857142857142
2.360544217687075 1.119047619047619 2.2346938775510203 1.0748299319727892 2.13265306122449 1.0034013605442176
2.0170068027210886 0.9591836734693877 1.9149659863945578 0.9625850340136054 1.816326530612245
0.9727891156462585 1.7040816326530612 1.0068027210884354 1.6054421768707483 1.0204081632653061
1.510204081632653 1.0680272108843538 1.3639455782312926 1.0816326530612246 1.2380952380952381

1.0816326530612246 1.1360544217687074 1.0680272108843538 0.9931972789115646 1.0714285714285714
0.8197278911564626 1.1224489795918366 0.673469387755102 1.1666666666666667 0.5272108843537415
1.227891156462585 0.36054421768707484 1.3129251700680271 0.24149659863945577 1.4761904761904763
0.1598639455782313 1.6428571428571428 0.15306122448979592 1.8401360544217686 0.1836734693877551
2.0170068027210886 0.1564625850340136 2.1904761904761907 0.15306122448979592 2.3469387755102042
0.23809523809523808 2.4489795918367347 0.36054421768707484 2.506802721088435 0.54421768707483
2.574829931972789 0.7551020408163265 2.5884353741496597 0.9863945578231292 2.574829931972789
1.1394557823129252 2.574829931972789 1.3503401360544218 2.622448979591837 1.5476190476190477
2.6802721088435373 1.7755102040816326 2.6904761904761907 1.9217687074829932 2.639455782312925
2.0306122448979593 2.568027210884354 2.1666666666666665 2.5 2.295918367346939 2.4183673469387754
2.4183673469387754 2.32312925170068 2.5510204081632653 1.2993197278911566 2.496598639455782
44 1.8503523489932885 1.3396442953020133 1.342281879194631 2.51006711409396 1.278523489932886
2.365771812080537 1.2046979865771812 2.2818791946308723 1.1711409395973154 2.1711409395973154
1.1140939597315436 2.0704697986577183 1.0536912751677852 1.9697986577181208 1.0503355704697988
1.8624161073825503 1.080536912751678 1.7315436241610738 1.1140939597315436 1.5939597315436242
1.157718120805369 1.4664429530201342 1.1711409395973154 1.2986577181208054 1.1711409395973154
1.151006711409396 1.1644295302013423 0.959731543624161 1.1946308724832215 0.8120805369127517
1.2281879194630871 0.6677852348993288 1.238255033557047 0.5805369127516778 1.2852348993288591
0.48322147651006714 1.3389261744966443 0.40939597315436244 1.4328859060402686 0.3422818791946309
1.5570469798657718 0.2953020134228188 1.6778523489932886 0.28523489932885904 1.8724832214765101
0.2953020134228188 2.063758389261745 0.2751677852348993 2.2449664429530203 0.32550335570469796
2.37248322147651 0.40604026845637586 2.4328859060402683 0.5134228187919463 2.4731543624161074
0.6610738255033557 2.51006711409396 0.8221476510067114 2.5503355704697985 0.9765100671140939
2.5503355704697985 1.1275167785234899 2.540268456375839 1.2751677852348993 2.5503355704697985
1.4060402684563758 2.587248322147651 1.5469798657718121 2.6308724832214767 1.6979865771812082
2.6711409395973154 1.8389261744966443 2.684563758389262 1.9328859060402686 2.640939597315436
2.0503355704697985 2.5805369127516777 2.164429530201342 2.530201342281879 2.2751677852348995
2.4496644295302015 2.3691275167785233 2.402684563758389 2.4563758389261743 2.348993288590604 2.540268456375839
1.342281879194631 2.51006711409396
47 1.798342372881356 1.240528813559322 1.240677966101695 2.5762711864406778 1.176271186440678
2.450847457627119 1.0915254237288134 2.3220338983050848 1.047457627118644 2.1932203389830507
0.9898305084745763 2.064406779661017 0.9254237288135593 1.9627118644067796 0.9084745762711864
1.847457627118644 0.9288135593220339 1.7322033898305085 0.9423728813559322 1.6372881355932203 1.0
1.488135593220339 1.0372881355932204 1.3864406779661016 1.0745762711864406 1.2813559322033898
1.0610169491525423 1.152542372881356 1.0508474576271187 1.006779661016949 1.0677966101694916
0.8338983050847457 1.0983050847457627 0.6711864406779661 1.1288135593220339 0.5423728813559322
1.1796610169491526 0.3864406779661017 1.2338983050847459 0.27796610169491526 1.3423728813559321
0.19322033898305085 1.4542372881355932 0.14576271186440679 1.5796610169491525 0.13220338983050847
1.7050847457627119 0.15932203389830507 1.8711864406779661 0.2 2.016949152542373 0.16610169491525423
2.1932203389830507 0.15593220338983052 2.335593220338983 0.1864406779661017 2.461016949152542
0.30847457627118646 2.522033898305085 0.43728813559322033 2.545762711864407 0.5525423728813559
2.5796610169491525 0.6677966101694915 2.610169491525424 0.7593220338983051 2.623728813559322
0.9796610169491525 2.610169491525424 1.0745762711864406 2.5830508474576273 1.2576271186440677
2.606779661016949 1.4338983050847458 2.6338983050847458 1.5694915254237287 2.6847457627118643
1.7423728813559323 2.711864406779661 1.911864406779661 2.694915254237288 2.0203389830508476 2.613559322033898
2.142372881355932 2.5627118644067797 2.288135593220339 2.48135593220339 2.4033898305084747 2.389830508474576
2.5152542372881355 2.328813559322034 2.606779661016949 1.240677966101695 2.5762711864406778
44 1.7910945084836225 1.389552881043895 1.2905331758497676 2.520255887916038 1.2466145075616866
2.4189051149435437 1.1824256846791066 2.3344461374664647 1.1148585026974434 2.2466088008903022
1.0945883481029444 2.1486363870168907 1.0371562434185306 2.0607990504407283 0.9763457796350337
1.949313200170984 0.9797241387341168 1.8209355544058239 1.003372652427699 1.7432332951269112
1.0439129616166969 1.5844504174700027 1.0810749117066116 1.4695862081011752 1.1081017844992769
1.3310734850387655 1.0945883481029444 1.172290607381857 1.0776965526075284 1.0135077297249484

1.0709398344093621 0.8445897747707903 1.0979667072020274 0.6587800243212165 1.131750298192859
0.5304023785560563 1.1858040437781896 0.39864637369181305 1.317560048642433 0.314187396214734
1.4797212853984247 0.2432418551339876 1.6216123675599174 0.21621498234132233 1.7803952452168261
0.22972841873765498 1.9932318684590653 0.21283662324223918 2.2060684917013043 0.24999857333215394
2.3580946511600467 0.35134934630464876 2.432418551339876 0.5033755057633911 2.4864722969252067
0.6689151016184659 2.5236342470151216 0.8513464929689567 2.516877528816955 1.0067510115267821
2.5033640924206226 1.1824256846791066 2.510120810618789 1.3344518441378488 2.5472827607087036
1.4830996444975078 2.5912014289967846 1.631747444857167 2.6317417381857826 1.7635034497214102
2.6486335336811986 1.8817460181893209 2.621606660888533 1.9864751502608988 2.560796197105036
2.0945826414315603 2.5270126061142046 2.1790416189086392 2.456067065033458 2.304040905574716
2.3986349603490447 2.3986349603490447 2.3614730102591297 2.48985065602429 2.3344461374664647 2.537147683411454
1.2905331758497676 2.520255887916038
44 1.9167686844460394 1.3448863908957756 1.403317267860458 2.57274832441084 1.321965542187388
2.4507207359012346 1.2440034717506958 2.31174487120974 1.1863793327322714 2.176158661754623 1.115196572768335
2.0507414180086405 1.0745207099318 1.9592207266264365 1.0507931232771546 1.8812586561897444 1.071131054695422
1.813465551462186 1.0982482965864453 1.694827618188959 1.1355345041866025 1.5626310639702201
1.1694310565503816 1.4134862335695917 1.1863793327322714 1.2643414031689633 1.1762103670231374
1.1219758832410909 1.15587243560487 0.949103466185817 1.1592620908412479 0.7558931177122757 1.2067172641505388
0.616917253020781 1.2507827822234516 0.43048621501999557 1.3117965764782542 0.2847310398557451
1.447382785933371 0.18304138276440757 1.5761896849157317 0.15592414087338421 1.7422827914982497
0.1728724170552738 1.9354931399717912 0.19660000370991923 2.1219241779725766 0.16270345134614006
2.3185241816824957 0.16270345134614006 2.4913965987377695 0.24066552178283218 2.606644876774619
0.42370690454723975 2.633762118665642 0.5626827692387344 2.6846069472113108 0.783010359603299
2.7015552233932008 0.9694413976040845 2.6913862576840666 1.1829896774958932 2.698165568156823
1.3253551974237658 2.7185034995750903 1.4609414068788826 2.765958672884381 1.6270345134614006 2.79985522524816
1.7422827914982497 2.810024190957294 1.8643103800078549 2.7862966043026485 1.9863379685174598
2.7049448786295787 2.104975901790687 2.677827636738555 2.2100552141184027 2.6608793605566654 2.264289697900449
2.5896966005927293 2.3761483207009206 2.535462116810683 2.4608897016103684 2.4880069435013916
2.5558000482289502 1.403317267860458 2.57274832441084
43 1.826290253577871 1.294323694758939 1.3243167668405993 2.5506611198077866 1.2736413803543518
2.4628237832316247 1.2195876347690213 2.3614730102591297 1.1418853754901086 2.2297170053948867
1.0810749117066116 2.0844475641343108 1.0067510115267821 2.006745304855398 0.9797241387341168
1.9155296091801526 0.9966159342295327 1.7837736043159091 1.0303995252203642 1.6486392403525827
1.0641831162111959 1.50674815819109 1.0912099890038611 1.3952623079213455 1.1148585026974434
1.2263443529671876 1.0844532708056949 1.0709398344093621 1.0776965526075284 0.858103211167123
1.0979667072020274 0.7162121290056302 1.1655338891836906 0.5304023785560563 1.2263443529671876
0.33783590990831613 1.320938407741516 0.209458264143156 1.4391809762094268 0.14526944126057595
1.5979638538663352 0.11148585026974432 1.7297198587305787 0.1216209275669938 1.8412057090003229
0.14189108216149277 1.9729617138645663 0.11824256846791065 2.1520147461159738 0.12499928666607697
2.3378244965655477 0.18918810954865703 2.472958860528874 0.3040523189174845 2.540526042510537
0.4527001192771436 2.601336506294034 0.6655367425193828 2.635120097284866 0.8310763383744577 2.635120097284866
1.0439129616166969 2.6114715835912836 1.2128309165708548 2.635120097284866 1.4121541034167615
2.6959305610683626 1.6249907266590007 2.743227588455527 1.7668818088204934 2.763497743050026
1.9189079682792356 2.7094439974646956 2.0135020230535643 2.635120097284866 2.1385013097196413
2.560796197105036 2.283770750980217 2.496607374222456 2.4053916785472107 2.4391752695380426 2.5236342470151216
2.3344461374664647 2.621606660888533 1.3243167668405993 2.5506611198077866
48 1.8015988826886384 1.3242999402571758 1.1952590949779505 2.6161023008390636 1.1211867003595988
2.4847921467428944 1.0336465976288192 2.360215846702939 0.9865096192353225 2.2794095980283733
0.9158041516450776 2.148099443932204 0.8349979029705119 2.0403577790327834 0.8114294137737637
1.942716895217683 0.831630975942405 1.8248744492339415 0.8585663921672603 1.6699958059410238 0.90233644353265
1.4915486734513579 0.9461064948980398 1.3400369571865474 0.9393726408418259 1.1649567517249884
0.9225380057012914 0.9865096192353225 0.9124372246169707 0.8215301948580843 0.9393726408418259
0.7070546759024496 0.9797757651791088 0.555542959637639 1.003344254375857 0.40739817040093523

1.0605820138536743	0.2727210892766591	1.148122116584454	0.15824557032102443	1.2996338328492645	
0.08754010273077947	1.4679801842546096	0.030302343252962126	1.6228588275475273	0.04377005136538974	
1.797939033009086	0.07407239461835187	1.9999546546955003	0.037036197309175935	2.1750348601570595	
0.07407239461835187	2.3736835548153667	0.18181405951777277	2.4881590737710013	0.3400596298387972	
2.525195271080177	0.49493827313171473	2.575699176501781	0.6733854056213806	2.6295700089514913	
0.8484656110829395	2.6228361548952774	0.9999773273477501	2.592533811642315	1.1716906057812022	
2.606001519754743	1.323202322046013	2.632936935979598	1.4646132572265027	2.659872352204453	1.5790887761821375
2.7002754765417363	1.7238666383907344	2.730577819794698	1.8248744492339415	2.740678600879019	
1.9225153330490417	2.7272108927665912	2.020156216864142	2.66323927923256	2.114430173651135	2.6093684467828497
2.2120710574662352	2.579066103529888	2.313078868309442	2.51172756296775	2.393885116984008	2.451122876461825
2.4814252197147875	2.4006189710402217	2.56559839541746	2.3467481385905113	2.6497715711201324	
1.1952590949779505	2.6161023008390636				
47	1.864893525422625	1.3241531627839689	1.2943991050491228	2.5922225463549893	1.2087906986305301
2.441551751058266	1.1437283097523996	2.3285486545857235	1.0957876021579875	2.215545558113181	
1.0375738857933445	2.1162398066676134	0.9793601694287014	2.003236710195071	0.9622384881449828	
1.9313256488034531	0.9793601694287014	1.8525659148983478	1.0238765407663697	1.719016800885343	
1.0649685758472942	1.5751946781021071	1.1060606109282187	1.4074022015216652	1.11633361969845	1.256731406224942
1.1094849471849624	1.11633361969845	1.112909283441706	0.9759358331719576	1.1334553009821684	0.8218407016184907
1.1676986635496054	0.6780185788352548	1.2087906986305301	0.5239234472817877	1.2738530875086604	
0.3664039794715771	1.3731588389542282	0.2807955730529843	1.5238296342509514	0.2225818566883412	
1.6813491020611622	0.22943052920182863	1.8080495435606794	0.2397035379720598	1.9039309587495035	
0.2534008829990346	2.0648747628164577	0.23285486545857237	2.2189698943699248	0.23627920171531608	
2.3422459996126985	0.29106858182321543	2.44497608731501	0.37667698824180823	2.513462812449884	
0.49995309348458183	2.53743316624709	0.5958345086734057	2.5751008650712706	0.7567783127403601	
2.6230415726656826	0.9040247717803397	2.6230415726656826	1.0512712308203194	2.6127685638954516	
1.1882446810900678	2.5887982100982456	1.3320668038733037	2.6059198913819643	1.4964349441970017	
2.6367389176926577	1.6197110494397753	2.6744066165168383	1.7498358271960364	2.7052256428275316	
1.862838923668579	2.6983769703140443	2.0306314002490207	2.6812552890303256	2.1128154704108697	
2.6161929001521953	2.2121212218564374	2.5751008650712706	2.321699982072236	2.486068122395934	2.421005733517804
2.431278742288035	2.5237358212201153	2.355943344639673	2.6230415726656826	1.2943991050491228	
2.5922225463549893					
46	1.666915206290393	1.342452932188943	1.177467544967815	2.5631250036835627	1.1160344556651465
2.4539106227010405	1.0443625181453664	2.334457393501407	0.9692776312198825	2.2423077595474044	
0.941974035974252	2.1467451761876974	0.8771279972658795	2.0784861880736214	0.8225208067746186	
1.9624459082796917	0.8395855538031376	1.866883324919985	0.8498244020202491	1.7576689439374629	
0.8907797948886949	1.6006732712750877	0.9351481371628444	1.4470905480184162	0.9487999347856596	
1.3139855211959675	0.9351481371628444	1.1808804943735187	0.9146704407286215	1.0546013663624778	
0.9146704407286215	0.9010186431058063	0.9556258335970672	0.7542618186605423	0.9999941758712168	
0.6245697412437975	1.0204718723054396	0.5051165120441641	1.0819049616081082	0.37201148522171545	
1.2081840896191494	0.2730359524563049	1.3447020658473017	0.22184171137074776	1.467568244452639	
0.19112516671941346	1.6552804617663486	0.22525466077645157	1.812276134428724	0.18429926790800583	
1.9999883517424335	0.2047769643422287	2.1467451761876974	0.2696230030506011	2.2593725065759234	
0.37542443462741926	2.3242185452842956	0.5187683096669794	2.378825735775557	0.6450474376780204	
2.412955229832595	0.7849783633118766	2.4504976732953367	1.0034071252769206	2.4334329262668177	
1.1262733038822579	2.430019976861114	1.3071596223845598	2.464149470918152	1.4402646492070086	2.494866015569486
1.5836085242465685	2.532408459032228	1.7337782980975363	2.5870156495234893	1.8737092237313926	
2.5631250036835627	1.9795106553082107	2.5016919143808938	2.071660289262214	2.4573235721067443	
2.1774617208390317	2.3890645839926683	2.286676101821554	2.3071537982557766	2.3890645839926683	
2.262785455981627	2.4880401167580786	2.2252430125188853	2.5528861554664513	1.177467544967815	
2.5631250036835627					
49	1.8702140660901891	1.2930279169749952	1.3276723964288837	2.4898080309620543	1.2769978774812165
2.412107101908964	1.192540345901771	2.3073797627504518	1.1452441282172814	2.1992741223287617	1.094569609269614
2.0877901806438937	1.0134903789533463	1.9796845402222032	0.9999771739006351	1.8850921048532243	

1.020246981479702 1.7769864644315339 1.0540299941114801 1.6722591252730215 1.0844347054800807
1.5607751835881534 1.1080828143223254 1.4391563381137518 1.13173092316457 1.2938893837971055
1.1283526219013922 1.1486224294804592 1.1114611155855032 0.9932205713742794 1.1283526219013922
0.8479536170576332 1.1587573332699928 0.7398479766359428 1.192540345901771 0.6249857336878969
1.2161884547440156 0.4932319844239619 1.2736195762180387 0.35472163263367124 1.3378073002184172
0.26012919726469225 1.469561049482352 0.19594147326431363 1.624962907588532 0.18580656947478016
1.7533383555892892 0.18918487073795798 1.8749572010636908 0.21283297958020272 2.023602456643515
0.18580656947478016 2.189139218539228 0.18918487073795798 2.327649570329519 0.24323769094880313
2.4289986082248536 0.32431692126507083 2.5066995372779433 0.4628272730553615 2.5472391524360773
0.6114725286351856 2.5844004663310334 0.7432262778991207 2.6215617802259894 0.8986281360053004
2.6215617802259894 1.0303818852692355 2.6012919726469224 1.1621356345331706 2.5979136713837443
1.2533497686389716 2.6181834789628113 1.418886530534685 2.662101395384123 1.567531786114509 2.678992901700012
1.6688808240098436 2.716154215594968 1.7803647656947117 2.7330457219108575 1.9121185149586468
2.6958844080159015 2.003332649064448 2.6350749852787003 2.0945467831702493 2.6046702739101 2.1823826160128723
2.5371042486465436 2.287109955171385 2.4627816208566315 2.3918372943298976 2.4087288006457865
2.476294825909343 2.378324089277186 2.533725947383366 1.3276723964288837 2.4898080309620543
47 1.7776925579375598 1.1651588913236925 1.2939115349488508 2.4189051149435437 1.2432361484626033
2.3141759828719657 1.1858040437781896 2.2364737235930527 1.1081017844992769 2.1418796688187243
1.0776965526075284 2.0405288958462293 1.003372652427699 1.929043045576485 0.9594539841396178
1.7905303225140756 0.9932375751304494 1.6418825221544164 1.0303995252203642 1.5101265172901732
1.0641831162111959 1.402019026119512 1.0945883481029444 1.2635063030571023 1.0945883481029444
1.1249935799946926 1.0844532708056949 1.003372652427699 1.0709398344093621 0.8648599293652893
1.0878316299047779 0.7162121290056302 1.131750298192859 0.5776994059432206 1.1655338891836906
0.4729702738716426 1.2026958392736053 0.3412142690073993 1.280398098552518 0.2263500596385718
1.3783705124259298 0.1655395958550749 1.4695862081011752 0.1317560048642433 1.5945854947672522
0.1385127230624096 1.6993146268388302 0.15202615945874226 1.7939086816131586 0.16216123675599176
1.8749892999911546 0.14189108216149277 1.9594482774682336 0.1317560048642433 2.1080960778278928
0.1317560048642433 2.263500596385718 0.18918810954865703 2.388499883051795 0.3074306780165677
2.4594454241325416 0.4898620693670584 2.4999857333215396 0.648644947023967 2.533769324312371
0.8040494655817924 2.520255887916038 0.9695890614368673 2.516877528816955 1.0878316299047779
2.5270126061142046 1.2229659938681043 2.557417838005953 1.3918839488222625 2.61822830178945 1.5945854947672522
2.6520118927802816 1.7094497041360797 2.638498456383949 1.8074221180094914 2.6080932244922006
1.9121512500810693 2.5506611198077866 1.9864751502608988 2.496607374222456 2.1317445915214748
2.429040192240793 2.2330953644939697 2.364851369358213 2.344581214763714 2.3209327010701317 2.429040192240793
1.2939115349488508 2.4189051149435437
38 1.6828764308381758 1.32065028238275 1.184557088905037 2.530187096414725 1.1040206296027115
2.4093824074612367 0.9765045690406963 2.224819688226741 0.9093908529554251 2.087236570251935
0.8456328226744174 1.9697875671027103 0.8120759646317818 1.8523385639534857 0.8422771368701538
1.7617350472383695 0.8926124239341072 1.6308633008720907 0.9261692819767429 1.4932801828972846
0.9429477109980606 1.291939034641471 0.9362363393895335 1.1140876870155023 0.9395920251937971
0.9093908529554251 0.9798602548449599 0.7516736201550377 1.0134171128875955 0.5570438435077512
1.100664943798448 0.359058381056201 1.244959433381781 0.23825369210271283 1.429522152616277 0.191274090843023
1.57381664219961 0.19462977664728653 1.6845542737403076 0.2147638914728679 1.8858954219961213
0.18456271923449585 2.0905922560561985 0.23154232049418572 2.26173223207364 0.36241406686046457
2.345624377180229 0.5603995293120146 2.3825369210271283 0.711405390503875 2.432872208091082 0.8657669374999988
2.439583579699609 1.0805308289728666 2.4295165222868182 1.2986504062499982 2.4630733803294538
1.4798574396802304 2.5268314106104617 1.6677758447189899 2.563743954457361 1.8187817059108502 2.55367689704457
1.9697875671027103 2.489918866763562 2.087236570251935 2.4429392655038726 2.208041259205423 2.3825369210271283
2.30871183333333 2.30871183333333 2.3993153500484463 2.244953803052322 2.5268314106104617 1.184557088905037
2.530187096414725
44 1.778772082474612 1.276557230813143 1.0982041341075683 2.5997610211743982 1.0405823122562454
2.454011707079875 0.9321177064184608 2.3184309497826443 0.8880539602968609 2.1964082682151367
0.8338216573779687 2.1015017381070753 0.7490836840671994 1.94558386721526 0.7660312787293533

1.7930555152558756 0.8372111763103993 1.5930738982424604 0.9083910738914455 1.4269874705533527
0.9490653010806147 1.264290561796676 0.9558443389454762 1.10498317197243 0.9456757821481839 0.8982225170941531
0.9863500093373531 0.7287465704726148 1.0405823122562454 0.5457125481213534 1.1015936530399992
0.362678525770092 1.1829421074183375 0.24065584420258443 1.335470459377722 0.12880171943236912
1.4269874705533527 0.10507508690535376 1.5591787089181526 0.1220226815675076 1.6676433147559373
0.1525283519593845 1.7998345531207371 0.1728654655539691 1.9659209808098448 0.16608642768910756
2.138786446363814 0.1525283519593845 2.338768063377229 0.2338768063377229 2.470959301742029 0.3694575636349536
2.5082440099987675 0.5050383209321843 2.5319706425257826 0.6711247486212918 2.559086793985229
0.8304321384455379 2.5828134265122444 0.9829604904049224 2.5760343886473827 1.0880355773102761
2.5489182371879364 1.19311066421563 2.542139199323075 1.450714103080368 2.5692553507825213 1.596463417174891
2.6065400590392596 1.7625498448639987 2.643824767295998 1.9218572346882448 2.640435248363567 2.043879916255752
2.5794239075798133 2.179460673552983 2.4946859342690444 2.25403009006646 2.467569782809598 2.335378544444798
2.4133374798907057 2.4336745934852906 2.3082623929853523 2.5218020857284906 2.2167453818097216
2.6675513998230134 1.0982041341075683 2.5997610211743982
43 1.718439988991761 1.3002040887773723 1.0973092579941846 2.583878069282942 1.0301955419089133
2.4462949513081362 0.9295249677810065 2.3120675191375937 0.8859010523255801 2.201329887596896
0.8322100794573631 2.0973036276647257 0.7583849917635648 1.9832103103197647 0.7281838195251927
1.872472678779067 0.7617406775678284 1.751667989825579 0.8154316504360454 1.597306442829455 0.8691226233042623
1.4060323519864322 0.9194579103682158 1.2181139469476727 0.8993237955426344 1.0402625993217038
0.8758339949127895 0.8389214510658902 0.9228135961724793 0.6375803028100766 0.9731488832364327
0.4228164113372087 1.0503296567344946 0.2516764353197671 1.2114025753391455 0.11073763154069752
1.3590527507267423 0.06711371608527122 1.5603938989825559 0.0771807734980619 1.6979770169573618
0.11073763154069752 1.8825397361918577 0.0771807734980619 2.057035398013563 0.07382508769379835
2.2147526308139502 0.15100586119186024 2.2952890901162757 0.191274090843023 2.3959596642441827
0.31543446560077476 2.4462949513081362 0.47650738420542565 2.469784751937981 0.6308689312015495
2.5033416099806165 0.825498707848836 2.4932745525678257 1.023484170300386 2.476496123546508 1.2181139469476727
2.4966302383720893 1.4127437235949591 2.5771666976744148 1.6644201589147263 2.6174349273255775
1.812070334302323 2.630857670542632 1.9630761954941833 2.5771666976744148 2.06374676962209 2.5100529815891437
2.140927543120152 2.4496506371123994 2.2583765462693766 2.3825369210271283 2.369114177810074 2.318778890746121
2.45971769452519 2.26173223207364 2.5503212112403064 2.2214640024224774 2.6040121841085235 1.0973092579941846
2.583878069282942
47 1.7640272563749153 1.341060705652492 1.1423466283596204 2.610109506934444 1.0948900918699034
2.5016374235293766 1.0169257819225108 2.3796063296986754 0.9559102350071602 2.321980535389733 0.93557171936871
2.220287957197482 0.8982844406982179 2.1355441420372725 0.7999816151123752 2.027072058632205
0.7830328520803334 1.854194675705378 0.8474381516020923 1.6813172927785511 0.9016741933046263
1.5186091676709494 0.9626897402199769 1.3389522795313058 0.9592999876135685 1.1491261335724372
0.9491307297943434 0.9864180084648355 0.9660794928263853 0.8203201307508254 1.0338745449545526
0.62371447957914 1.0711618236250446 0.46100635447153826 1.1084491022955367 0.3288060028216118
1.1694646492108873 0.24067243505499422 1.2779367326159552 0.155928619894785 1.4575936207555988
0.11864134122429293 1.5931837250119336 0.14914911468196826 1.7389430870874936 0.19660565117168544
1.918599975227137 0.15253886728837662 2.091477358153964 0.1423696094691515 2.220287957197482
0.16948763032041847 2.3423190510281833 0.24406218766140259 2.4270628661883924 0.34236501324724533
2.4609603922524763 0.49490388053562195 2.481298907890926 0.6982890369201241 2.5118066813486015
0.881335677666176 2.5185861865614183 1.064382318412228 2.5016374235293766 1.2101416804877878
2.5016374235293766 1.3321727743184892 2.5185861865614183 1.5186091676709494 2.566042723051136
1.7152148188426348 2.606719754328036 1.8202971496412943 2.6270582699664864 1.9457179960784041
2.603330001721628 2.05757983208988 2.552483712625502 2.1524929050693147 2.481298907890926 2.2168982045910735
2.467739897465293 2.284693256719241 2.433842371401209 2.3829960823050835 2.3457088036345914 2.484688660497335
2.311811277570508 2.559263217838319 2.227067462410299 2.6575660434241617 1.1423466283596204 2.610109506934444
42 1.7900606870240483 1.2924613578188886 1.235466098575225 2.5357771028767737 1.1672083030738314
2.4095001811991956 1.0682344995968105 2.25592014132106 0.9999767040954168 2.1057529912179938
0.9078286801685355 1.976063179765346 0.901002900618396 1.8224831398872103 0.9624349165696504
1.6245355329331688 1.0068024836455562 1.4777812726051724 1.0375184916211835 1.2832465554262005

1.0341056018461137	1.1637954132987616	1.0136282631956957	1.030692712071044	1.0272798222959743	
0.8566353335424902	1.064821609821741	0.7235326323147726	1.085298948472159	0.5972557106371944	
1.1194278462228557	0.4470885605341284	1.1842727519491798	0.31398585930641076	1.3446785713774547	
0.20818627627925063	1.5221488396810783	0.19794760695404157	1.7337480057353984	0.19794760695404157	
1.9043924944888826	0.18770893762883253	2.0852756525675757	0.19794760695404157	2.242268582220781	
0.290095630880923	2.3310037163725927	0.4061338832332922	2.3651326141232896	0.5563010333363582	
2.4095001811991956	0.7371841914150513	2.4538677482751017	0.8702868926427689	2.4538677482751017	
0.9931509245452775	2.4333904096246832	1.085298948472159	2.4299775198496136	1.2149887599248068	
2.4504548585000316	1.3685687998029425	2.4982353153510073	1.546039068106566	2.546015772201983	
1.6723159897841442	2.5801446699526798	1.795180021686653	2.5835575597277494	1.911218274039022	2.552841551752122
1.9999534081908337	2.481170866475659	2.1194045503182726	2.4538677482751017	2.2115525742451543	
2.3583068345731504	2.341242385697802	2.3139392674972443	2.4368032993997533	2.2593330310961295	
2.5289513233266345	1.235466098575225	2.5357771028767737			
44	1.7545251980727794	1.3140310726908222	1.1835023287801578	2.6322724209075923	1.1120840848020448
2.499638539233954	1.0134588907370317	2.326194232429965	0.9386397779980562	2.1799568757128767	
0.8706224027808057	2.0847325504087264	0.8230102401287304	1.9963099626263006	0.8264111088895929	
1.8466717371483496	0.8740232715416683	1.7140378554747113	0.9148336966720185	1.5780031050402104	
0.9522432530415063	1.438567485844847	0.9658467280849563	1.2787266540843083	0.9522432530415063	
1.0950797409977322	0.9454415155197812	1.0270623657804818	0.9522432530415063	0.8298119776504554	
0.9964545469327191	0.690376358455092	1.0304632345413443	0.5509407392597286	1.067872790910832	
0.39790164502091513	1.1562953786932575	0.2550651570646892	1.2923301291277585	0.1360347504345009	
1.4691753046926097	0.0918234565432881	1.6154126614096982	0.0918234565432881	1.8126630495397245	
0.12243127539105081	2.033719518995788	0.10882780034760071	2.193560350756327	0.16324170052140108	
2.3091898886256526	0.2550651570646892	2.411215951451528	0.4013025137817776	2.475832457907916	
0.5883502956292164	2.5472507018860293	0.7413893898680298	2.5880611270163794	0.8876267465851183	
2.5676559144512043	1.0984806097585946	2.5506515706468917	1.2719249165625834	2.574457651972929	
1.4317657483231219	2.6118672083424173	1.5814039738010728	2.6526776334727673	1.7344430680398863	
2.68328545232053	1.850072605909212	2.6968889273639802	1.979305618821988	2.6900871898422554	2.043922125278376
2.5914619957772422	2.2173664320823647	2.5370480956034416	2.3534011825168655	2.479233326668779	
2.435022032777566	2.4044142139298033	2.5370480956034416	2.326194232429965	2.6458758959510424	
1.1835023287801578	2.6322724209075923				
39	1.7485545800889497	1.1875303023077768	1.2449384053784351	2.483165552506852	1.154336419003185
2.359007274881509	1.0301781413778426	2.204648335131083	0.9630655588776573	2.060356282755685	0.8959529763774722
1.9361980051303425	0.8791748307524259	1.7583496615048517	0.9227980093775462	1.6039907217544258	
0.9630655588776573	1.463054298504037	1.0066887375027778	1.3019841005035926	1.023466883127824	
1.1342026442531297	1.0066887375027778	0.9798437045027036	1.0133999957527962	0.7986397317522037	
1.0536675452529074	0.6140801298766944	1.1040019821280462	0.3422741707509444	1.1845370811282685	
0.23153840962563887	1.281850325753537	0.1644258271254537	1.3825191995038146	0.12751390675035185	
1.5167443645041851	0.15435893975042592	1.671103304254611	0.20133774750055555	1.8086840983799906	
0.2214715222506111	2.0234443623805833	0.16107019800044445	2.187870189506037	0.12751390675035185	
2.318739725381398	0.2046933766255648	2.409341711756648	0.35569668725098147	2.4428980030067406	
0.5469675473765092	2.5066549563819165	0.7281715201270093	2.536855618507	0.9060198637525	2.510010585506926
1.0972907238780276	2.5167218437569443	1.3187622461286388	2.5603450223820645	1.54023376837925	2.644235750507296
1.7516384032548333	2.6475913796323054	1.8825079391301944	2.6207463466322314	1.9865324420054813	
2.5435668767570183	2.097268203130787	2.4965880690068887	2.2214264807561297	2.3556516457564998	
2.385852307881583	2.2684052885062593	2.486521181631861	1.2449384053784351	2.483165552506852	
39	1.7949027496235854	1.2848504397301646	1.2941098998693366	2.5986003604328123	1.2179857881123168
2.470573445205097	1.1280209287631116	2.3667678382637063	1.0657375645982772	2.2525816706281767	
1.0345958825158599	2.1556964374828786	0.9446310231666548	2.0380500829493027	0.927330088676423	
1.8927222332313558	0.9550115838607939	1.7439341966153628	1.0311356956178137	1.5467035434267205	
1.0657375645982772	1.3806145723204954	1.07265793839437	1.2145256012142704	1.0484366301080454	
1.0207551349236745	1.0518968170060918	0.8235244817350323	1.1107199942728798	0.6159132678522511	
1.1453218632533433	0.439443736051887	1.259508030888873	0.27681495184370836	1.3633136378302637	

0.22145196147496668 1.512101674446257 0.20069084008668855 1.650509150368111 0.20415102698473492
1.8235184952704289 0.24913345665933753 1.9584657842942366 0.21107140078082762 2.1522362505848323
0.19377046629059586 2.3217854085891036 0.22491214837301304 2.4359715762246337 0.2802751387417547
2.529396622471885 0.42560298845970157 2.567458678350395 0.5986123333620194 2.61936148182109 0.8062235472448006
2.6401226032093685 0.9965338266373501 2.6089809211269515 1.2249061619084094 2.6228216687191366
1.474039618567747 2.69548559357811 1.7058721407368527 2.7577689577429445 1.9204037284157267 2.7266272756605274
2.0553510174395346 2.6574235376996 2.1937584933613885 2.5743790521464875 2.3702280251617527 2.4671132583070507
2.519016061777746 2.373688212059799 2.626281855617183 1.2941098998693366 2.5986003604328123
32 1.8325375178333774 1.5547338791128371 1.2926123104989153 2.6827802670732206 1.2229297061593511
2.5399309281771143 1.118405799650005 2.369208547545182 1.0765962370462665 2.205454427347206 1.0103977629236804
2.093962260403903 1.0069136327067023 1.9336922704229058 1.0765962370462665 1.7769064106588863
1.1497629716028088 1.5957316393760195 1.1985407946405038 1.4006203472252399 1.2055090550744603
1.1985407946405038 1.2229297061593511 1.0313025442255497 1.2960964407158937 0.8257388614238355
1.351842524187545 0.6306275692730557 1.4737870817817822 0.5017147512448621 1.651477722847671
0.4424845375562325 1.9441446610738404 0.4703575792920582 2.086993999969947 0.45990518864112356
2.268168771252814 0.46338931885810175 2.407533979931942 0.5365560534146442 2.5294785375261797
0.7769610383861405 2.571288100129918 1.0731121068292884 2.5643198396959614 1.404104477442218
2.5782563605638744 1.6898031552344313 2.640970704469482 1.9162716193380147 2.6758120066392643
2.1427400834415984 2.6235500533845912 2.2472639899509446 2.5399309281771143 2.3587561568942474
2.487668974922441 2.5085737562243104 2.3901133288470513 2.6235500533845912 2.3134624640735306
2.7420104807618504 1.2926123104989153 2.6827802670732206 NA NA NA NA NA NA NA NA NA NA NA NA
36 1.8728428768974725 1.3135129152864806 1.3298530945632818 2.5972065658833805 1.2465202635723711
2.440957507775423 1.1492986274163088 2.274291845793602 1.0729101990079741 2.1284593915595087
0.9895773680170635 1.9930435411992788 0.9965217705996394 1.8263778792174576 1.0555491925515343
1.6354068081966207 1.1006878093382777 1.496518756545103 1.138882023542445 1.3298530945632818
1.1597152312901726 1.1562430299988846 1.1458264261250208 0.9965217705996394 1.1597152312901726
0.7986062969962267 1.2083260493682038 0.6145796285579658 1.2916588803591145 0.4270807588284169
1.4027693216803285 0.2638872981378836 1.5312407694579824 0.16319346069053328 1.6249902043227569
0.1319436490689418 1.8090168727610179 0.1319436490689418 1.975682534742839 0.1562490581079574
2.215264423841707 0.15277685681666944 2.4305409039015595 0.22569308393371623 2.6215119749223965
0.4062475510806892 2.6979004033307312 0.6354128363056935 2.7360946175348984 0.8576337189481217
2.7291502149523224 1.1145766145034295 2.694428202039443 1.2916588803591145 2.7256780136610343
1.5312407694579824 2.809010844651945 1.795128067595866 2.812483045943233 2.0104045476557184 2.711789208495883
2.1562370018898123 2.642345182670124 2.3159582612890572 2.5555401503879254 2.4340131051928475
2.4860961245621667 2.520818137475046 2.4201243000276955 2.6492895852526996 1.3298530945632818
2.5972065658833805 NA NA NA NA
39 1.993031860059195 1.2119778986509275 1.4186511503893575 2.4566885775035217 1.3494486552484133
2.32174371197868 1.262945536322233 2.204099470239075 1.2006632906953831 2.0587742304430923 1.131460795554439
1.9411299887034867 1.0830190489557778 1.8027249984215983 1.1141601717692027 1.6124181367840016
1.1764424173960526 1.4151910256323104 1.2179639144806191 1.2491050372940442 1.231804413508808
1.0553380508994001 1.2248841639947137 0.8615710645047562 1.2491050372940442 0.6643439533530651
1.297546783892705 0.47403709171546826 1.3632891542766021 0.26988973104968267 1.4497922732027826
0.1799264873664551 1.6089580120269542 0.11764424173960526 1.730062378523607 0.10034361795436919
1.9826514857880533 0.13840499028188855 2.1764184721826974 0.10034361795436919 2.32174371197868
0.11072399222551084 2.460148702260569 0.15916573882417182 2.512050573616277 0.2076074854228328
2.605473942056552 0.32525172716243805 2.6642960629263546 0.5363193373423181 2.6815966867115906
0.7093255751946788 2.702357435253874 0.9584545577020782 2.685056811468638 1.1280006707973915
2.6919770609827323 1.2837062848645162 2.7611795561236763 1.5016941445584906 2.8130814274793847
1.6920010061960875 2.8303820512646207 1.8442464955061648 2.7992409284511957 1.9238293649182507
2.7161979342820626 2.0518539809289975 2.6573758134122603 2.204099470239075 2.5743328192431267
2.304443088193444 2.5328113221585604 2.4013265813907663 2.463608827017616 2.477449326045805 1.4186511503893575
2.4566885775035217
37 1.7229166666666667 1.3579375 1.1597222222222223 2.6805555555555554 1.0833333333333333 2.517361111111111

1.0034722222222223 2.4097222222222223 0.9444444444444444 2.2881944444444446 0.8993055555555556
2.1770833333333335 0.8159722222222222 2.0347222222222223 0.8298611111111112 1.8645833333333333
0.8888888888888888 1.6701388888888888 0.9409722222222222 1.5381944444444444 0.9791666666666666
1.3541666666666667 0.96875 1.1840277777777777 0.9618055555555556 1.0 0.9930555555555556 0.7916666666666666
1.0555555555555556 0.5590277777777778 1.1423611111111112 0.3472222222222222 1.3194444444444444
0.21180555555555555 1.4895833333333333 0.1597222222222222 1.6111111111111112 0.16319444444444445
1.8090277777777777 0.1840277777777778 1.9965277777777777 0.1875 2.1944444444444446 0.21875 2.3125
0.2777777777777778 2.4097222222222223 0.3368055555555556 2.5381944444444446 0.5972222222222222
2.5659722222222223 0.8020833333333334 2.6041666666666665 1.03125 2.5694444444444446 1.3333333333333333
2.6006944444444446 1.5347222222222223 2.6597222222222223 1.8020833333333333 2.6805555555555554
2.0416666666666665 2.625 2.1770833333333335 2.53125 2.329861111111111 2.454861111111111 2.4722222222222223
2.3715277777777777 2.5555555555555554 2.2916666666666665 2.6944444444444446 1.1597222222222223
2.6805555555555554 NA NA
37 1.7759823496757043 1.4958243162868454 1.2152704519507798 2.63192857879626 1.142354224833733
2.4791517219795907 1.0520769912602466 2.357624676784513 1.013882777056079 2.2534586380458745
0.9444387512303203 2.163181404472388 0.9027723357348649 2.0138767489470064 0.9201333421913047
1.8749886972954888 0.9791607641431996 1.7013786327310916 1.0277715822212308 1.5520739772057102
1.0659657964253981 1.427074730719344 1.0590213938428223 1.2499924648636591 1.0624935951341103
1.0069383744735032 1.135409822251157 0.7673564853746352 1.2256870558246435 0.5763854143537984
1.3437418997284336 0.42360855753712895 1.5277685681666946 0.38194214204167365 1.7117952366049554
0.36110893429394597 1.798600268887154 0.3645811355852339 1.9999879437818546 0.3715255381678098
2.1840146122201154 0.4409695639935686 2.347208072910649 0.5659688104799345 2.451374111649287
0.7604120827920593 2.4895683258534547 0.9131889396087288 2.520818137475046 1.0972156080469897
2.5034571310186062 1.2881866790678265 2.4965127284360302 1.4617967436322237 2.4965127284360302
1.5972125939924533 2.538179143931486 1.7708226585568505 2.6006787671746685 1.9965157424905666
2.5972065658833805 2.1145705863943567 2.5173459361837582 2.2395698328807225 2.4340131051928475
2.3715134819496644 2.3367914690367853 2.4965127284360302 2.281236248376178 2.6041509684659565
2.2360976315894345 2.677067195583003 1.2152704519507798 2.63192857879626 NA NA
39 1.7881607315303831 1.4446191273137365 1.3296936038777876 2.6804377661283105 1.2700503551550373
2.5506259894964423 1.1893565480595514 2.445373197632765 1.1191880201504334 2.336611979373632
1.0630531978231388 2.234867613905411 0.9683256851458294 2.1015474108780863 0.9542919795640058
1.933142943896203 0.9893762435185649 1.8349070048234377 1.0279689338685798 1.7331626393552164
1.0805953298004183 1.5928255835369802 1.0946290353822419 1.4384548221369207 1.091120608986786
1.2735587815504932 1.0630531978231388 1.136730152127713 1.091120608986786 0.9332414211912704
1.1507638577095365 0.7051937054866366 1.1823396952686396 0.543806091295665 1.2875924871323168
0.3754016243137817 1.3963537053914497 0.29119939082284 1.5226570556278622 0.27014883245010457
1.641943553073363 0.23857299489100145 1.8419238576143495 0.26313197965919277 2.013836750991689
0.21752243651826603 2.2489013194872345 0.26664040605464867 2.445373197632765 0.3929437562910612
2.56115126868281 0.6209914719956949 2.634828222987384 0.8104464973503138 2.6488619285692074 1.0665616242185947
2.631319796591928 1.3577610150414348 2.6488619285692074 1.5787918779551566 2.726047309269237
1.7787721824961433 2.7821821315965316 1.971735634246218 2.77516527880562 2.1225979692508217 2.6909630453146782
2.2173254819281314 2.6137776646146484 2.368187816932735 2.5190501519373387 2.4839658879827797
2.4488816240282207 2.6032523854282807 2.4173057864691176 2.6804377661283105 1.3296936038777876
2.6804377661283105
37 1.8723136125344095 1.4473122148545685 1.4275098261312074 2.689511266624014 1.3309632678421401
2.541243337822946 1.234416709553073 2.403319683124279 1.1930396131434728 2.3067731248352117 1.1171816030592057
2.1998822924437444 1.0413235929749387 2.086095277317344 1.0240831361376053 1.9861006276608102
1.0413235929749387 1.9412754398837433 1.0861487807520056 1.7964556024501426 1.1275258771616057
1.6033624858720081 1.1620067908362726 1.4344060088661406 1.1654548822037394 1.2482090750229398
1.148214425366406 1.0482197757098721 1.1551106081013391 0.851678567764271 1.1964877045109394
0.6723778166560035 1.2551052577578732 0.42756332956586884 1.3930289124565405 0.2655030352949347
1.5481930239925412 0.18274884247573428 1.7205975923658754 0.18274884247573428 1.9481716226186767
0.2172297561504011 2.1550571046666778 0.16206029427093416 2.392975409021879 0.18964502521066764

2.6033089824373468 0.3896343245237353 2.6998555407264138 0.6585854511861367 2.7688173680757475
0.9033999382762713 2.7584730939733473 1.1757991563061394 2.7619211853408143 1.4378541002336074
2.8239868299552144 1.6516357650165419 2.9101891141418816 1.879209795269343 2.9136372055093482
2.030925815437877 2.8032982817504144 2.1998822924437444 2.7619211853408143 2.3102212162026783
2.717095997563747 2.3964235003893455 2.59986089106988 2.54813952055788 2.544691429190413 2.6377898961120136
1.4275098261312074 2.689511266624014 NA NA
38 1.7385678437120404 1.3744585556800302 1.2055675948236897 2.616708854660667 1.1498188043116115
2.4877897766014865 1.104522912020548 2.4111351896473794 1.0069625286244113 2.2787318121811935
0.9790881333683722 2.153297033529018 0.8884963487862452 2.0417994525048617 0.8606219535302062
1.9128803744456808 0.8989492470072599 1.79092989520051 0.9407608398913184 1.6585265177343245 0.986056732182382
1.4947644456050948 1.0174154268454259 1.3275180740688606 1.0243840256594356 1.0975543132065382
1.0313526244734454 0.8153260612391426 1.0836171155785186 0.6202052944468692 1.19163039719567
0.4390217252826153 1.3031279782198264 0.31707124603744435 1.4982487450120998 0.24041665908333695
1.6271678230712805 0.23693235967633205 1.7735083981654856 0.2508695573043516 1.9477233685157298
0.23344806026932718 2.0243779554698373 0.23693235967633205 2.1637499317500324 0.28222825196739554
2.362354997949311 0.39024153358454694 2.4668839801594573 0.5644565039347911 2.4982426748225013
0.7491243725060499 2.53308566889255 0.9930253309963917 2.5296013694855453 1.2613163853357678
2.5226327706715357 1.4355313556860119 2.553991465334579 1.6201992242572707 2.595803058218638
1.7978984940145197 2.6341303516956915 1.9755977637717688 2.606255956439653 2.094063943609935
2.5191484712645305 2.1916243270060716 2.439009584903418 2.3484178003212914 2.355386399135301 2.480821177787477
2.2647946145531743 2.6341303516956915 1.2055675948236897 2.616708854660667
44 1.7407196829870777 1.3076219297062075 1.2041450405201313 2.6781846590878784 1.1107199942728798
2.5432373700640705 1.0103745742295356 2.4082900810402625 0.927330088676423 2.2975641003027794
0.8339050424291714 2.1729973719731106 0.7577809306721516 2.058811204337581 0.7785420520604297
1.8858018594352632 0.8339050424291714 1.7300934490231772 0.8927282196959595 1.5501637303247668
0.9238699017783767 1.4705794316697007 0.9342504624725158 1.3598534509322173 0.8892680327979131
1.2525876570927803 0.868506911409635 1.1141801811709262 0.868506911409635 0.9584717707588402
0.9169495279822839 0.761241117570198 0.9342504624725158 0.6020725202600656 0.9792328921471183
0.42560298845970157 1.07265793839437 0.2525936435573839 1.179923732233807 0.20761121388278125 1.40483588060682
0.16262878420817867 1.5640044779169522 0.1972306531886422 1.7058721407368527 0.24567326976129117
1.844279616658707 0.32871775531440367 1.982687092580561 0.2525936435573839 2.214519614749667
0.1972306531886422 2.404829894142216 0.2283723352710594 2.5328568093699313 0.31487700772221827
2.605520734228905 0.3944613063772844 2.667804098393739 0.5259484085030459 2.730087462558574 0.878887472103774
2.730087462558574 1.0138347611275818 2.712786528068342 1.2249061619084094 2.667804098393739 1.3702340116263563
2.674724472189832 1.5363229827325813 2.737007836354666 1.73701382281927 2.7785300791312224 1.934244476007912
2.7750698922331765 2.128014942298508 2.69548559357811 2.2595020444242695 2.595140173534766 2.3944493334480774
2.47749381900119 2.5155558748797 2.3771483989578455 2.6332022294132758 2.3148650347930113 2.7162467149663883
1.2041450405201313 2.6781846590878784
39 1.7814760894133412 1.361822054363254 1.2278840536321542 2.479577493345819 1.139449191043689
2.367333244675843 1.1020344414870304 2.278898382087378 1.0067968971609909 2.2176742464492096
0.9319673980476739 2.102028656910447 0.8333285128528471 1.9829817265028975 0.8095191267713372
1.8571321143577737 0.8639405806719314 1.6734597074432684 0.9013553302285897 1.530603390954209
0.9557767841291839 1.3571350066460652 0.9183620345725254 1.1734625997315602 0.9183620345725254
0.9455727615228224 0.9523754432603967 0.7584990137395302 0.9897901928170552 0.5680239250874509
1.0986331006182433 0.3673448138290102 1.2618974623200256 0.23809386081509917 1.452372550972105
0.1972777703896536 1.6394462987553973 0.22448849733995066 1.8469280917514121 0.3163247007972032
2.0408045212722787 0.24489654255267343 2.2278782690555707 0.24829788342146059 2.380938608150992
0.32312738253477746 2.510189561164903 0.4829904033677726 2.56120967419671 0.6564587876759163 2.602025764622155
0.8129204676401244 2.6394405141788138 0.9353687389164611 2.6564472185227492 1.1360478501749018
2.625835150703665 1.5442087544293575 2.646243195916388 1.6734597074432684 2.693861968079408 1.8231187056699023
2.7108686724233437 1.945566976946239 2.666651241129111 2.071416589091363 2.5850190602782197 2.1870621786301254
2.510189561164903 2.275497041218591 2.3911426307573533 2.384339949019779 2.340122517725546 2.46597212987067
2.275497041218591 2.5408016289839868 1.2278840536321542 2.479577493345819

41 1.888452380952381 1.5284319727891156 1.316326530612245 2.697278911564626 1.2312925170068028
2.5714285714285716 1.1938775510204083 2.5170068027210886 1.08843537414966 2.4081632653061225
1.0034013605442176 2.2687074829931975 0.9319727891156463 2.1258503401360542 0.9659863945578231 2.0
1.030612244897959 1.8129251700680271 1.0748299319727892 1.6360544217687074 1.08843537414966 1.4727891156462585
1.0816326530612246 1.2585034013605443 1.1258503401360545 1.0170068027210883 1.1564625850340136
0.8605442176870748 1.1870748299319729 0.7142857142857143 1.2585034013605443 0.5816326530612245
1.3979591836734695 0.5034013605442177 1.5578231292517006 0.4965986394557823 1.7142857142857142
0.5306122448979592 1.8605442176870748 0.6224489795918368 1.9591836734693877 0.6904761904761905
2.1258503401360542 0.5918367346938775 2.261904761904762 0.54421768707483 2.391156462585034 0.5340136054421769
2.5170068027210886 0.6054421768707483 2.622448979591837 0.7448979591836735 2.6598639455782314
0.8809523809523809 2.697278911564626 1.0816326530612246 2.7108843537414966 1.3639455782312926
2.683673469387755 1.564625850340136 2.663265306122449 1.6870748299319729 2.683673469387755 1.836734693877551
2.7244897959183674 2.0170068027210886 2.7312925170068025 2.1904761904761907 2.6904761904761907
2.292517006802721 2.6156462585034013 2.3741496598639458 2.554421768707483 2.4829931972789114 2.442176870748299
2.5884353741496597 2.3639455782312924 2.6802721088435373 2.2857142857142856 2.761904761904762
1.316326530612245 2.697278911564626
38 1.8467606847908704 1.5091415352030884 1.3597280815728094 2.619476157147618 1.3130707454404091
2.5228288180162175 1.2497500749750088 2.442844813217817 1.143104735243808 2.3661934752860168
1.0931147322448076 2.2462174680884157 1.0097980605798071 2.156235462690215 0.9798040587804069
2.012930787426414 1.016463394313007 1.8229687760302127 1.0564553967122075 1.692994768232812 1.0964473991114077
1.5030327568366106 1.0464573961124073 1.143104735243808 1.0764513979118076 1.003132726846607
1.1164434003110077 0.7598480455848053 1.1864294045096082 0.5932147022548041 1.309738078573809
0.45990802759080324 1.4730387550372104 0.4665733613240033 1.6496700989670117 0.5332266986560037
1.7529827718316122 0.6032127028546043 1.8063054416972126 0.6398720383872045 1.9596081175608138
0.5398920323892038 2.2128907994224156 0.4532426938576032 2.426181478884817 0.5032326968566035
2.559488153548818 0.6632007064534047 2.592814822214818 0.853162717849606 2.6261414908808183 1.0397920623792072
2.6528028258136187 1.2297540737754087 2.6428048252138185 1.3597280815728094 2.619476157147618
1.6829967676330118 2.6661334932800185 1.876291445895813 2.722788830012219 2.0229287880262143 2.716123496279019
2.142904795223815 2.622808824014218 2.2762114698878158 2.5261614848828176 2.402852810818617 2.442844813217817
2.4961674830834175 2.399520143952017 2.559488153548818 2.3595281415528166 2.6394721583472185
1.3597280815728094 2.619476157147618
39 2.0375927182081854 1.3411216355105107 1.4980606703215347 2.6387857679058224 1.3743675874509491
2.480733495348963 1.2506745045803638 2.3364248986666136 1.1544687734587973 2.1989881399215188
1.0616989613058583 2.082166894988188 1.0479552854313487 1.8863195137764277 1.1063659078980141
1.6732925377215306 1.1544687734587973 1.4774451565097704 1.1647765303646793 1.3090851270470292
1.123545502741151 1.133853259647033 1.1544687734587973 0.9380058784352728 1.1682124493333068
0.8211846335019422 1.254110423548991 0.580670305698026 1.3331365598274207 0.40543843829803 1.4946247513529072
0.27143759852156246 1.632061510098002 0.2508220847097982 1.7488827550313328 0.27487351749018984
1.8382166482156446 0.3264123020196004 1.9756534069607394 0.41574619520391215 2.1440134364234806
0.2954890313019541 2.3707840883528872 0.26112984161568037 2.6387857679058224 0.32297638305097304
2.755607012839153 0.4432335469529311 2.8311972301489554 0.6734401178509651 2.865556419835229 0.893338931843117
2.9274029612705217 1.1201095837725237 2.8999156095215026 1.233494909737227 2.8793000957097385
1.4430859668234965 2.9067874474587576 1.611445996286238 2.9583262319881682 1.8038574585293707
3.002993178580324 1.9584738121176026 2.9961213406430693 2.064987300145051 2.923967042301894 2.1818085450783817
2.8243253922117004 2.2917579520744575 2.7349914990273887 2.3982714401019063 2.645657605843077
2.4944771712234726 2.5494518747215107 2.6284780109999404 1.4980606703215347 2.6387857679058224
39 2.0535194266876307 1.6844990791948997 1.5153734070428782 3.0076700616942404 1.3999896450852987
2.846132794953629 1.2961442593234769 2.7422874091918072 1.1769143719673114 2.6038268948427117
1.0961457385970057 2.4961353836823044 1.030761606821044 2.3769054963261387 1.023069356023872 2.226906605781285
1.061530610009732 2.111522843823706 1.1307608671842797 1.8884475707057187 1.1884527481630693
1.7692176833495532 1.1615298703729675 1.6346032943990436 1.1230686163871075 1.4346047736725724
1.1307608671842797 1.2192217513517574 1.1884527481630693 0.9307623464578084 1.2499907545404452
0.6692258193539614 1.3807590180923686 0.503842427214764 1.6115265420075278 0.4307660446416303

1.803832811936827 0.4653811732289042 2.007677458061884 0.5499959319977958 2.2615217343685594
0.4576889224317322 2.55767339005968 0.4307660446416303 2.7422874091918072 0.52691917960628 2.861517296547973
0.6923025717454773 2.8961324251352467 0.8769165908776045 2.9269014283239345 1.0346077322196299
2.9461320553168644 1.1192224909885216 2.9730549331069667 1.530757908637222 2.9499781807154504
1.6384494197976296 2.9461320553168644 1.8192173135311709 3.0384390648829283 2.08844609143219 3.080746444267374
2.238444981977043 3.080746444267374 2.357674869333209 2.9999778108970685 2.4769047566893745 2.9269014283239345
2.619211396437056 2.826902167960699 2.7076722806045335 2.746133534590393 2.823056042562113 2.642288148828572
2.9807471839041386 1.5153734070428782 3.0076700616942404
37 2.173590486618477 1.45456972450769 1.551314279461674 2.880465032481923 1.4555541387541633
2.734909618606507 1.309998724878747 2.558710959704687 1.1721041222599315 2.3825123008028672 1.0801743871807212
2.210144047529348 1.0993264153222233 1.9726588985747213 1.1567824997467298 1.7849690227880002
1.2180689897995367 1.5628054963465754 1.24871223482594 1.3789460261881548 1.1874257447731331
1.1606129053750303 1.2372210179410388 0.9307885676770045 1.283185885480644 0.7507595031468842
1.363624403674953 0.48646151479415456 1.4747061668956654 0.3638885346885408 1.685378476452189
0.2796196108659314 1.8462555128408071 0.2834500164942318 2.0262845773709275 0.3217540727772361
2.148857557476541 0.39070137408664385 2.397833923316069 0.2949412333791331 2.646810289155597
0.2719587996093305 2.895786654995125 0.3638885346885408 3.0260204463573395 0.5094439485639571
3.0987981532950477 0.7086250412355795 3.1102893701799488 0.900145322650601 3.1294413983214513
1.0610223590392192 3.1332718039497514 1.3866068374447555 3.0911373420384467 1.5972791470012793
3.1141197758082493 1.8117818621861033 3.171575860232756 2.0569278223973306 3.2060495108874596
2.2331264812991507 3.1332718039497514 2.3825123008028672 3.022190040729039 2.558710959704687 2.868973815597022
2.6774535341820003 2.7847048917744126 2.7847048917744126 2.700435967951803 2.876634626853623 1.551314279461674
2.880465032481923
37 1.9812623016012767 1.4799666554655444 1.4999892390965144 2.9014943362321466 1.3674144326107114
2.742404568449183 1.2234760712832682 2.5984662071217395 1.1174162260946257 2.450739994180416
1.0227199357476235 2.306801632852973 1.007568529292103 2.140136161842249 1.0302956389753837 1.9772585424454054
1.1022648196391054 1.7537752972264802 1.136355484164026 1.5568070133047158 1.132567632550146
1.4052929487495123 1.0833255615697048 1.2234760712832682 1.1174162260946257 1.0340834905892637
1.1855975551444673 0.8522666131230195 1.2348396261249084 0.6060562582208139 1.3371116196996706
0.42423938075456974 1.5037770907103944 0.31060383233816713 1.6704425617211183 0.3143916839520472
1.8030173682069215 0.3522702000908481 1.9507435811482448 0.43181508398232993 2.136348310228369
0.34090664524920783 2.3408922973778936 0.30681598072428706 2.5946783555078596 0.30681598072428706
2.7651316781324633 0.39393656784352904 2.890130781390506 0.5454506323987325 2.9431607039848275
0.8219638002119789 2.996190626579149 1.1818097035305872 2.973463516895868 1.4848378326409941 2.988614923351389
1.7348360391570798 3.0605841040151103 1.9961978005148058 3.087099065312271 2.2007417876643305
3.0302812911040697 2.3371044457640138 2.912857891073787 2.5075577683886174 2.784070936201864
2.6401325748744204 2.696950349082622 2.7764952329741037 2.61740546519114 2.840888710410065 1.4999892390965144
2.9014943362321466
34 1.9544445349937594 1.3161720863532294 1.5286907512129575 2.5286613929838397 1.4444020381134963
2.4137222387573014 1.3409567993096119 2.3141083050943014 1.2298489502239582 2.176181320022456
1.1149097959974201 2.0305917246688407 1.0995845754338818 1.8811708241743412 1.1187411011383048
1.7317499236798417 1.1685480679698046 1.5363533614947267 1.1723793731106893 1.2643306964919199
1.1570541525471507 1.0651028291659204 1.1800419833924585 0.8390558258537286 1.2145237296604199
0.6398279585277291 1.2758246119145735 0.44826270148349884 1.4482333432543808 0.32182963183430685
1.601485548889765 0.2950104958481146 1.758569059666034 0.3294922421160761 1.9156525704423026
0.40228703979288355 2.183843930304225 0.2950104958481146 2.4252161541799553 0.31033571641165303
2.582299664956224 0.39462442951111437 2.658925767773916 0.544045330005614 2.685744903760108 0.7011288407818828
2.7010701243236466 0.8313932155719593 2.7393831757324927 1.1608854576880354 2.72022665002807
1.3639446301549194 2.7623710065778004 1.5478472769173806 2.8313344991137233 1.7432438391024954
2.854322329959031 1.9156525704423026 2.796852752845762 2.068904776077687 2.7010701243236466 2.1991691508677635
2.612950106083301 2.337096135939609 2.517167477561186 2.4865170364341087 1.5286907512129575 2.5286613929838397
NA NA NA NA NA NA
33 1.799290851987643 1.3860960821337966 1.334418308444367 2.662147803312572 1.2273972912257713

2.5116494978489214 1.0969654264906075 2.384562039901839 0.9799111889077683 2.2374081412262696
0.8963010192057403 2.100287462914944 0.8829233920534159 1.9631667846036178 0.9230562735103893
1.7792244112591562 0.9665335617554438 1.585248817550451 1.0033220364243363 1.4180284781463952
0.9464671210269572 1.110343053642932 0.9799111889077683 0.8695457649010914 1.023388477152823
0.6153708490069262 1.1203762740071754 0.3812623738412477 1.307663054139718 0.277585763410733
1.5016386478484232 0.26086372947032743 1.665514580464398 0.3110298312915442 1.8227016995042107
0.3912955942054911 2.026710513577159 0.277585763410733 2.2842298362594056 0.24748610231800292
2.501616277484678 0.3578515263246799 2.635392549007923 0.5685491539737905 2.668836616888734 0.7625247476824955
2.7256915322861133 1.0300772907289852 2.6922474644053023 1.3912732238417462 2.7390691594384378 1.6120040718551
2.8360569562927904 1.9029674624181576 2.8260237359285467 2.0768766153983758 2.722347125498032
2.247441361590513 2.555126786093976 2.4313837349349745 2.471516616391948 2.571848820034382 2.4146617009945692
2.632048142219842 1.334418308444367 2.662147803312572 NA NA NA NA NA NA NA NA
36 1.8297071148481736 1.3969282709666044 1.3590297956287498 2.44625363213175 1.2684278092535
2.2952503215063333 1.1476251607531667 2.1643807856309722 1.0570231743779166 2.0133774750055555
1.010044366627787 1.8959304556302314 1.0637344326279352 1.748282774129824 1.0939350947530184
1.5771456887543518 1.140913902503148 1.439564894628972 1.1610476772532037 1.2852059548785462
1.1342026442531297 1.1442695316281573 1.154336419003185 0.9630655588776573 1.1845370811282685
0.8221291356272684 1.2214490015033703 0.6979708580019259 1.2852059548785462 0.5402562891264907
1.4462761528789907 0.4328761571261944 1.6006350926294166 0.43958741537621293 1.7315046285047777
0.48656622312634257 1.8724410517551666 0.5436119182515 2.046933766255648 0.4764993357513148 2.26504965938125
0.466432448376287 2.432831115631713 0.5603900638765462 2.5200774728819537 0.7885728443771759
2.5569893932570555 0.9429317841276018 2.5871900553821385 1.140913902503148 2.526788731131972
1.4563430402540185 2.5569893932570555 1.5469450266292684 2.5939013136321574 1.7180821120047407
2.634168863132268 1.8959304556302314 2.6207463466322314 2.0301556206306017 2.526788731131972 2.124113236130861
2.4663874068818052 2.228137739006148 2.382496678756574 2.305317208881361 2.3019615797563517 2.405986082631639
2.2583384011312315 2.473098665131824 1.3590297956287498 2.44625363213175 NA NA
42 1.9058999520971311 1.3344638392530241 1.410826537778409 2.7463176779084564 1.304672113819354
2.6367389176926577 1.2087906986305301 2.527160157476859 1.0957876021579875 2.4004597159773415
1.0204522045096258 2.29772962827503 0.9382681343477769 2.208696885599694 0.9279951255775457 2.0717234353299454
0.9553898156314954 1.8868092774657848 1.006754859482651 1.6813491020611622 1.054695567077063 1.469040254143052
0.9930575144556762 1.338915476386791 0.9793601694287014 1.1711229998063493 1.0033305232259073
0.9382681343477769 1.0512712308203194 0.7362322951998979 1.0889389296445002 0.5273477835385315
1.160849991036118 0.3698283157283208 1.2875504325356353 0.2705225642827532 1.4484942366025897
0.23285486545857237 1.6060137044128004 0.2465522104855472 1.7395628184258052 0.284219909309728
1.8902336137225286 0.34928229818785855 2.0546017540462267 0.24997654674229092 2.2463645844238744
0.20546017540462266 2.4175813972610602 0.2191575204315975 2.5751008650712706 0.29449291807995914
2.6744066165168383 0.4074960145525016 2.7291959966247377 0.5444694648222501 2.7565906866786873
0.6814429150919985 2.783985376732637 0.8423867191589529 2.845623429354024 1.1300309647254247
2.8285017480703054 1.314945122589585 2.797682721759612 1.4313725553188712 2.845623429354024 1.6094380406695443
2.903837145718667 1.8080495435606794 2.965475198340054 2.027207063992277 2.931231835772617 2.1710291867755127
2.845623429354024 2.308002637045261 2.7223473241112504 2.455249096085241 2.62989024517917 2.5785252013280147
2.5100384761931402 2.7086499790842753 1.410826537778409 2.7463176779084564
34 1.7419419334703061 1.2359910610010814 1.254969965071793 2.3589408701750547 1.1878592717524457
2.2280750182023272 1.0670600237776207 2.103920235561535 0.9462607758027957 1.9562767102589713
0.8892166864813505 1.7314558876391581 0.9261275678069916 1.5200572036832143 0.9831716571284367
1.348924935718879 1.0267936077860123 1.2482588957398582 0.9630384491326325 1.0804821624414902
0.9764605877965019 0.8657279438195791 1.013371469122143 0.7113733491850804 1.0603489544456859
0.5234634078909083 1.1576594597587395 0.3389090012627034 1.3287917277230747 0.26508723861142147
1.5502570156769206 0.26508723861142147 1.6811228676496477 0.31877579326689925 1.7650112342988318
0.37917541725431175 1.9596322449249386 0.26508723861142147 2.197875206208621 0.25837616927948676
2.3690074741729568 0.3925975559181812 2.4361181674923036 0.5838630318783208 2.499873326145684
0.8120393891641012 2.5267176034734224 1.013371469122143 2.4830956528158468 1.191214806418413
2.5367842074713245 1.4495909756978997 2.587117227460835 1.6442119863240068 2.6072504354566393

1.8388329969501136 2.53007313813939 1.9596322449249386 2.446184771490206 2.083787027565731 2.325385523515381 2.1844530675447515 2.2549192955300663 2.2851191075237725 2.204586275540556 2.345518731511185 1.254969965071793 2.3589408701750547 NA NA NA NA NA NA
43 1.749594874481706 1.4040545796719412 1.322853800620002 2.67291282079003 1.2378374895261715 2.5742938999211864 1.166423788207354 2.479075631496096 1.057602910007251 2.3906586679585127 0.9895898611321866 2.268235179983397 0.918176159813369 2.1798182164458133 0.8671663731570707 2.067596685801957 0.870567025600824 1.9247692831643217 0.8909709402633432 1.8567562342892574 0.9283781171446287 1.618710563226532 0.9725865989134205 1.4826844654764033 0.9657852940259141 1.3160524957324955 0.9249774647008755 1.152821178432341 0.9487820318071479 1.0031924709071995 0.9793879038009269 0.8399611536070449 0.9997918184634462 0.6903324460819034 1.026997038013472 0.5407037385567618 1.1018113917760428 0.4012769883628798 1.2378374895261715 0.3332639394878154 1.3772642397200534 0.31966132971280253 1.526892947245195 0.35366785415033475 1.6595183925515706 0.41487959813789266 1.7649386183079203 0.46929003723794416 1.9519745027143474 0.36046915903784116 2.213824740883345 0.2890554577190236 2.400860625289772 0.3468665492628283 2.5436880279274074 0.4760913421254506 2.587896509696199 0.5951141776568133 2.6219030341337315 0.7957526718382532 2.6695121683462766 0.9929905135759398 2.67291282079003 1.2412381419699248 2.6559095585712638 1.445277288595118 2.696717387896302 1.649316435220311 2.7307239123338345 1.7819418805266865 2.771531741658873 1.9689777649331135 2.7783330465463796 2.0777986431332165 2.706919345227562 2.2036227835520856 2.6389062963524976 2.3294469239709548 2.523284113264888 2.4484697595023173 2.441668454614811 2.553889985258667 2.3906586679585127 2.659310211015017 1.322853800620002 2.67291282079003
35 1.78705061264202 1.3631172210935694 1.317560048642433 2.5709312744022856 1.2331010711653538 2.4425536286371257 1.0912099890038611 2.2939058282774667 0.9999942933286158 2.1655281825123063 0.9121569567524536 2.0337721776480633 0.9155353158515367 1.8479624271984894 0.9763457796350337 1.7229631405324124 1.0135077297249484 1.5236399536865057 1.0337778843194474 1.317560048642433 1.0202644479231147 1.0168860888240316 1.023642807022198 0.8276979792753746 1.0337778843194474 0.6587800243212165 1.1418853754901086 0.4695919147725594 1.300668253147017 0.37499785999823093 1.4560727717048425 0.37161950089914775 1.6385041630553332 0.42567324648447835 1.7871519634149924 0.5067538648624742 2.0135020230535643 0.38175457819639724 2.1790416189086392 0.36824114180006456 2.3344461374664647 0.42229488738539517 2.432418551339876 0.5506725331505553 2.4662021423307077 0.6858068971138818 2.48985065602429 0.8175629019781251 2.540526042510537 0.9493189068423683 2.5236342470151216 1.3344518441378488 2.5641745562041196 1.527018312785589 2.6486335336811986 1.7871519634149924 2.6722820473747806 1.9526915592700673 2.614849942690367 2.1047177187288097 2.5303909652132877 2.2229602871967202 2.4189051149435437 2.347959573862797 2.35133793296188 2.4459319877362087 2.2972841873765497 2.513499169717872 1.317560048642433 2.5709312744022856 NA NA NA NA
37 1.9566972324526781 1.3119551740798323 1.298297625430073 2.586425817762783 1.1999931054888926 2.4474435654321485 1.0474516090284403 2.2847326358743323 0.9559267111521688 2.1220217063165165 0.8508425691460793 1.983039453985882 0.8576221912109883 1.833887768557884 0.8881304905030788 1.671176839000068 0.9186387897951692 1.583041752156251 0.9932146325091682 1.3932123343387992 0.9966044435416227 1.1864338613590746 0.9830451994118048 0.9389776559898962 1.0508414200608946 0.7016908837180813 1.1186376407099847 0.4135569459594489 1.2474504599432557 0.21016828401217894 1.5084659094422521 0.09491470890872597 1.7186341934544311 0.12542300820081648 1.942361721596428 0.22372752814199695 2.206766982127879 0.14237206336308897 2.4406639433672392 0.16610074059027047 2.6135443060224186 0.21355809504463344 2.766085802482871 0.3932180797647219 2.8203227790021432 0.5423697651927198 2.84066164519687 0.7491482381724444 2.857610700359143 0.9660961442495323 2.857610700359143 1.1220274517424391 2.8338820231319612 1.291518003365164 2.793204290742507 1.3966021453712536 2.8338820231319612 1.5999908073185236 2.881339377586324 1.840667390622793 2.9152374879108693 1.9796496429534274 2.884729188618779 2.071174540829699 2.779645046612689 2.206766982127879 2.661001660476782 2.3423594234260587 2.5254092191786017 2.4745620536917845 2.396596399945331 2.620323928087328 1.298297625430073 2.586425817762783
37 2.0041828665620436 1.3194462402450937 1.4778070926697504 2.4573235721067443 1.3993092563385627 2.3242185452842956 1.3139855211959675 2.2423077595474044 1.2115970390248532 2.085312086885029 1.1330992026936655 1.9829236047139145 1.1092085568537389 1.883948071948504 1.160402797939296 1.703061753446202 1.1911193425906303 1.5460660807838267 1.215009988430557 1.358353863470117 1.215009988430557 1.2013581908077418 1.2115970390248532 0.9795164794369939 1.2559653812990026 0.7337841222263195 1.3242243694130789

0.4982906132327565 1.4709811938583428 0.3549467381931964 1.66551930998346 0.31740429473045445
1.795211387400205 0.3583596875989002 1.8873610213542078 0.416379827495865 1.9795106553082107
0.4675740685814222 2.1501581255934012 0.3822503334388269 2.3446962417185184 0.344707889976085
2.5494732060607475 0.3924891816559383 2.6655134858546767 0.5017035626384603 2.7303595245630494
0.6621121847065394 2.754250170402976 0.83275965499173 2.774727866837199 1.1126215062594427 2.771314917431495
1.2798560271389294 2.7474242715915684 1.4061351551499703 2.7679019680257912 1.59384737246368 2.808857360894237
1.7337782980975363 2.8463998043569787 1.901012818977023 2.8361609561398673 1.9965754023367297
2.754250170402976 2.081899137479325 2.658687587043269 2.2218300631131815 2.515343712003709 2.3583480393413336
2.4539106227010405 2.4675624203238558 1.4778070926697504 2.4573235721067443
33 1.823746917353453 1.2956672994513885 1.384321710729429 2.4183113914708207 1.2924871009267396
2.306069090600867 1.1700409545231538 2.1870242260418253 1.0884101902540966 2.0781832070164157
0.9931742986068631 1.9795460335246382 0.9727666075395988 1.84689604158742 0.9965755804514072
1.6632268219820414 1.0748050628759203 1.4217358110194136 1.0135819896741274 1.0986140357877288
1.0271871170523037 0.9217473798714381 1.0475948081195678 0.6632499596860901 1.108817881321361
0.48978458561434346 1.2890858190821957 0.326523057076229 1.4795576023766626 0.28230639309715627
1.6224114398475127 0.2959115204753325 1.775469122851995 0.3605358755216695 1.9897498790582704
0.26189870202989196 2.1972280715754575 0.26189870202989196 2.36729216380266 0.3401281844544052
2.4761331828280695 0.4999884311479756 2.5339549741853187 0.7652884150224116 2.5815729200089352
0.9999768622959512 2.567967792630759 1.2210601821913145 2.5747703563198474 1.3639140196621649
2.6461972750552722 1.5475832392675435 2.690413939034345 1.7652652773183628 2.6802100935007127
1.9217242421673892 2.5951780473871113 2.0577755159491513 2.5237511286516865 2.163215253130017
2.4217126733153647 2.2754575539999706 2.3536870364244837 2.3808972911808364 1.384321710729429
2.4183113914708207 NA NA NA NA NA NA NA NA
44 1.6225262841462842 1.4608884826804063 1.0405285185578639 2.782651184807838 0.9557949258414254
2.650466780170194 0.8337785523297542 2.50472500069792 0.732098241070028 2.3691512523516183 0.6338072735189595
2.2369668477139744 0.6338072735189595 2.1691799735408237 0.6575326794795622 1.9624300073127139
0.6948154602747951 1.823466915257755 0.7524343033219733 1.6268849801556178 0.776159709282576
1.4709751695573712 0.7287088973613705 1.3048973278331517 0.7388769284873431 1.0981473616050421
0.7931064278258637 0.9219014887548501 0.8134424900778089 0.7151515225267403 0.8541146145816993
0.5490736808025211 1.0066350814712886 0.4168892761648771 1.1218727675656448 0.3931638702042743
1.2472784847859737 0.4134999324562195 1.3828522331322752 0.46095074437742506 1.4845325443920012
0.5219589311332608 1.5794341682344124 0.5863564615977539 1.7319546351240014 0.49484418146400044
1.9522619761867415 0.4134999324562195 2.159011942414851 0.43044665099950724 2.2539135662572622
0.4846761503380278 2.352204533808331 0.593135149015069 2.409823376855509 0.7829383966998911 2.4267700953987967
0.9354588635894803 2.4504955013593994 1.0879793304790695 2.457274188776714 1.2540571722032887
2.4403274702334268 1.4370817324707958 2.409823376855509 1.5794341682344124 2.4030446894381936
1.7150079165807137 2.443716813942084 1.8370242900923852 2.474220907320002 1.918368539100166 2.53183975036718
2.128507849036933 2.5148930318238927 2.2674709410918923 2.4267700953987967 2.399655345729536 2.342036502682358
2.511503688115235 2.240356191422632 2.606405311957646 2.172569317249481 2.708085623217372 2.0912250682417004
2.7928192159338106 1.0405285185578639 2.782651184807838
40 1.878638181750189 1.2644658557580883 1.3897985686274315 2.542314454806277 1.2677674747967302
2.386385834911492 1.1423466283596204 2.264354741080791 1.0609925658058197 2.1253748842180475
0.9660794928263853 1.9762257695360794 0.9762487506456103 1.8406356652797446 1.0203155345289192
1.6813172927785511 1.0779413288378614 1.5186091676709494 1.0982798444763118 1.3559010425633478
1.0881105866570866 1.1999724226685629 1.0609925658058197 1.064382318412228 1.0813310814442698
0.8677766672405426 1.1355671231468036 0.6915095317073073 1.183023659636521 0.5220219013868889
1.2542084643710967 0.3898215497369625 1.3694600529889813 0.29151872415111198 1.505050157245316
0.2881289715447114 1.660978777140101 0.34575476585365367 1.7660611079387605 0.4203293231946378
1.87114343873742 0.49151412792921356 2.0236823060257967 0.3966010549497792 2.1728314207077646
0.3186367450023867 2.3694370718794504 0.2949084767575281 2.51519643395501 0.36948303409851224
2.616889012147261 0.4745653648971717 2.6711250538497953 0.62371447957914 2.691463569488245 0.816930378144417
2.7287508481587373 1.054213060593003 2.7185815903395123 1.2067519278813794 2.68129431166902 1.3389522795313058
2.7084123325202873 1.55928619894785 2.7558688690100044 1.8033483866092526 2.80671515810613 1.983005274748896

2.7592586216164126 2.091477358153964 2.6779045590626116 2.227067462410299 2.566042723051136 2.3321497932089583
2.5084169287421934 2.4101141031563507 2.440621876614026 2.484688660497335 1.3897985686274315 2.542314454806277
41 1.762498477949425 1.264811610523942 1.2135314330941962 2.688073816881837 1.10166959708272
2.5389247021998687 0.9762487506456103 2.3829960823050835 0.8372688937828673 2.2474059780487488
0.752525078622658 2.105036368579597 0.7389660681970245 1.9524975012912207 0.78981235729315 1.7558918501195353
0.8338791411764589 1.5558964463414415 0.8677766672405426 1.41352683687229 0.8203201307508254
1.2643777221903219 0.8101508729316003 1.0948900918699034 0.8203201307508254 0.9559102350071602
0.8847254302725844 0.7423558208034329 0.8915049354854011 0.5457501696317475 0.9152332037302597
0.40338056016259594 1.0474335553801861 0.27118020851266955 1.203362175274971 0.19321589856527704
1.4609833733620072 0.16948763032041847 1.633860756288834 0.220333919416544 1.806738139215661
0.2982982293639365 1.9660565117168542 0.2101646615973189 2.1219851316116394 0.1762671355332352
2.277913751506424 0.18643639335246032 2.4270628661883924 0.2576211980870361 2.5558734652319104
0.4237190758010462 2.579601733476769 0.5626989326637893 2.623668517360078 0.7660840890482915
2.6846840642754284 0.9152332037302597 2.7016328273074706 1.0440438027737777 2.6677353012433866
1.2270904435198298 2.654176290817753 1.3965780738402482 2.7016328273074706 1.5864042197991168
2.7660381268292293 1.789789376183619 2.789766395074088 1.9863950273553044 2.7762073846484547 2.105036368579597
2.6779045590626116 2.277913751506424 2.566042723051136 2.4168936083691674 2.4609603922524763 2.532145196987052
2.3321497932089583 2.6677353012433866 1.2135314330941962 2.688073816881837
37 1.6359557823129252 1.311952380952381 1.1904761904761905 2.6700680272108843 1.0578231292517006
2.5136054421768708 0.9761904761904762 2.4149659863945576 0.8401360544217688 2.295918367346939
0.7517006802721088 2.163265306122449 0.6836734693877551 2.010204081632653 0.7142857142857143
1.8299319727891157 0.7551020408163265 1.6768707482993197 0.7687074829931972 1.58843537414966
0.7789115646258503 1.3537414965986394 0.7414965986394558 1.1972789115646258 0.7278911564625851
0.9659863945578231 0.7551020408163265 0.7346938775510204 0.7925170068027211 0.5 0.9081632653061225
0.2925170068027211 1.1462585034013606 0.1598639455782313 1.336734693877551 0.14625850340136054
1.5238095238095237 0.17687074829931973 1.6938775510204083 0.25170068027210885 1.870748299319728
0.16326530612244897 2.0918367346938775 0.15306122448979592 2.261904761904762 0.20408163265306123
2.4183673469387754 0.35374149659863946 2.489795918367347 0.6224489795918368 2.554421768707483
0.7959183673469388 2.5918367346938775 0.9285714285714286 2.598639455782313 1.0476190476190477
2.5816326530612246 1.4081632653061225 2.6360544217687076 1.6156462585034013 2.697278911564626
1.836734693877551 2.67687074829932 2.068027210884354 2.5850340136054424 2.248299319727891 2.435374149659864
2.4115646258503403 2.357142857142857 2.510204081632653 2.2687074829931975 2.608843537414966 1.1904761904761905
2.6700680272108843
PRONOTUM monticola rixosus simillimus 25 33 20 (78)
37 1.2104727143191123 1.4960629758740909 0.30636952187370975 1.3702461033252733 0.40063706706562047
1.3062788405164767 0.5521384789811912 1.2860786522610674 0.6800730045987843 1.2355781816225437
0.8349411145569233 1.1648775227286108 1.0066427147279036 1.0605098834089952 1.1951778051117248
0.9763424323447893 1.4005463857083875 0.8921749812805834 1.5587811937090947 0.8854415851954469
1.717016001709802 0.9258419617062658 1.8348504331996902 0.9898092245150623 1.9661516568598516
1.0874434677495413 2.080619390307172 1.1615108246860424 1.9526848646895787 1.1480440325157695
1.8281170371145539 1.1480440325157695 1.7978167547314396 1.2423115777076803 1.7405828880077796
1.4173798759212286 1.760783076263189 1.535214307411117 1.8079168488591444 1.7069159075820972
1.8651507155828044 1.8045501508165762 1.9964519392429658 1.902184394051055 1.858417319497668 2.016652127498375
1.6934491154118243 2.124386464860559 1.5217475152408442 2.1849870296267873 1.3332124248570227
2.1479533511585367 1.1177437501326555 2.057052504009194 0.942675451919107 1.9594182607747153
0.8214743223866503 1.8920842999233505 0.6699729104710795 1.8516839234125315 0.49827131030009936
1.8112835469017126 0.3703367846825063 1.7910833586463033 0.3164696160014145 1.7237493977949385
0.31310291795884626 1.6328485506455959 0.3164696160014145 1.5049140250280029 0.309736219916278
1.4173798759212286 0.30636952187370975 1.3702461033252733
30 0.9165402592838505 1.5526612326586167 0.19528508834396222 1.383830539816698 0.2996616010795282
1.3568946655623582 0.4646438308873584 1.3299587913080186 0.595956217877264 1.2659860899539621
0.7474705105579244 1.1683435457819809 0.8821498818296225 1.1111048129915093 1.050499095919245
1.080801954455377 1.2053803728816979 1.1010038601461318 1.346793712716981 1.1649765615001884

1.4545372097343394	1.2188483100088676	1.57911562816066	1.2659860899539621	1.4478032411707544	
1.3131238698990564	1.3703626026895281	1.4242343511982072	1.340059744153396	1.555546738188113	
1.3535276812805657	1.696960078023396	1.4410692726071694	1.8181715121679243	1.5690146753152827	
1.892245166367358	1.3636286341259432	1.9898877105393393	1.1952794200363206	2.0605943804569806	
1.0909029073007546	2.0807962861477356	0.8821498818296225	2.033658506202641	0.7272686048671697	
1.9461169148760373	0.6026901864408489	1.855208339267641	0.5016806579870754	1.794602622195377	
0.3670012867153773	1.7676667479410373	0.20201905690754712	1.720528967995943	0.18518413549858487	
1.6329873766693392	0.20201905690754712	1.444436256888962	0.19528508834396222	1.383830539816698	
31	1.0313319523317113	1.7751520318950489	0.17229631405324122	1.7297198587305787	0.23310677783673814
1.6385041630553332	0.42229488738539517	1.5844504174700027	0.5912128423395533	1.5135048763892562	
0.7364822836001291	1.4121541034167615	0.9121569567524536	1.2432361484626033	1.1182368617965264	
1.1689122482827738	1.300668253147017	1.131750298192859	1.5033697990920067	1.1283719390937759	1.672287754046165
1.1858040437781896	1.8412057090003229	1.2837764576516013	1.6689093949470817	1.3243167668405993	
1.5776936992718364	1.445937694407593	1.5574235446773375	1.6587743176498322	1.5844504174700027	
1.8513407862975724	1.6824228313434144	2.0135020230535643	1.8682325817929881	2.0810692050352273	
1.7837736043159091	2.239852082692136	1.550666826479171	2.395256601249961	1.3885055897231793	2.4189051149435437
1.172290607381857	2.3580946511600467	0.9155353158515367	2.2736356736829677	0.7195904881047134	
2.1891766962058887	0.5641859695468879	2.1283662324223918	0.418916528286312	2.1013393596297263	
0.314187396214734	2.091204282332477	0.2432418551339876	2.0743124868370613	0.20607990504407284	
1.989853509359982	0.18918810954865703	1.8243139135049071	0.17229631405324122	1.7297198587305787	
28	1.2538078787898082	1.700713578036241	0.3199897604914939	1.543950594371458	0.3959873286082237
1.471952898260872	0.6639787530198499	1.423954434187148	0.8919714573700392	1.311958018015125	1.0919650576772229
1.17596236980624	1.3639563540949928	1.0799654416587918	1.5839493144328949	1.0839653136649356	
1.7719432987216475	1.151963137769378	1.935938050973538	1.2359604498983952	2.0799334431947103	1.347956866070418
1.9439377949858254	1.411954818168717	1.9159386909428198	1.5599500823960328	1.8919394589059577	
1.7359444506663544	1.9159386909428198	1.9519375389981128	1.9959361310656931	2.039934723133274	
2.107932547237716	2.0919330592131415	1.8199417627953716	2.1999296033790205	1.5399507223653144	
2.3559246116186237	1.2999584019966939	2.315925891557187	1.0279671055789241	2.1919298593667333	
0.8519727373086025	2.095932931219285	0.6879779850567119	2.0279351071148426	0.49998400076795924	
1.983936515047262	0.355988608546787	1.9639371550165439	0.3159898884853502	1.8439409948322336	
0.3279895045037812	1.619948162488188	0.3199897604914939	1.543950594371458		
31	1.496031746031746	1.7783253968253967	0.5277777777777778	1.5753968253968254	0.7063492063492064
1.5119047619047619	0.9761904761904762	1.4126984126984128	1.1388888888888888	1.3253968253968254	
1.3293650793650793	1.2023809523809523	1.507936507936508	1.1587301587301588	1.7182539682539681	
1.1071428571428572	1.8888888888888888	1.1150793650793651	2.0674603174603177	1.2023809523809523	
2.1904761904761907	1.2896825396825398	2.3333333333333335	1.4007936507936507	2.1746031746031744	
1.4444444444444444	2.111111111111111	1.5753968253968254	2.0992063492063493	1.7380952380952381	
2.142857142857143	2.0198412698412698	2.2182539682539684	2.1468253968253967	2.4047619047619047	
2.1785714285714284	2.25	2.3253968253968256	2.0357142857142856	2.4087301587301586	1.7738095238095237
2.496031746031746	1.496031746031746	2.4365079365079363	1.2817460317460319	2.361111111111111	1.0952380952380953
2.246031746031746	0.9484126984126984	2.142857142857143	0.8055555555555556	2.111111111111111	0.6150793650793651
2.0952380952380953	0.5238095238095238	2.0238095238095237	0.5119047619047619	1.869047619047619	
0.5119047619047619	1.6507936507936507	0.5277777777777778	1.5753968253968254		
31	1.3471785642146181	2.1222004894652113	0.4032225284462031	1.955629262964085	0.5362859628334501
1.9112747848350027	0.7137038753497795	1.8427269549991483	0.9475729418485773	1.681437943620667	
1.173377557778451	1.5322456080955718	1.431439975984021	1.423375525415097	1.673373493051743	1.3870854978549387
1.8709525319903824	1.435472201268483	2.0644993456445597	1.5080522563887997	2.201595005316269	
1.6048256632158884	2.0040159663776294	1.6612768171983567	1.9153070101194647	1.7903080263011417	
1.8830492078437686	2.0322415433688636	1.931435911257313	2.217723906454117	2.052402669791174	2.358851791410288
2.294336186858896	2.451592972952915	2.0886926973513322	2.5806241820557	1.8749847572748444	2.713687616442947
1.7136957458963633	2.7741709957098775	1.576600086224654	2.7580420945720294	1.3306343438724704	
2.701590940589561	1.1209586290804447	2.6249786601847824	0.9757985188398115	2.5362697039266178	
0.8387028591681025	2.471754099375225	0.7137038753497795	2.451592972952915	0.5524148639712982	

2.4314318465306046 0.41935142958405125 2.3991740442549085 0.3749969514549689 2.2741750604365856
0.391125852592817 2.0322415433688636 0.4032225284462031 1.955629262964085
33 1.2275953850984538 1.9589276862240506 0.3522151745020727 1.7772696736368954 0.45342643154289813
1.7205913696940331 0.6113159925265859 1.663913065751171 0.7692055535102736 1.6031863115266756
0.9878218687184567 1.465539001951153 1.1173722777307133 1.3602792946286943 1.3805215460368596
1.271213388432768 1.5546049081470794 1.2145350844899057 1.7205913696940331 1.2307288856164378
1.906820082649152 1.3238432420939972 2.084951895041005 1.453393651106254 1.8865778312409869 1.5505564578654463
1.7894150244817946 1.7408336211021982 1.7853665742001614 1.9634983865920144 1.8582386792695558
2.1416301989838673 1.9715952871552804 2.2428414560246925 2.1213879475757023 2.315713561094087
1.9473045854654822 2.4736031220777748 1.7610758725103632 2.5829112796818663 1.6396223640613727
2.6233957824981964 1.485781253359318 2.5748143791186 1.263116487869502 2.522184525457371 1.076887774914383
2.4371670695430776 0.9473373659021265 2.372391865036949 0.8137385066082369 2.307616660530821
0.6760911970327143 2.250938356587959 0.5586861388653567 2.2266476548981604 0.47366868295106324
2.2104538537716283 0.3846027767551368 2.1821147018001974 0.34816672422043965 2.068758093914473
0.32792447281227455 1.8541902289879229 0.3522151745020727 1.7772696736368954
31 1.344402812878889 1.9725624058123894 0.48995588817992775 1.698781481148438 0.6787093860853097
1.610428780001238 0.9919598719708373 1.5140440151133834 1.277098134764074 1.365450835911274 1.5943646525199289
1.2811141666344013 1.7791021185549836 1.2489859116717832 1.9517914889790566 1.3373386128189833
2.10841673192182 1.4377394095771652 2.220865624290984 1.5582203656869833 2.028096094515275 1.5943646525199289
1.8835189471834928 1.7228776723704018 1.8473746603505474 1.9397433933680748 1.8513906922208747
2.1726732418470567 1.9517914889790566 2.3453626122711295 2.188737369328366 2.4176511859370207
1.9317113296274202 2.5180519826952024 1.7349257679813836 2.61042071571273 1.6144448118715653 2.614436747583057
1.4056111546145469 2.570260397009457 1.1927614654872012 2.5019878552138937 1.0923606687290193
2.421667217807348 1.0080239994521465 2.3333145166601477 0.9036071708236373 2.2650419748645843
0.7670620872325099 2.2007854649393477 0.6024047805490915 2.1525930824954203 0.4859398563096005
2.1164487956624747 0.4658596969579641 2.016047998904293 0.46184366508763686 1.88753497905382
0.47790779256894594 1.7509898954626928 0.48995588817992775 1.698781481148438
34 1.3985729941123979 1.9370040793437513 0.4759390836956811 1.715780394163422 0.5639278218579079
1.6597875607874595 0.7079093933960972 1.6317911440994781 0.8718884054257016 1.5837972869200818
1.0758622984381363 1.4598131558733076 1.271837215254005 1.3158315843351185 1.5278044535441193
1.2198438699763257 1.7637742513428183 1.1678505246986461 1.9237537752741398 1.1958469413866273
2.1437256206797066 1.3118320962368355 2.3636974660852736 1.4358162272836095 2.1597235730728386
1.5038075249544212 2.0317399539277816 1.611793703608063 2.0157420015346497 1.843764013308479
2.0077430253380832 2.071734834910612 2.071734834910612 2.291706680316179 2.2477123112350657 2.3357010493972923
2.3516990017904242 2.3676969541835566 2.175721525465971 2.4796826209354816 1.947750703863838
2.5916682876874066 1.7477762989496861 2.6116657281788216 1.5358034297406853 2.5956677757856896
1.3038331200402693 2.527676478114878 1.1838484770917783 2.4396877399526513 1.0838612746347025
2.3556984898887077 0.9398797030965131 2.27970821602133 0.7918986434600409 2.2357138469402162
0.6079221909390213 2.2157164064488013 0.49593652418709633 2.18771998976082 0.46394061940083203
2.067735346812329 0.4479426670076999 1.8877583823895925 0.4519421551059829 1.7837716918342335
0.4759390836956811 1.715780394163422
31 1.7284094680681694 2.173288377781467 0.7148022857352289 2.034774604792308 0.8507462787278184
1.947068802861605 1.0349284627822946 1.9076011919927887 1.232266517126376 1.8155100999655505
1.4734574724358092 1.648869076297215 1.6357132060076096 1.5216956634976957 1.9251423523789293
1.4515310219531334 2.166333307688362 1.4339898615669928 2.376827232322049 1.4909986328219498 2.521541805507709
1.561163274366512 2.6618710885968335 1.6400984961041447 2.4469918738666117 1.6883366871660312
2.3329743313566977 1.8418218405447615 2.275965560101741 2.131250986916081 2.3329743313566977
2.3636713620324437 2.4996153550250333 2.521541805507709 2.6837975390795092 2.6224034777280174
2.4776889045423576 2.7802739212032828 2.2978920105844165 2.8899061736166614 2.1137098265299405
2.9249884943889426 1.8681335811239723 2.8635944330374503 1.6006308852353284 2.7715033410102126
1.3945222506981765 2.666256378693369 1.232266517126376 2.5741652866661306 1.074396073651111 2.5171565154111737
0.9121403400793104 2.4864594847354278 0.6928758352525531 2.4382212936735415 0.6402523540941314
2.3549007818393735 0.6534082243837368 2.1356362770126163 0.7148022857352289 2.034774604792308

30 1.4797990567857895 2.2420614172890203 0.5695598340534744 2.108675721495688 0.7130367388150367
2.0260678066329705 0.9521649134176405 1.952155461755802 1.1565108080780475 1.804330772001465 1.352161132752905
1.656506082247128 1.6173760173121565 1.5608548124060866 1.826069696965338 1.5043336075000167 1.995633311683548
1.556507027413312 2.165196926401758 1.6260715872977058 2.299978261177771 1.7304184271242964 2.0608500865751673
1.8173741269797887 1.9217209668063795 2.0347633766185194 1.939112106777478 2.2956304761849964
2.0347633766185194 2.447802950932108 2.1695447113945328 2.521715295809276 2.347803896098292 2.5869320707008954
2.143458001437885 2.7478001154335563 1.939112106777478 2.8695380952312455 1.7608529220737188 2.860842525245696
1.5347681024494388 2.81736467531795 1.3130310678179336 2.7478001154335563 1.1912930880202444
2.6738877705563877 1.026077258294809 2.5956276406864447 0.8912959235187959 2.5521497907586985
0.7391234487716845 2.543454220773149 0.6130376839812206 2.526063080802051 0.586950974024573 2.413020670989911
0.5739076190462491 2.1999792063439547 0.5695598340534744 2.108675721495688
32 1.6217850877192983 1.8862587719298245 0.6929824561403509 1.7280701754385965 0.8289473684210527
1.6578947368421053 1.0043859649122806 1.5921052631578947 1.219298245614035 1.4956140350877194
1.4254385964912282 1.337719298245614 1.6140350877192982 1.2280701754385965 1.8640350877192982
1.1491228070175439 2.074561403508772 1.162280701754386 2.245614035087719 1.2412280701754386 2.3684210526315788
1.3070175438596492 2.530701754385965 1.3859649122807018 2.289473684210526 1.4473684210526316 2.18859649122807
1.6359649122807018 2.162280701754386 1.8596491228070176 2.241228070175439 2.0482456140350878
2.3728070175438596 2.192982456140351 2.5789473684210527 2.25 2.3728070175438596 2.4298245614035086
2.1096491228070176 2.5833333333333335 1.9429824561403508 2.6140350877192984 1.7105263157894737
2.543859649122807 1.4780701754385965 2.456140350877193 1.3333333333333333 2.3640350877192984
1.1798245614035088 2.289473684210526 1.0570175438596492 2.2280701754385963 0.9210526315789473 2.18859649122807
0.8070175438596491 2.1798245614035086 0.7017543859649122 2.1403508771929824 0.6710526315789473
2.008771929824561 0.6666666666666666 1.8421052631578947 0.6929824561403509 1.7280701754385965
32 1.2721479901705748 2.311265566198423 0.30566519862816854 2.1178231619237393 0.45413115224756473
2.039223539419353 0.6899300197607233 1.964990562609655 0.9082623044951295 1.7946913805168183
1.1571611090923524 1.6549587182867984 1.384226685216135 1.5850923871717884 1.6200255527292935
1.5457925759195954 1.8514577745477638 1.6636920096761747 1.9475239798309025 1.7466582778752489
2.0479568308087295 1.8470911288530758 1.8121579632955709 1.855824420242452 1.6462254268974221
1.9387906884415262 1.571992450087724 2.1178231619237393 1.571992450087724 2.3143222181847047
1.6549587182867984 2.5894208969500565 1.8077913176008826 2.6505539366756903 1.9780904996937194
2.676753810843819 2.0523234765034175 2.6985870393172595 1.921324105662774 2.812119827379151 1.7684915063486897
2.925652615441042 1.5632591586983478 2.99988559225074 1.3973266223001992 2.964952426693235 1.1222279435348474
2.890719449883537 0.9344621786632582 2.8033865359897745 0.7772629336544857 2.6898537479278835
0.6855633740660352 2.6461872909810022 0.5108975462785104 2.611254125423497 0.37553152974317855
2.55885437708724 0.32313178140692106 2.4103884234678437 0.32313178140692106 2.2226226585962543
0.30566519862816854 2.1178231619237393
32 1.6864102029935069 2.3397896615853875 0.6216451269370523 2.147501347600726 0.7998790444504729
2.064905141923775 1.0476676614813258 1.973614598807145 1.321539290831216 1.8127693561730824 1.4563015211462413
1.6910486320175757 1.717131644336613 1.5867165827414271 2.0040447798460215 1.5128147145041553
2.221403215837998 1.5997580889009457 2.3996371333514186 1.6910486320175757 2.616995569343395
1.8127693561730824 2.4952748451878883 1.8605882120913173 2.3735541210323814 1.9866561049666636
2.360512614872863 2.177931528639603 2.3735541210323814 2.425720145670456 2.443108820549814 2.6561200878219506
2.560482375985481 2.7039389437401855 2.651772919102111 2.7430634622187413 2.4474559892696535
2.8691313550940873 2.2300975532776772 3.0082407541289524 2.0518636357642563 3.064753947486866
1.8431995372119592 3.0343237664479896 1.5910637514612667 2.956074729490878 1.3954411590684879
2.8691313550940873 1.256331760033623 2.7604521370980994 1.0867921799598816 2.6648144252616297
0.8868224188472634 2.6213427380632344 0.7042413326140032 2.6126484006235553 0.5520904274196199
2.586565388304518 0.5260074151005827 2.47788617030853 0.5564375961394594 2.221403215837998 0.6216451269370523
2.147501347600726
35 1.5999638995780545 2.2884590643644316 0.6150201657089472 2.106112222139992 0.7123614869002914
2.048592350526925 0.8937703127568873 2.0131955064573455 1.0796037441221809 1.942401818318186
1.2698617809961716 1.8140882585659595 1.513215083974532 1.6592270657615482 1.800814442039867
1.5751595610962965 1.9777986623877655 1.530913506009322 2.1415090662095717 1.579584166604994

2.3007948645226803 1.637104038218061 2.3981361857140246 1.7255961483920101 2.4689298738531837
1.800814442039867 2.376013158170537 1.8494851026355392 2.2167273598574284 1.973374056879068 2.203453543331336
2.1238106441747817 2.2078781488400336 2.35389013062705 2.2521242039270084 2.5352989564836457 2.33619170859226
2.6591879107271748 2.5131759289401585 2.7255569933576367 2.3229178920661675 2.871568975144653
2.1459336717182693 2.977759507353392 1.9556756348442783 3.004307140405577 1.7388699649181025
2.9733349018446944 1.5397627170267167 2.920239635740325 1.3539292856614233 2.8184737090402834
1.207917303874407 2.721132387848939 1.0619053220873909 2.6370648831836876 0.9424409733525594
2.6193664611488976 0.7964289915655431 2.6016680391141076 0.6769646428307117 2.592818828096713
0.5973217436741574 2.5308743509749485 0.584047927148065 2.3981361857140246 0.5973217436741574
2.199028937822639 0.6150201657089472 2.106112222139992
34 1.6915777581501683 2.2727687233472995 0.6986832947506343 2.135350819581626 0.7947522477788465
2.0785828018831367 0.9475892185055477 2.030548325369031 1.0960594186400574 1.9825138488549248
1.2183289952214185 1.8733445840501382 1.4061001306856513 1.7336079251000112 1.6506392838483734
1.6069715779264588 1.882078125234521 1.5371032484513953 2.1047834254362856 1.5414700190435868
2.2838210197161355 1.6244386602952245 2.44539153162722 1.6899402191780966 2.62442912590707 1.7248743839156282
2.471592155180369 1.8122097957594576 2.3493225785990077 1.93884614293301 2.314388413861476 2.1484511313582004
2.3274887256380503 2.3100216432692844 2.3667896609677737 2.4934260081413258 2.44539153162722 2.59386173176173
2.7161313083430905 2.6855639141977505 2.44539153162722 2.8209338025556856 2.248886854978604 2.930103067360472
2.1309840489894345 2.999971396835536 1.9170122899720528 2.9781375438745785 1.6419057426639905
2.895168902622941 1.3798995071325026 2.7641657848571968 1.2357960775901844 2.663730061236793
1.1135265010088233 2.6287958964992613 0.969423071466505 2.6113288141304953 0.8165861007398038
2.598228502353921 0.7074168359350171 2.59386173176173 0.6943165241584428 2.44539153162722 0.6855829829740598
2.257620396162987 0.6986832947506343 2.135350819581626
30 1.2892382149775172 1.862816530006903 0.3912895545497901 1.6651544376952179 0.5478053763697062
1.6086348353713593 0.7608408005134808 1.5651582181991603 0.9825715480916951 1.4260330432481239
1.2434312511248886 1.265169559710988 1.4955956307236422 1.1956069722354699 1.695588069715757
1.1912593105182498 1.891232846990652 1.2564742362765482 2.056443992245008 1.3564704557726057
2.1607878734582853 1.4434236901170034 2.021662698507249 1.4955956307236422 1.9173188172939715 1.58689652678526
1.865146876687333 1.7912366274945948 1.8521038915356731 2.008619713355589 1.9129711555767517
2.1999168289132642 2.165135535175505 2.339042003864301 1.9173188172939715 2.491210163966997 1.7477600103223958
2.578163398311395 1.543419909613061 2.5825110600286147 1.2390835894076686 2.5086008108358766
0.9999621949605747 2.3607803124504003 0.8477940348578785 2.2433934460854634 0.7217118450585017
2.1825261820443846 0.5869343318246851 2.134701903154966 0.3869418928325702 2.095572947699987
0.37824656939813045 2.0042720516383694 0.3869418928325702 1.8434085681012333 0.38259423111535035
1.739064686887956 0.3912895545497901 1.6651544376952179
29 1.530642866519577 2.1217517039515985 0.5745392994672338 1.8990497455672688 0.7938291084241932
1.811333821984485 0.9999615288437351 1.7236178984017014 1.254337707233808 1.5613434397735513
1.4692417200116283 1.4516985352950715 1.7323894907599797 1.3815257964288445 1.9999230576874703
1.4034547773245405 2.2411418475401255 1.508713885623881 2.412187898526554 1.6183587901023608 2.201669681927873
1.6666025480728919 2.1402685354199242 1.8288770067010418 2.1183395545242285 2.1139537583450894
2.1885122933904553 2.3595583443768837 2.3069287902272135 2.4385026756013892 2.4692032488553637
2.521832803005034 2.245527643719265 2.6577924845583487 2.0613242041954187 2.7849805737533853
1.8288770067010418 2.8376101279030554 1.600815605385804 2.754280000499411 1.3464394269957312 2.662178280737488
1.2017081530841378 2.5656907647964258 0.9999615288437351 2.4911322297510594 0.8026007007824716
2.412187898526554 0.6578694268708783 2.385873121451719 0.5613819109298163 2.342015159660327 0.5526103185715379
2.127111146882507 0.5657677071089554 1.9779940767917743 0.5745392994672338 1.8990497455672688
30 0.9823028488006558 1.7552850844029884 0.26101095069344443 1.5830144671927084 0.37287278670492063
1.5457271885222164 0.5220219013868889 1.505050157245316 0.6542222530368152 1.4372551051171487
0.8372688937828673 1.349121537350531 0.9830282558584271 1.2711572274031384 1.155905638785254
1.2169211857006046 1.2914957430415888 1.193192917455746 1.41352683687229 1.2338699487326465 1.5830144671927084
1.305054753467222 1.7355533344810852 1.4101370842658816 1.5830144671927084 1.505050157245316
1.5355579307029914 1.6474197667144674 1.532168178096583 1.8677536861310116 1.5660657041606667
2.0508003268770634 1.7050455610234099 2.1185953790052308 1.5355579307029914 2.2338469676231156

1.386408816021023 2.2982522671448744 1.220310938307013 2.3253702879961415 1.0440438027737777 2.277913751506424
0.8338791411764589 2.193169936346215 0.7050685421329408 2.111815873792414 0.559309180057381 2.0440208216642466
0.440667838833088 2.0033437903873463 0.3186367450023867 1.9796155221424878 0.23728268244858586
1.9389384908655873 0.23728268244858586 1.8474151704925612 0.2542314454806277 1.6541992719272842
0.26101095069344443 1.5830144671927084
34 1.1815423377475205 1.525917474290366 0.2508416928742193 1.386408816021023 0.44744734404590475
1.2881059904351804 0.6711710160688571 1.1864134122429293 0.844048398995684 1.0779413288378614
1.0033667714968773 0.9830282558584271 1.2304801961262382 0.8881151828789928 1.3897985686274315
0.8576074094213174 1.5457271885222164 0.8745561724533593 1.688096797991368 0.9423512245815266
1.8338561600669279 0.9931975136776522 1.9287692330463622 1.0576028131994113 1.8304664074605195
1.0813310814442698 1.7016558084170015 1.1762441544237041 1.660978777140101 1.3931883212338398
1.698266055810593 1.5762349619798917 1.7863996235772106 1.7728406131515773 1.9219897278335454
1.844025417886153 2.0338515638450216 1.8880922017694617 1.89148195437587 1.9897847799617128 1.7084353136298183
2.0779183477283305 1.5762349619798917 2.1626621628885396 1.4101370842658816 2.1728314207077646
1.2270904435198298 2.128764636824456 0.999977018890469 2.0508003268770634 0.7932021098995584 1.955887253897629
0.6677812634624488 1.8880922017694617 0.5457501696317475 1.8372459126733363 0.440667838833088
1.8135176444284775 0.3254162502152035 1.8101278918220693 0.2508416928742193 1.7084353136298183
0.2508416928742193 1.5219989202773578 0.2542314454806277 1.4440346103299653 0.2508416928742193
1.386408816021023
34 1.5901640480453076 1.962074169777005 0.6212259773108001 1.7104989238283674 0.79142487520417
1.6424193646710195 1.0254483598075537 1.5743398055136717 1.2296870372795976 1.4722204667776497
1.425415769856973 1.289256651542277 1.6211445024343483 1.2296870372795976 1.8338931248010606 1.204157202595592
2.067916609404444 1.1913922852535894 2.2296055624031457 1.2637268168582716 2.370019653165176
1.3701011280416278 2.5529834684005484 1.5020052739089893 2.3657646807178416 1.5403000259349977
2.2934301491131595 1.6722041718023593 2.2551353970871513 1.9317574910897484 2.2934301491131595
2.144506113456461 2.348744790928505 2.314705011349831 2.5700033581898856 2.4338442398751896 2.4338442398751896
2.506178771479872 2.1913108103771375 2.650847834689236 2.03387682982577 2.7104174489519157 1.8806978217217374
2.718927393846584 1.6253994748816825 2.6551028071365703 1.4041409076203017 2.54447352350588 1.27223676175294
2.438099212322524 1.1488425607802468 2.3572547358231732 1.0084284700182167 2.2764102593238222
0.859504434361518 2.238115507297814 0.7318552609414906 2.2083307001664747 0.6297359222054686 2.182800865482469
0.5786762528374577 2.0849364991937813 0.5871861977321262 1.940267435984417 0.6042060875214632
1.787088427880384 0.6212259773108001 1.7104989238283674
29 1.6877153401995624 1.967353764465619 0.7372285730650582 1.8134128119071546 0.9024694601313643
1.741384732929534 1.0973689679531613 1.6651197081297004 1.2965054215971712 1.55072217092995 1.4744571461301164
1.4066660129747086 1.762569462040599 1.330400988174875 1.9744167531512478 1.2965054215971712 2.156605423506406
1.360059608930366 2.3472679855059897 1.4447985253746256 2.482850251816805 1.5125896585300331
2.2625290690617303 1.5803807916854409 2.2540551774173045 1.7837541911516637 2.2794768523505824
1.9828906447956738 2.300661581461647 2.156605423506406 2.4023482811947585 2.2582921232395172
2.4997980351056572 2.300661581461647 2.338794093861564 2.453191631061314 2.139657640217554 2.5803000057277035
1.9617059156846088 2.6226694639498334 1.699015274707404 2.5718261140832777 1.4829310377745424
2.465902468527953 1.330400988174875 2.3769266062614807 1.169397046930782 2.309135473106073 0.9956822682200498
2.2540551774173045 0.775361085464975 2.2159226650173873 0.7287546814206323 2.131183748573128
0.7160438439539933 1.9532320240401828 0.7372285730650582 1.8134128119071546
38 1.3205762046627285 1.8459557104860693 0.43951326440144506 1.7177643417023145 0.5384037488917702
1.6554999625787763 0.666595117675525 1.6188738572119894 0.7947864864592799 1.5968981939919171
0.9046648025596411 1.534633814868379 1.0475066134901108 1.4357433303780538 1.1976736454939378 1.33319023535105
1.4027798355479455 1.2562754140807972 1.5456216464784152 1.1903484244205804 1.6701504047254911
1.1720353717371867 1.8056669945826034 1.2159866981773313 1.9521714160497519 1.2855762983742267
2.0803627848335067 1.358828509107801 2.186578490397189 1.44673116198809 2.040074068930041 1.508995541111628
1.9924601319532176 1.6261990782853468 1.9741470792698241 1.7543904470691016 1.9558340265864305
1.9045574790729285 1.9924601319532176 2.047399290003398 2.065712342686792 2.2048915430805827
2.2085541536172615 2.24151764844737 2.0217610162466473 2.3587211856210883 1.875256594779499 2.4722621122581283
1.7214269522389931 2.527201270308309 1.4796946568181983 2.486912554404843 1.2892389089109055

2.4356360068913414 1.120758824223685 2.351395964547731 0.9962300659766088 2.274481143277478 0.8643760866561753
2.2085541536172615 0.7654856021658502 2.179253269323832 0.6519446755288102 2.1572776061037593
0.5310785278184128 2.153614995567081 0.4724767592315534 2.1206515007369724 0.4468384854748025
2.0180984057099685 0.4175376011813728 1.8422930999493905 0.4248628222547302 1.761715668142459
0.43951326440144506 1.7177643417023145
34 1.5084724368943148 2.163103562257776 0.57543351667798 1.9589226099675918 0.6774607359471255
1.8936251896353387 0.8366231980069923 1.860976479469212 1.0365965477745174 1.8038412366784908
1.2284077200005106 1.6569220409309213 1.4447054248510989 1.530408289037181 1.6569220409309213
1.4569486911633964 1.8895441008645728 1.4079756259142067 2.040544385382908 1.4324621585388015
2.2527610014627304 1.554894821661776 2.4527343512302555 1.6936518398678138 2.260923179004262
1.7548681714293008 2.134409427110522 1.8977062784061045 2.105841805715161 2.0976796281736294
2.1180850720274584 2.2445988239211987 2.1711392260474143 2.436409996147192 2.313977333024218
2.5588426592701667 2.4976263277086796 2.599653546977825 2.265004267775028 2.7139240325592677
2.1017607169443955 2.8241134293699446 1.9221928110306994 2.8771675833899004 1.6528409521601555
2.8241134293699446 1.4079756259142067 2.754734920266926 1.3018673178742954 2.660869878539312 1.15086703335596
2.5833291918947614 1.0243532814622198 2.5139506827917426 0.8529475530900555 2.460896528771787
0.6611363808640622 2.416004552293363 0.5631902503656826 2.3792747533564707 0.555028072824151
2.2568420902334965 0.5591091615949169 2.093598539402864 0.5591091615949169 2.020138941529079 0.57543351667798
1.9589226099675918
36 1.5447750833453782 2.2177029003171893 0.580907862249601 2.0746709366057177 0.6680440415870411
2.008281466634335 0.800822981529807 1.9792360735218548 0.9750953402046874 1.904547919804049 1.1286209895135104
1.7966650311005516 1.3443867669205052 1.6472887236649398 1.5601525443274997 1.551853860581077
1.829859766086243 1.5020617581025397 2.0124308085075464 1.5726005699471342 2.1535084321967353
1.6348406980453056 2.2738393465198667 1.6638860911577857 2.4356636795751125 1.7551716123684373
2.2530926371538094 1.771768979861283 2.1535084321967353 1.8589051591987231 2.1576577740699463
2.0124308085075464 2.174255141562792 2.2323459277877524 2.211599218421695 2.4066182864626326 2.331930132744827
2.51450117516613 2.4356636795751125 2.57259196139109 2.323631448998404 2.6265334057428387 2.1576577740699463
2.726117610699913 2.0248788341271804 2.8132537900373533 1.8713531848183573 2.875493918135525
1.7095288517631115 2.8630458925158906 1.514509783722174 2.8049551062909304 1.3111920319348136
2.7219682688267017 1.1410690151331448 2.6182347219964157 1.0165887589368017 2.57259196139109
0.8589137677547671 2.5103518332929187 0.7427321953048469 2.4979038076732842 0.6182519391085038
2.48545578205365 0.580907862249601 2.3900209189697867 0.580907862249601 2.290436714012712 0.5892065459960238
2.1452097484503123 0.580907862249601 2.0746709366057177
38 1.2280766283524904 1.7757547892720307 0.40229885057471265 1.6590038314176245 0.46360153256704983
1.5977011494252873 0.6053639846743295 1.578544061302682 0.7164750957854407 1.5555555555555556
0.8352490421455939 1.4980842911877394 0.9310344827586207 1.4137931034482758 1.0804597701149425
1.3295019157088122 1.206896551724138 1.2911877394636015 1.3409961685823755 1.260536398467433
1.5057471264367817 1.2452107279693487 1.6513409961685823 1.2490421455938698 1.7969348659003832
1.3026819923371646 1.9080459770114941 1.3639846743295019 1.996168582375479 1.4521072796934866
1.9003831417624522 1.5057471264367817 1.793103448275862 1.5632183908045978 1.739463601532567
1.6666666666666667 1.7241379310344827 1.8314176245210727 1.7547892720306513 1.9540229885057472
1.8199233716475096 2.0613026819923372 1.9080459770114941 2.103448275862069 1.9770114942528736
2.1302681992337167 1.8620689655172413 2.2222222222222223 1.7088122605363985 2.310344827586207
1.5172413793103448 2.3333333333333335 1.3256704980842913 2.314176245210728 1.160919540229885
2.2835249042145596 0.9846743295019157 2.206896551724138 0.89272030651341 2.1455938697318007 0.7854406130268199
2.057471264367816 0.6819923371647509 2.0114942528735633 0.5517241379310345 1.9846743295019158
0.46360153256704983 1.9808429118773947 0.4099616858237548 1.9195402298850575 0.4061302681992337
1.8314176245210727 0.40229885057471265 1.7126436781609196 0.40229885057471265 1.6590038314176245
37 1.2304155671596406 1.6190752899871903 0.38619771241093 1.5068607171747894 0.5275736607042169
1.462034196984235 0.6861905783015632 1.4034149013504331 0.8206701388732263 1.3172100548301362
0.9241159546975825 1.2379015960314632 1.0792846784341168 1.1654895249544137 1.241349789892275
1.1241111986246712 1.3896221259071857 1.1068702293206119 1.5516872373653436 1.0965256477381762
1.71030415496269 1.1516967495111663 1.8275427462302938 1.2241088205882156 1.9206439804722144 1.293072697804453

1.793060807622175 1.293072697804453 1.7172005426843138 1.348243799577443 1.6999595733802544 1.458586003123423
1.6930631856586307 1.5896173698342744 1.7172005426843138 1.6999595733802544 1.7447860935708088
1.8103017769262344 1.789612613761363 1.8585764909776006 1.8585764909776006 1.8689210725600363
1.9137475927505907 1.8861620418640956 1.8413355216735412 1.9551259190803332 1.7240969304059375
2.0551235410438773 1.5551354312261556 2.1206392243993033 1.3861739320463737 2.1137428366776794
1.1965232697017205 2.0861572857911845 1.024113576661127 2.04133076560063 0.8758412406462163 1.9689186945235806
0.7758436186826718 1.9171957866114024 0.66205322127588 1.872369266420848 0.5448146300082762 1.8413355216735412
0.4068868755758012 1.82064635850867 0.3620603553852469 1.7413378997099969 0.3620603553852469
1.6516848593288882 0.3551639676636231 1.5792727882518387 0.38619771241093 1.5068607171747894
33 1.2731254355400696 1.68798606271777 0.4738675958188153 1.6062717770034842 0.5679442508710801
1.5679442508710801 0.6655052264808362 1.5261324041811846 0.8153310104529616 1.4599303135888502
0.926829268292683 1.3379790940766552 1.1010452961672474 1.2369337979094077 1.2752613240418118
1.1951219512195121 1.4634146341463414 1.1742160278745644 1.6620209059233448 1.1846689895470384
1.7944250871080138 1.2473867595818815 1.9477351916376306 1.3379790940766552 1.818815331010453
1.3205574912891986 1.7560975609756098 1.4320557491289199 1.7560975609756098 1.5609756097560976
1.7700348432055748 1.6794425087108014 1.8083623693379791 1.7735191637630663 1.8571428571428572
1.8397212543554007 1.9442508710801394 1.8815331010452963 1.9094076655052266 1.9581881533101044
1.8222996515679442 2.0696864111498257 1.6655052264808363 2.156794425087108 1.5365853658536586
2.1742160278745644 1.32404181184669 2.153310104529617 1.1533101045296168 2.1045296167247387 0.9895470383275261
2.0418118466898956 0.9128919860627178 2.010452961672474 0.759581881533101 1.9581881533101044
0.6167247386759582 1.9268292682926829 0.4912891986062718 1.9128919860627178 0.445993031358885
1.8222996515679442 0.4355400696864111 1.67595818815331 0.4738675958188153 1.6062717770034842
34 0.9713995748913622 1.785495907561026 0.16148819152548652 1.693908051533295 0.2542580036784256
1.6629847808156486 0.3916947624235205 1.62175375319212 0.5669266298235165 1.5221121031019262
0.7387225782548852 1.3778035064195766 0.9517495543097823 1.2850336942666374 1.1819561252078163
1.2506745045803638 1.3468802357019303 1.2472385856117363 1.5083684272274167 1.3090851270470292
1.6148819152548652 1.3606239115764396 1.7076517274078042 1.408726777137223 1.583958644537219 1.419034534043105
1.49118883238428 1.4946247513529072 1.4637014806352608 1.6526770239097663 1.4877529134156524 1.786677863686234
1.5221121031019262 1.9309864603685836 1.5873945635058462 1.989397082835249 1.7007798894705497
2.0203203535528953 1.6354974290666295 2.1337056795175986 1.5186761841332987 2.2333473296077924
1.3400083977646755 2.312373465886222 1.1475969355215425 2.284886114137203 0.9276981215293907
2.2436550865136744 0.7799536058784137 2.1886803830156367 0.6390809281646913 2.1165260846744616
0.515387845294106 2.0684232191136784 0.377951086549011 2.0306281104587773 0.27487351749018984
2.0203203535528953 0.20615513811764238 2.0065766776783858 0.1649241104941139 1.9241146224313288
0.1511804346196044 1.796985620592116 0.14430859668234966 1.742010917094078 0.16148819152548652
1.693908051533295
35 1.2833854101757867 1.6943807523434378 0.4913828230186955 1.5944169921725504 0.5979063720647064
1.5566183134788047 0.7319198692516233 1.5119471477498323 0.821262200709568 1.481020956091313
0.9484032108612584 1.3744974070453022 1.130524117294761 1.278282588552131 1.3298262413163298
1.2164302052350924 1.5153833912674457 1.1889402570941863 1.680323080112882 1.2095577181998658
1.7937191161941193 1.2679738579992912 1.883061447652064 1.3332624848339432 1.9620950485571689
1.3813698940805286 1.845262768958318 1.3882423811157552 1.7627929245356 1.4363497903623408 1.7387392199123073
1.5737995310668709 1.7559204375003734 1.6940680541833348 1.776537898606053 1.7937191161941193
1.8383902819230915 1.8761889606168374 1.9517863180043291 1.924296369863423 1.8933701782049037
2.0102024578037545 1.7937191161941193 2.1064172762969253 1.6768868365952687 2.178578390166804
1.560054556996418 2.2026320947900966 1.4054235987038215 2.1682696596139643 1.2267389357879321
2.1304709809202182 1.0514905163896562 2.0686185976031797 0.8968595580970596 1.9964574837333013
0.7594098173925294 1.9311688568986496 0.632268807240839 1.91055139579297 0.5051277970891486 1.8933701782049037
0.46732911839540275 1.8383902819230915 0.46389287487778946 1.7456117069475336 0.46389287487778946
1.6528331319719758 0.4913828230186955 1.5944169921725504
33 1.2150842869776493 1.8740342367342149 0.4346262618780033 1.7879747033355256 0.5052971987687355
1.7385050475120132 0.6395719788611267 1.72083731328933 0.8021151337098109 1.6749012043103542
0.9717253822475683 1.565361252129719 1.1236678965626425 1.4770225810163038 1.2968116919449366

1.420485831503718 1.5052909557725966 1.3816168162138152 1.6430992827095245 1.4134187378146448
1.7455721412010863 1.4734890341717672 1.830377265469965 1.5370928773734263 1.893981108671624
1.5865625331969389 1.8162430780918186 1.6254315484868416 1.7385050475120132 1.703169579066647
1.7314379538229399 1.8056424375582087 1.7314379538229399 1.9540514050287463 1.766773422268306
2.102460372499284 1.8727798276044043 2.1519300283227967 1.9469843113396732 2.1660642157009433
1.8162430780918186 2.2614699805034317 1.646632829554061 2.3639428389949932 1.4275529251927912
2.395744760595823 1.1236678965626425 2.3427415579277735 0.929322820113129 2.233201605747139 0.7879809463316645
2.1589971220118698 0.6572397130838098 2.120128106721967 0.5512333077477115 2.102460372499284
0.45582754294522293 2.084792638276601 0.4240256213443934 2.0070546076967957 0.42755916818893
1.9010482023606972 0.4240256213443934 1.8374443591590381 0.4346262618780033 1.7879747033355256
36 1.1942088683497374 1.7848799955908525 0.44063746121599967 1.7083175419451064 0.4982592830673227
1.6744223526207986 0.5863867753105226 1.6473062011613526 0.6711247486212918 1.626969087566768
0.813484543783384 1.5727367846478757 0.9117805928238762 1.4710512166749528 1.043971831188676
1.3829237244317527 1.2405639292696606 1.3015752700534144 1.4337665084182143 1.2575115239318144
1.6235795686343373 1.2812381564588298 1.742212731269414 1.3320809404452914 1.8371192613774756
1.3727551676344605 1.9116886778909523 1.4337665084182143 1.7625498448639987 1.4710512166749528
1.7015385040802449 1.5829053414451681 1.6947594662153833 1.7184860987423987 1.7218756176748296
1.8472878181747678 1.772718401661291 1.9557524240125523 1.8506773371071985 1.9998161701341524
1.9150781968233832 2.0404903973233215 1.8269507045801832 2.1286178895665215 1.6913699472829526
2.2303034575394447 1.5862948603775988 2.2743672036610443 1.454103622012799 2.2743672036610443
1.2846276753912607 2.250640571134029 1.0948146151751377 2.20996634394486 0.9083910738914455 2.125228370634091
0.7694207976617841 2.0540484730530446 0.6168924457023995 2.0269323215935984 0.47453265054030735
2.0099847269314446 0.4338584233511381 1.962531461877414 0.4270793854862766 1.8981306021612294
0.4338584233511381 1.8133926288504603 0.4270793854862766 1.7489917691342756 0.44063746121599967
1.7083175419451064
34 1.0391669891048794 1.6555260577381674 0.3435195312409962 1.5475384823232998 0.3775313660173325
1.5135266475469635 0.47616568686870764 1.482915996248261 0.6292189433622208 1.4318982440837564
0.7414579981241305 1.3298627397547478 0.9659361076479498 1.1972165841270364 1.1666059328283338
1.1393964650072648 1.336665106710015 1.1257917310967303 1.482915996248261 1.1427976484848983
1.6087597849207051 1.2108213180375709 1.7312023901155156 1.3060544554113123 1.5373349318903988
1.3298627397547478 1.4761136292929937 1.4352994275613902 1.4591077119048255 1.5815503170996361
1.4897183632035282 1.7141964727273475 1.527131381457498 1.8162319770563562 1.591753867532537 1.863848545743227
1.727801206637882 1.9046627474748306 1.6325680692641404 1.999895884848572 1.5135266475469635
2.0815242883117793 1.3876828588745194 2.118937306565749 1.2312284189033726 2.1121349396104816
1.0713727954545922 2.0849254717894126 0.9149183554834454 2.037308903102542 0.7822721998557339
1.9692852335498694 0.6802366955267252 1.9046627474748306 0.5782011911977164 1.880854463131395
0.445555035570005 1.863848545743227 0.363926632106798 1.8400402613997917 0.3299147973304617 1.7652142248918519
0.3299147973304617 1.6563763536075757 0.32311243037519444 1.5883526840549034 0.3435195312409962
1.5475384823232998
36 1.1336397381311234 1.4879418486190032 0.34456313482417306 1.4323016584847976 0.3884780441645088
1.3985209589922318 0.48982014264220675 1.361362189550409 0.6215648706632141 1.3343376299563563
0.756687668633478 1.2498858812249414 0.9120788862992816 1.1417876428487304 1.0472016842695455
1.0674701039650851 1.2127271117831189 1.0134209847769795 1.3681183294489223 0.9965306350306965
1.5268876170639825 1.010042914827723 1.665388484983503 1.0640920340158284 1.749840233714918 1.1113850133054208
1.8275358425478196 1.1451657127979868 1.8782068917866688 1.1755683423412961 1.732949883968635
1.2059709718846057 1.6518762051864766 1.2600200910727113 1.6248516455924238 1.3681183294489223
1.6113393657953974 1.4863507776729032 1.665388484983503 1.6282297155416805 1.7261937440701218
1.716059534222352 1.8646946119896424 1.7971332130045103 1.7464621637656614 1.908609521329978
1.5572902466072918 2.026841969553959 1.3681183294489223 2.0403542493509854 1.16205606254427 2.009951619807676
0.9762622153351569 1.942390220822544 0.8276271375678667 1.851182332192616 0.7161508292423988
1.7937551430552539 0.6046745209169311 1.766730583461201 0.49319821259146335 1.7464621637656614
0.4087464638600484 1.7363279539178915 0.34794120477342966 1.699169184476069 0.33780699492565985
1.614717435744654 0.33105085502714665 1.48972884762216 0.34456313482417306 1.4323016584847976

36 0.9358282828282828 1.7650033670033671 0.2727272727272727 1.7003367003367003 0.3400673400673401
1.6498316498316499 0.43097643097643096 1.6195286195286196 0.5218855218855218 1.5622895622895623
0.6363636363636364 1.4612794612794613 0.797979797979798 1.3602693602693603 0.9797979797979798
1.3063973063973064 1.1582491582491583 1.2693602693602695 1.3198653198653199 1.2727272727272727
1.4242424242424243 1.3232323232323233 1.5353535353535352 1.404040404040404 1.5824915824915824
1.4444444444444444 1.4848484848484849 1.4444444444444444 1.3905723905723906 1.4545454545454546
1.2794612794612794 1.5791245791245792 1.2491582491582491 1.7205387205387206 1.2727272727272727
1.797979797979798 1.32996632996633 1.936026936026936 1.4579124579124578 1.9898989898989898 1.5622895622895623
2.0202020202020203 1.5084175084175084 2.1313131313131315 1.3771043771043772 2.2222222222222223
1.2188552188552189 2.265993265993266 1.0740740740740742 2.249158249158249 0.8888888888888888 2.218855218855219
0.7441077441077442 2.1717171717171717 0.622895622895623 2.0841750841750843 0.5420875420875421
2.0336700336700337 0.4377104377104377 1.9966329966329965 0.3367003367003367 1.9865319865319866
0.26936026936026936 1.9461279461279462 0.25925925925925924 1.8754208754208754 0.25252525252525254
1.7811447811447811 0.25925925925925924 1.7306397306397305 0.2727272727272727 1.7003367003367003
32 1.1931696710660533 1.7835199569780307 0.43536407610163863 1.6836345130493056 0.4999884311479756
1.636016567225689 0.6258358593961055 1.6122075943138805 0.8027025153123962 1.5407806755784554
0.9319512254050701 1.4523473476203101 1.0577986536532 1.3911242744185173 1.2346653095694908 1.3435063285949005
1.4081306836412375 1.312894791994004 1.5339781118893674 1.2958883827712837 1.6700293856711295
1.319697355683092 1.7788704046965391 1.3639140196621649 1.9863485972137263 1.499965293443927
1.8162845049865237 1.5271755482002793 1.7448575862510987 1.6326152853811449 1.7176473314947462
1.7958768139192594 1.7516601499401867 1.9387306513901095 1.8026793776083474 2.0543742341046074
1.962539624301918 2.1189985891509444 1.8298896323646998 2.234642171865442 1.642819130914777 2.3196742179790433
1.4081306836412375 2.309470372445411 1.1972512092795062 2.2720562721554267 1.0033781441404952
2.1938267897309136 0.8673268703587332 2.1189985891509444 0.7312755965769712 2.0271639793482548
0.6088294501733853 1.9999537245919023 0.4727781763916232 1.9965524427473584 0.43536407610163863
1.9455332150791975 0.4149563850343743 1.8366921960537879 0.4217589487234624 1.7312524588729223
0.43536407610163863 1.6836345130493056
33 1.0065806458101767 1.5923895241417874 0.2457280638050171 1.4334137055292666 0.3208116388565501
1.3992848077785698 0.43002411165877996 1.375394579353082 0.5426494742360795 1.3446785713774547
0.6689263959136577 1.266182106550852 0.8361579948920721 1.170621192848901 1.0272798222959743
1.1126020666727163 1.2252274292500158 1.0716473893718803 1.3992848077785698 1.064821609821741
1.5119101703558693 1.1023633973475073 1.6347742022583778 1.1945114212743888 1.7508124546107469
1.3037238940766187 1.600645304507681 1.320788342951967 1.4982586112555905 1.4026976975536394 1.460716823729824
1.5767550760821931 1.4777812726051724 1.7439866750606077 1.580167965857263 1.890740935388604
1.7303351159603289 1.921456943364231 1.6620773204589352 2.0067791877409733 1.5119101703558693
2.0989272116678546 1.3378527918273153 2.136468999193621 1.191098531499319 2.115991660543203 1.040931381396253
2.0852756525675757 0.8634611130926296 2.0340823059415305 0.6791650652388668 1.921456943364231
0.5528881435612886 1.836134698987489 0.42661122188371026 1.7883542421365135 0.3071600797562714
1.771289793261165 0.24231517402994743 1.7405737852855379 0.21842494560445966 1.6484257613586566
0.22866361492966872 1.4846070521553119 0.2457280638050171 1.4334137055292666
32 1.0888253352215325 1.7785924379711933 0.24065584420258443 1.7320441744721218 0.30844622285119977
1.6778118715532295 0.41352130975655355 1.6506957200937833 0.5253754345267688 1.6066319739721835
0.6779037864861533 1.5015568870668297 0.8406006952428301 1.365976129769599 1.0982041341075683
1.2541220049993838 1.2710695996615375 1.233784891404799 1.454103622012799 1.233784891404799 1.643916682228922
1.2744591185939682 1.7489917691342756 1.3151333457831376 1.8676249317693525 1.379534205499322
1.7388232123369833 1.3998713190939067 1.6032424550397526 1.5117254438641219 1.5659577467830141
1.6812013904856602 1.6303586064991988 1.8405087803099063 1.711707060877537 1.9930371322692908
1.860845893904491 2.050658954120614 1.8066135909855987 2.16590259782326 1.6540852390262142 2.2777567225934754
1.494777849201968 2.348936620174521 1.308354307918276 2.34215758230966 1.0575299069183992 2.2845357604583367
0.8439902141752609 2.1964082682151367 0.6745142675537226 2.125228370634091 0.5287649534591996
2.0709960677151984 0.34912045004036896 2.07777510558006 0.27116151459446136 2.0404903973233215
0.2440453631350152 1.9150781968233832 0.23048728740529215 1.8100031099180294 0.24065584420258443
1.7320441744721218

35 0.974895400973587 1.6340809866772334 0.1811917315822805 1.5770391452531822 0.2583659876265852
1.5300635111392575 0.39593748753165 1.483087877025333 0.563707609367095 1.4025582185443195 0.7079899141455775
1.2884745356962168 0.8623384262341869 1.2113002796519123 1.0468855602531764 1.1643246455379876
1.224721889398748 1.1307706211708988 1.3958474136709016 1.1676800479746967 1.513286498955713 1.21801108452533
1.5938161574367267 1.2683421210759636 1.6508579988607779 1.3086069503164703 1.5703283403797643
1.3253839625000148 1.4763770721519152 1.3455163771202683 1.415979828291155 1.4965094867721687
1.385781206360775 1.6307255842405246 1.4226906331645728 1.7246768524683738 1.4629554624050796
1.8320497304430585 1.5636175355063466 1.8689591572468562 1.6709904134810314 1.915934791360781
1.5736837428164734 1.9998198522785033 1.382425803924066 2.113903535126606 1.2213664869620389 2.147457559493695
1.0334639505063408 2.1239697424367328 0.8388506091772245 2.076994108322808 0.6744358897784886
1.9863982425316677 0.5804846215506394 1.909223986487363 0.47311174357595465 1.8924469743038186
0.3154078290506364 1.8689591572468562 0.21139035351266058 1.8555375475000206 0.164414719398736
1.7716524865822982 0.1711255242721538 1.677701218354449 0.1677701218354449 1.610593169620271
0.1811917315822805 1.5770391452531822
29 1.1668740768166075 1.677314771968097 0.41210337238923267 1.5909892491420377 0.537085542704
1.5470765947071194 0.6823350919887295 1.4896523542922264 0.8444741237484277 1.378181769957434
1.0437700169530566 1.2565774961376603 1.2396880136626918 1.2059090487127546 1.4052049419173835
1.1991532557227673 1.563966077182088 1.2126648417027421 1.715971419456805 1.259955392632654 1.8443314862665658
1.3714259769674464 1.7463724879117484 1.378181769957434 1.6484134895569307 1.4727628718172578
1.6484134895569307 1.6078787316170062 1.6619250755369055 1.7497503844067421 1.7092156264668175
1.878110451216503 1.878110451216503 1.9355346916313962 1.7328609019317736 2.0706505514311444
1.5335650087271446 2.171987446280956 1.3545364944924778 2.171987446280956 1.1484848082978616
2.1517200673109937 0.9627005010732075 2.087540033906113 0.8174509517884779 2.006470518026264
0.6620677130187672 1.9355346916313962 0.5303297497140126 1.915267312661434 0.40534757939924526
1.8814883477114968 0.3817023039342893 1.7227272124467923 0.39521388991426415 1.6382798000719496
0.41210337238923267 1.5909892491420377
32 1.0143744883439063 1.8335188000928284 0.3435294662989492 1.7346537407174665 0.46597561270253507
1.6938383585829377 0.5986256046397531 1.5815960577129842 0.785696106089676 1.4319396565530458
0.9829704530732309 1.3537101741285327 1.1598371089895216 1.3230986375276361 1.3367037649058124
1.306092228304916 1.4897614479102947 1.3537101741285327 1.6156088761584246 1.4625511931539423
1.646220412759321 1.5033665752884708 1.4591499113093982 1.510169138977559 1.3435063285949005
1.6054050306247925 1.2720794098594754 1.772067841007451 1.3264999193721803 1.9013165511001249
1.4047294017966934 2.0067562882809904 1.5169717026666472 2.047571670415519 1.6768319493602175
2.0577755159491513 1.6224114398475127 2.166616534974561 1.4829588842212065 2.2890626813781467
1.2618755643258432 2.3332793453572194 1.0850089084095524 2.3026678087563233 0.8979384069596297
2.2618524266217945 0.7414794421106032 2.2074319171090897 0.653046114152458 2.1428075620627527
0.5510076588161363 2.0679793614827835 0.41155510318983024 2.044170388570975 0.3367269026098611
2.0271639793482548 0.31291792969805277 1.9217242421673892 0.3163192115425968 1.8230870686756118
0.3333256207653171 1.772067841007451 0.3435294662989492 1.7346537407174665
35 1.1079085571674259 1.700568394167502 0.32312738253477746 1.5986302083299517 0.3605421320914359
1.5374060726917833 0.45237833554868845 1.5101953457414863 0.5578199024810895 1.4863859596599762
0.6938735372325747 1.425161824021808 0.8469338763279957 1.323121597958194 1.0544156693240108
1.2516934397136643 1.2244827127633673 1.2074760084194316 1.3707403701212137 1.1972719858130703
1.5135966866102732 1.2346867353697286 1.6224395944114616 1.3027135527454712 1.68366373004963 1.374141710990001
1.7482892065565854 1.425161824021808 1.65985434396812 1.4387671874969565 1.5680181405108675 1.4829846187911893
1.5203993683478476 1.5850248448548032 1.4999913231351247 1.7210784796062883 1.530603390954209
1.8299213874074765 1.581623503986016 1.9285602726023034 1.6700583665744815 1.9863830673716847
1.765295910900521 2.020396476059556 1.6190382535426744 2.1156340203855954 1.452372550972105 2.1972662012364865
1.3027135527454712 2.221075587317997 1.1632585771251989 2.1870621786301254 1.010198238029778 2.153048769942254
0.8605392398031442 2.09862731604166 0.7482949911331689 2.0340018395347044 0.6292480607256192
1.9557709995526005 0.5238064937932182 1.918356249995942 0.3673448138290102 1.894546863914432
0.3163247007972032 1.789105296982031 0.31972604166599033 1.687065070918417 0.32312738253477746
1.5986302083299517

31 1.0785566767504524 1.6416456886784092 0.31291792969805277 1.6088063124693366 0.3673384392107576
1.5645896484902637 0.47617945823616725 1.5271755482002793 0.6054281683288412 1.455748629464854
0.7516832876442354 1.3503088922839885 0.9217473798714381 1.2414678732585789 1.0646012173422883
1.1904486455904182 1.2380665914140347 1.1598371089895216 1.3945255562630612 1.1496332634558895
1.5407806755784554 1.18364608190133 1.707443485961114 1.2618755643258432 1.8060806594528915 1.312894791994004
1.584997339557528 1.3673153015067088 1.5271755482002793 1.4863601660657506 1.5271755482002793
1.6054050306247925 1.5611883666457198 1.7312524588729223 1.6224114398475127 1.8230870686756118
1.768666559162907 1.8877114237219488 1.6904370767383938 2.023762697503711 1.5169717026666472
2.1258011528400322 1.3537101741285327 2.156412689440929 1.1768435182122419 2.1428075620627527
0.9217473798714381 2.0781832070164157 0.7618871331778676 1.9965524427473584 0.6190332957070174
1.9319280877010214 0.49658714930343156 1.8945139874110368 0.3401281844544052 1.8843101418774046
0.3095166478535087 1.7958768139192594 0.31291792969805277 1.6700293856711295 0.31291792969805277
1.6088063124693366
36 1.0712307898356184 1.5673821500649718 0.23467215870241281 1.510064325563352 0.29589098271173786
1.4624496846672101 0.39792235606061305 1.4352413184408435 0.523761049857559 1.3978298148795893
0.6700060183242801 1.3094026246438975 0.7856415747863386 1.2073712512950223 0.9658970010360179
1.1189440610593306 1.1461524272856973 1.0781315117197805 1.319605761978785 1.0509231454934138
1.4964601424501687 1.0577252370500057 1.612095698912227 1.1189440610593306 1.7277312553742856
1.197168113960135 1.7889500793836106 1.2447827548562767 1.6801166144781439 1.261787983747756
1.5610800122377895 1.3060015788656016 1.5270695544548312 1.4012308606578852 1.486257005115281
1.5202674628982396 1.489658050893577 1.595090470020748 1.530470600233127 1.7073249807045106 1.5814862869075645
1.7787469420487232 1.6869187060347355 1.8399657660580482 1.782147987827019 1.8535699491712316
1.6597103398083688 1.989611780303065 1.4726528220020978 2.0882421078736444 1.2855953041958268
2.0916431536519404 1.091735694832964 2.071236878982165 0.9182823601398762 2.030424329642615 0.7516311170033801
1.9590023682984026 0.5917819654234757 1.8807783153975983 0.4353338596218672 1.843366811836344
0.2788857538202587 1.843366811836344 0.22106797558922944 1.7753458962704274 0.20406274669775026
1.676715568699848 0.22106797558922944 1.5882883784641562 0.23467215870241281 1.510064325563352
31 1.1120713569479561 1.5918695077707765 0.2754958616883237 1.4761136292929937 0.3945372834055006
1.4489041614719247 0.5441893564213801 1.4046887762626876 0.7210508972583287 1.2890485380231442
0.8741041537518419 1.1836118502165018 1.0679716119769584 1.108785813708562 1.2754438041126097
1.064570428499325 1.4489041614719247 1.0611692450216912 1.5815503170996361 1.1053846302309285
1.7312023901155156 1.1972165841270364 1.8332378944445245 1.2516355197691744 1.6971905553391793
1.2754438041126097 1.5883526840549034 1.3672757580087176 1.544137298845666 1.5067242805916963
1.5645443997114679 1.6155621518759724 1.6053586014430714 1.7380047570707828 1.6801846379510113
1.8298367109668907 1.8298367109668907 1.8944591970419296 1.6869870049062785 2.0475124535354428
1.4795148127706272 2.152949141342085 1.3230603727994805 2.156350324819719 1.081576345887493 2.098530205699947
0.89111007114001 2.0407100865801757 0.7244520807359623 1.9488781326840676 0.5782011911977164
1.8876568300866623 0.3979384668831342 1.8536449953103262 0.32311243037519444 1.836639077922158
0.25168757734488834 1.755010674458951 0.25508876082252196 1.5679455831891016 0.2754958616883237
1.4761136292929937
35 1.0690161413027652 1.4448610158341848 0.30134159059344656 1.3663101664407407 0.3732526519850645
1.3012477775626101 0.4931044209710944 1.280701760022148 0.5958345086734057 1.2635800787384295
0.7362322951998979 1.1574256547793744 0.87663008172639 1.0375738857933445 1.0273008770231133
0.9827845056854451 1.1950933536035553 0.9553898156314954 1.3491884851570222 0.9314194618342895
1.4998592804537454 0.9519654793747517 1.612862376926288 1.0101791957393949 1.7087437921151118
1.0820902571310127 1.7840791897634736 1.160849991036118 1.6573787482639561 1.1711229998063493
1.5443756517914138 1.222488043657505 1.4553429091160772 1.3457641489002785 1.4484942366025897
1.4621915816295648 1.482737599170027 1.5991650318993131 1.564921669331876 1.7155924646285994
1.6676517570341873 1.7635331722230112 1.7601088359662675 1.7977765347904484 1.636832730723494
1.9279013125467093 1.4279482190621275 2.003236710195071 1.2293367161709923 1.9963880376815837
1.0273008770231133 1.9621446751141465 0.8321137103887218 1.8799606049522974 0.6677455700650237
1.7601088359662675 0.6129561899571243 1.7053194558583682 0.4965287572278381 1.6916221108313934
0.3698283157283208 1.6779247658044185 0.2773712367962406 1.612862376926288 0.284219909309728

1.4964349441970017 0.2807955730529843 1.4074022015216652 0.30134159059344656 1.3663101664407407
30 1.0560481698067448 1.5134285927937654 0.25423582743408746 1.420330822598435 0.3016931818884504
1.3694836571116176 0.46101430041381186 1.3389753578195271 0.6406742851339003 1.271179137170437
0.8033852146917163 1.1660949951643478 0.9796553883793503 1.0779599083205307 1.1762644282617112
1.0406719869635312 1.3762632791765266 1.0305025538661676 1.5389742087343425 1.0949089634828033
1.6813462720974315 1.1762644282617112 1.752532303778976 1.2237217827160742 1.644058350740432
1.2508402709757103 1.5796519411237966 1.3254161136897091 1.5559232638966152 1.45422893292298
1.5559232638966152 1.5864315631887056 1.5932111852536146 1.7288036265517945 1.7491424927465216
1.806769280298248 1.596600996286069 1.901683989206974 1.4372798777607076 1.9864292650183364 1.2372810268458923
2.010157942245518 1.0610108531582583 1.989819076050791 0.9084693566978057 1.9321922884990645
0.7627074823022623 1.8711756899148835 0.6271150410040823 1.772871169973703 0.49491241073835684
1.7254138155193401 0.29491355982354145 1.6982953272597041 0.25423582743408746 1.6203296735132506
0.25084601640163295 1.5050760984097975 0.25423582743408746 1.420330822598435
34 1.1125790921383587 1.7487054988015371 0.2534231714297454 1.6609491641003584 0.2979434583025385
1.613004239775812 0.44520286872793113 1.5753332278065255 0.5856130042498171 1.5239636660302258
0.7842419764515094 1.3801288930565865 0.9554738490391752 1.2910883193110003 1.126705721626841
1.232869482631194 1.33218396873204 1.2054723830171674 1.506840478771459 1.2088970204689207 1.6814969888108784
1.2842390444074936 1.7945100247187378 1.3493071559908065 1.8698520486573107 1.4041013552188597
1.787660749815231 1.4349230922846394 1.7123187258766581 1.4862926540609394 1.688346263714385
1.6198535146793185 1.6951955386178916 1.7739622000082178 1.7088940884249049 1.9109476980783504
1.7602636502012046 1.9760158096616636 1.9212216104336104 2.0547824710519897 1.7499897378459446
2.157521594604589 1.5513607656442523 2.2671099930606955 1.3938274428635997 2.3013563675782285
1.212321657920674 2.284233180319462 1.0273912355259949 2.2362882559949155 0.8424608131313158
2.1506723197010826 0.736297052126963 2.092453483021276 0.595886916605077 2.02396073398621 0.465750693438451
1.9897143594686768 0.34931302007883824 1.9691665347581568 0.2739709961402653 1.938344797692377
0.2499985339779921 1.828756399236271 0.24657389652623876 1.732866550587178 0.2534231714297454
1.6609491641003584
31 1.121342750340161 1.5443851928790728 0.309736219916278 1.4241132720063652 0.3703367846825063
1.3702461033252733 0.5184714985555088 1.3500459150698638 0.6969064948116255 1.296178746388772
0.8955416793231517 1.1648775227286108 1.1042769579623823 1.0672432794941318 1.2928120483462038
1.0436763931961541 1.49144723285773 1.0504097892812907 1.6530487389010053 1.1177437501326555
1.7641497743057573 1.1918111070691566 1.8920842999233505 1.2759785581333627 1.7405828880077796
1.3062788405164767 1.6900824173692561 1.3837128954955462 1.6968158134543925 1.5150141191557076
1.7001825114969606 1.646315342815869 1.733849491922643 1.7708831703908938 1.8685174136253726
1.8550506214550997 1.7001825114969606 1.9526848646895787 1.5453144015388218 2.043585711838921
1.3534126131124322 2.073885994222035 1.097543561877246 2.023385523583512 0.9224752636636975 1.9493181666470105
0.8181076243440821 1.8685174136253726 0.7002731928541938 1.7944500566888715 0.5757053652791689
1.7405828880077796 0.43093734944873463 1.717016001709802 0.32993640817168746 1.6833490212841196
0.309736219916278 1.6025482682624819 0.3164696160014145 1.508280723070571 0.309736219916278 1.4241132720063652
33 1.1213289849020498 1.6676615077055346 0.34144476126386447 1.5504379465553029 0.39370671451853756
1.5086283839515644 0.5156512721127748 1.4772712119987605 0.6201751786221211 1.4389457796120002
0.7874134290370751 1.3622949148384795 1.0138818931406588 1.2159614457253949 1.1915725342065473
1.1636994924707218 1.3762314357063925 1.1427947111688526 1.5852792487250849 1.1532471018197872
1.756001629357017 1.2264138363763295 1.9232398797719712 1.3065488313668283 1.7455492387060825
1.3448742637535887 1.6793507645834966 1.421525128527109 1.6305729415458017 1.567858597640194
1.6479935926306928 1.7246444574042132 1.703739676102344 1.8674937963003198 1.7978111919607558
1.919755749554993 1.9232398797719712 1.9476287912908186 1.7803905408758647 2.0626050884510994
1.5817951185081067 2.1601607345264893 1.4006203472252399 2.1636448647434676 1.1776360133386345
2.1253194323567075 0.9894929816218113 2.0800257395359907 0.8536119031596612 2.0103431351964263
0.752572126867293 1.9406605308568623 0.6585006110088815 1.902335098470102 0.5365560534146442 1.870977926517298
0.4215797562543632 1.8640096660833416 0.34841302169782085 1.8256842336965813 0.31008758931106056
1.7350968480551479 0.317055849745017 1.616636420677889 0.34144476126386447 1.5504379465553029
33 1.1551704214731677 1.5955318093096014 0.5033016754404919 1.5443777438173996 0.57224711043234

1.4926686675735135 0.67221799117052 1.4581959500775894 0.7618470566599226 1.4237232325816653
0.8376870351509557 1.385803243336149 0.9479997311379127 1.2720432755995992 1.110021503368756
1.1927560253589737 1.2720432755995992 1.151388764363865 1.437512319580035 1.1238105903671256 1.589192276562101
1.151388764363865 1.7167413312970201 1.2306760146044904 1.7891340380384608 1.3065159930955235
1.6719267985523187 1.3168578083443006 1.5443777438173996 1.3651196128385943 1.4754323088255514
1.489221395823921 1.471985037075959 1.63055953755721 1.5374832003182148 1.7546613205425368 1.6374540810563947
1.8373958425327546 1.8029231250368305 1.858079473030309 1.7581085922921291 1.9546030820188964
1.589192276562101 2.058021234506669 1.4168286890824804 2.0890466802530003 1.1893087536093814 2.033890332259522
1.010050622630576 1.9787339842660434 0.8859488396452494 1.9028940057750103 0.7997670459054391
1.8477376577815317 0.7032434369168516 1.8098176685360152 0.5998252844290793 1.7787922227896835
0.5205380341884539 1.7546613205425368 0.4860653166925298 1.6857158855506884 0.49985440369089945
1.5822977330629162 0.5033016754404919 1.5443777438173996
37 1.0112390553325967 1.680655712071299 0.24738616574117084 1.6011382393803557 0.29892495027058147
1.5702149686627094 0.3951306813921479 1.5530353738195726 0.4947723314823417 1.511804346196044
0.6493886850705735 1.4293422909489872 0.8040050386588052 1.3090851270470292 0.9448777163725276
1.2300589907685995 1.113237745835269 1.188827963145071 1.271290018392128 1.1682124493333068 1.3778035064195766
1.171648368301934 1.5461635358823178 1.2472385856117363 1.6732925377215306 1.3090851270470292
1.7248313222509413 1.3434443167333028 1.649241104941139 1.3709316684823218 1.5324198600078083
1.3881112633254586 1.501496589290162 1.4740092375411429 1.4705733185725156 1.62175375319212 1.49118883238428
1.75231867399996 1.5530353738195726 1.8725758379019182 1.6286255911293748 1.9241146224313288
1.7488827550313328 1.9756534069607394 1.632061510098002 2.089038732925443 1.4671373996038883
2.1989881399215188 1.298777370141147 2.2230395727019103 1.0823144751176226 2.1818085450783817
0.9036466887489991 2.1440134364234806 0.7662099300039041 2.0684232191136784 0.6596964419764556
2.0065766776783858 0.5634907108548891 1.9653456500548574 0.4672849797333227 1.9516019741803479
0.37451516758038367 1.934422379337211 0.28174535542744455 1.8863195137764277 0.26112984161568037
1.8072933774979982 0.26112984161568037 1.7110876463764317 0.257693922647053 1.6423692670038843
0.24738616574117084 1.6011382393803557
32 1.2049810267911485 1.7754810636373917 0.38110956269491075 1.6433164629964043 0.447541504816042
1.6048558649262756 0.5734125530455538 1.587373774894399 0.7167656913069422 1.5419203408115199
0.9335436077022127 1.4090564565692574 1.1153573440337297 1.321646006409874 1.321646006409874
1.2726961543206194 1.513948996760517 1.2482212282759921 1.6677913890410316 1.3041639163779974
1.821633781321546 1.3985672025501312 1.9335191575255566 1.4754883986903886 1.769187511225916
1.4859776527095145 1.7307269131557874 1.5489131768242703 1.7167412411302863 1.660798553028281
1.7132448231239108 1.7936624372705434 1.7272304951494122 1.9440084115446825 1.7656910932195407
2.0174331896785644 1.9125406494873045 2.0523973697423177 1.7761803472386668 2.143304237908076
1.650309299009155 2.2307146880674593 1.4894740707158898 2.2971466301885908 1.3461209324545014
2.2761681221503385 1.1188537620401051 2.223721852054709 0.9440328617213386 2.1537934919272024
0.7936868874471995 2.066383041767819 0.6573265851985617 2.0104403536658135 0.5384483729818006
1.9824690096148112 0.39159881671403673 1.9614905015765591 0.3321597106056562 1.8775764694235513
0.3496418006375328 1.7307269131557874 0.38110956269491075 1.6433164629964043
33 1.1510997118077568 1.492122534597603 0.35958060667129127 1.393802923002053 0.4143738419735833
1.3561325737317271 0.5239603125781673 1.325311378874188 0.6540942464211108 1.3047639156358284
0.7465578309937286 1.260244411952716 0.8972392280750316 1.1403842097289525 1.1164121692841997
1.0376468935371548 1.3184622244614015 0.9897028126476494 1.4931156619874573 0.9725799266156832
1.6472216362751535 1.0170994302987955 1.7739309929117038 1.0890155516330535 1.900640349548254
1.1712054045864917 1.777355570118097 1.174629981792885 1.695165717164659 1.2054511766504241 1.6609199451007264
1.3698308825573002 1.65407079068794 1.4862665075746706 1.695165717164659 1.6232495958304007 1.739685220847771
1.7294114892285914 1.886942040722681 1.7979030333564565 1.7636572612925239 1.9109140811674337
1.558182628908929 2.034198860597591 1.38010461417648 2.034198860597591 1.1780545589992781 1.9931039341208718
0.9897028126476494 1.9177632355802203 0.8698426104238856 1.8526962686587485 0.7499824082001219
1.79105387894367 0.630122205976358 1.7705064157053105 0.4931391177206281 1.7533835296733442 0.3801280699096508
1.7054394487838387 0.36300518387768455 1.5924284009728615 0.3732789154968643 1.4554453127171314
0.35958060667129127 1.393802923002053

31 1.1381435001812776 1.876251326999135 0.33565612861203153 1.779676765245042 0.40558448873953806
1.7097484051175356 0.5629232990264278 1.692266315085659 0.7307513633324436 1.629330790970903
0.9195579356767113 1.4999633247350157 1.1433286880847324 1.4300349646075092 1.3286388424226248
1.405560038562882 1.4859776527095145 1.405560038562882 1.6538057170155303 1.4859776527095145 1.853101543378924
1.5803809388816483 1.7272304951494122 1.5943666109071497 1.6852734790729083 1.6433164629964043
1.6852734790729083 1.7517054211940395 1.6957627330920342 1.8775764694235513 1.7097484051175356
2.0104403536658135 1.7517054211940395 2.0733758777805695 1.8391158713534226 2.1258221478761996
1.7202376591366615 2.213232598035583 1.5698916848625224 2.3286143922459686 1.3880779485310053
2.3600821543033463 1.1747964501421102 2.314628720220467 0.9824934597914672 2.258686032118462
0.8496295755492047 2.1817648359782047 0.7272549453260683 2.122325729869824 0.5804053890583045
2.087361549806071 0.4440450868096667 2.0698794597741945 0.35313821864390815 2.024426025691315
0.35313821864390815 1.91603706749368 0.3496418006375328 1.8391158713534226 0.33565612861203153
1.779676765245042
37 1.1949733647487641 1.713202194242449 0.4096999951778452 1.621439811424184 0.4826127061840719
1.5763033712774723 0.5763576203349348 1.5554711681328361 0.6978788053453127 1.5346389649882 0.8332881257854479
1.440894050837337 0.9548093107958258 1.347149136686474 1.1110508343805972 1.288124561110005 1.2638203241079293
1.232572019390975 1.4061737122629434 1.215211850103778 1.5311669311307605 1.2186838839612175 1.687408454715532
1.2777084595376869 1.7880974365812736 1.336733035114156 1.867954215302379 1.4096457461203826
1.7672652334366374 1.4235338815501402 1.6631042177134565 1.4721423555542912 1.6144957437093053
1.545055066560518 1.5901915067072296 1.6735203192857746 1.5971355744221085 1.7776813350089555
1.6457440484262595 1.8922584523044546 1.7394889625771224 1.97211523102556 1.8228177751556673 2.003363535742514
1.944338960166045 2.0346118404594686 1.829761842870546 2.097108449893377 1.6735203192857746 2.215157601046316
1.4756143894117306 2.2637660750504667 1.2360440532484143 2.215157601046316 1.0485542249466886
2.1596050593272857 0.8923127013619171 2.076276246748741 0.7812076179238574 2.0068355695999536
0.6596864329134796 1.944338960166045 0.5728855864774954 1.933922858593727 0.4409482998947995
1.9235067570214088 0.4096999951778452 1.8575381137300608 0.39928389360552713 1.7464330302920013
0.4096999951778452 1.6735203192857746 0.4096999951778452 1.621439811424184
36 0.9841642062039455 1.6650212130100155 0.22916528522500418 1.5694349836621497 0.26735949942917153
1.5173519642928308 0.37499773945909776 1.496518756545103 0.5069413885280395 1.4687411462147995
0.6458294401795572 1.3923527178064647 0.8055506995788025 1.267353471320099 1.0312437835125188
1.1840206403291882 1.1597152312901726 1.1631874325814606 1.3645751074761612 1.1492986274163088
1.493046555253815 1.1874928416204762 1.5867959901185895 1.2430480622810833 1.6909620288572278
1.312492088106842 1.7708226585568505 1.3576307048935854 1.6458234120704847 1.3749917113500252
1.5208241655841186 1.43401913330192 1.4861021526712392 1.5486017759144222 1.4826299513799512
1.6909620288572278 1.5069353604189668 1.8229056779261696 1.559018379788286 1.9062385089170801
1.743045048226547 1.9930435411992788 1.6249902043227569 2.1215149889769327 1.4027693216803285
2.225681027715571 1.1874928416204762 2.215264423841707 1.0104105757647912 2.17707020963754 0.8437449137829699
2.1145705863943567 0.718745667296604 2.045126560568598 0.6319406350144054 1.9652659308689753
0.5381912001496311 1.9166551127909441 0.4270807588284169 1.8888775024606406 0.3229147200897786
1.8784608985867768 0.23958188909886802 1.864572093421625 0.21180427876856447 1.8055446714697299
0.22222088264242829 1.7152674378962434 0.20833207747727653 1.5972125939924533 0.22916528522500418
1.5694349836621497
34 1.214859739859431 1.6295012646829097 0.4475278275242026 1.527887973656848 0.5419282286425892
1.4789396175213883 0.6503138743711069 1.4614580617587243 0.8006552539300188 1.405517083318199
0.9335150777262664 1.2936351264371482 1.1852494807086305 1.1782568584035646 1.3775465940979361
1.1363011245731707 1.5453695294195122 1.1258121911155723 1.6992072201309567 1.1572789914883677
1.8670301554525328 1.2307015256915572 2.010378912706379 1.2831461929795498 1.8915043335202626
1.2936351264371482 1.8145854881645402 1.3216056156574107 1.7691334431816135 1.4509691283011257
1.7621408208765479 1.5978141967075046 1.797103932401876 1.734170331656285 1.8390596662322702 1.821578110469606
1.9963936680962475 1.8775190889101312 1.842555977384803 2.013875223858912 1.6467625528429644
2.1292534918924955 1.4964211732840524 2.1432387365026266 1.3251019268099438 2.111771936129831
1.1188195688105065 2.045342024231707 0.9614855669465291 1.9719194900285177 0.8391146766078799
1.9089858892829268 0.7552032089470919 1.8705264666050656 0.6573064966761726 1.8495485996898686

0.5559134732527204 1.8460522885373358 0.4685056944393996 1.8355633550797372 0.4230536494564728
1.776126065486679 0.41606102715140714 1.6992072201309567 0.44053520521913697 1.5663473963347092
0.4475278275242026 1.527887973656848
30 1.3535605536332178 1.8283494809688583 0.7439446366782007 1.6228373702422145 0.8615916955017301
1.5986159169550174 1.0346020761245676 1.4705882352941178 1.2491349480968859 1.3598615916955017
1.4567474048442905 1.273356401384083 1.6193771626297577 1.259515570934256 1.782006920415225 1.301038062283737
1.9169550173010381 1.3806228373702423 2.0484429065743943 1.4429065743944636 1.9134948096885813
1.505190311418685 1.8131487889273357 1.6228373702422145 1.7889273356401385 1.806228373702422
1.8269896193771626 1.9930795847750864 1.9273356401384083 2.1176470588235294 2.0726643598615917
2.1660899653979238 1.972318339100346 2.262975778546713 1.7577854671280277 2.370242214532872 1.508650519031142
2.359861591695502 1.2698961937716262 2.273356401384083 1.1245674740484428 2.204152249134948 1.0173010380622838
2.131487889273356 0.9446366782006921 2.093425605536332 0.8373702422145328 2.055363321799308 0.7474048442906575
2.0588235294117645 0.657439446366782 2.0311418685121105 0.6470588235294118 1.9515570934256055
0.6678200692041523 1.8166089965397925 0.6920415224913494 1.6643598615916955 0.7439446366782007
1.6228373702422145
31 1.2628782768157543 1.4542287569783139 0.620679574114976 1.3084596427288682 0.6810700191640007
1.2715543707544643 0.8152710081618333 1.2614892965796267 0.9360518982598827 1.2346490987800602
1.0836729861574985 1.1440634312065232 1.2245840246052229 1.0534777636329862 1.3990453103024052
0.9796672196841782 1.5701515712746419 0.9461169724347201 1.7043525602724745 0.9595370713345034
1.858683697619982 1.0232825411084738 1.9895296618928688 1.1407084064815773 1.8922339448694403
1.201098851530602 1.751322906421716 1.2983945685540308 1.7110626097223662 1.4325955575518634
1.7244827086221495 1.6137668926989375 1.7848731536711742 1.731192758072041 1.952624389918465
1.8351985245453613 1.7848731536711742 1.9492693651935191 1.5902817196243169 1.9995947360677064
1.3587850136030555 1.9626894640933024 1.1641935795561982 1.862038722344928 1.0668978625327694
1.7748080794963366 0.9427619477097743 1.7077075849974204 0.8521762801362373 1.680867387197854
0.7313953900381879 1.6640922635731248 0.6408097224646508 1.6506721646733415 0.600549425765301 1.6037018185241
0.5904843515904636 1.492986002600888 0.600549425765301 1.3889802361275678 0.620679574114976 1.3084596427288682
31 1.2997239872602369 1.8216606334514185 0.6962295141142152 1.7371608955104683 0.7508357505153301
1.6927933284345624 0.8566353335424902 1.665490210234005 0.9692606961197897 1.6279484227082384
1.0887118382472287 1.4982586112555905 1.2184016496998766 1.3890461384533606 1.3719816895780121
1.3276141225021063 1.5733421863071235 1.2968981145264793 1.7371608955104683 1.3378527918273153
1.890740935388604 1.4231750362040574 1.802005801236792 1.4538910441796846 1.6347742022583778 1.546039068106566
1.61088397383289 1.685967548884423 1.6142968636079598 1.8088315807869315 1.7200964466351198 1.9692374002152067
1.815657360337071 2.0238436366163217 1.9248698331393008 2.095514321892785 1.8327218092124193
2.2081396844700842 1.665490210234005 2.2832232595216175 1.5016715010306603 2.2832232595216175
1.3276141225021063 2.2456814719958507 1.2081629803746674 2.1979010151448755 1.0989505075724377
2.122817440093342 1.0033895938704867 2.0784498730174366 0.8975900108433265 2.061385424142088
0.7713130891657481 2.0647983139171577 0.7269455220898423 2.010192077516043 0.6928166243391455
1.9043924944888826 0.6894037345640758 1.7883542421365135 0.6962295141142152 1.7371608955104683
28 0.979126053562758 1.6691966494064923 0.3978763359191266 1.5132903374701823 0.48969395190046344
1.492886422807663 0.6359220069818519 1.4384759837076115 0.7855507145069934 1.3330557579512616
0.9453813793633947 1.2174335748636522 1.152821178432341 1.1596224833198476 1.3262544530637552
1.1766257455386135 1.4656812032576372 1.231036184638665 1.632313173001545 1.305850538401236 1.523492294801442
1.3500590201700278 1.4248733739325985 1.475883160588897 1.4078701117138326 1.6595183925515706
1.4894857703639097 1.8227497098517251 1.649316435220311 1.962176460045607 1.5030883801389225
2.0777986431332165 1.367062282388794 2.132209082233268 1.1630231357636007 2.1526129968957872
0.9759872513571737 2.105003862683242 0.8569644158258111 2.0471927711394375 0.7447428851819549
1.9791797222643732 0.6427233118693583 1.914567325833062 0.5100978665629827 1.8941634111705428
0.40807829325038625 1.8703588440642702 0.32306198215655574 1.8125477525204654 0.3128600248252961
1.7037268743203624 0.32306198215655574 1.5711014290139869 0.3978763359191266 1.5132903374701823
29 1.1533062537431278 1.6648917297947936 0.4183649268608171 1.4999913231351247 0.5238064937932182
1.479583277922402 0.7550976728707431 1.4217604831530208 0.9625794658667581 1.2755028257951742
1.1530545545188375 1.170061258862773 1.350332324908491 1.1122384640933918 1.5340047318229961

1.1360478501749018	1.7040717752623527	1.2244827127633673	1.8197173648011151	1.2823055075327485	
1.710874456999927	1.3299242796957682	1.5884261857235902	1.3775430518587881	1.5169980274790604	
1.5442087544293575	1.506794004872699	1.7176771387375012	1.5850248448548032	1.9115535682583678	
1.7074731161311398	1.9795803856341103	1.8537307734889865	2.0408045212722787	1.6530516622305458	
2.125838042991957	1.4659779144472536	2.1870621786301254	1.2346867353697286	2.1836608377613382	
1.0340076241112879	2.0918246343040856	0.9251647163100997	2.0135937943219817	0.7959137632961887	
1.9353629543398776	0.6836695146262134	1.8911455230456449	0.5033984485804954	1.8639347960953478	
0.38775285904173296	1.8469280917514121	0.3707461546977973	1.7448878656877982	0.3945555407793072	
1.5646167996420803	0.4183649268608171	1.4999913231351247			
30	1.4717717224640001	1.7599375624151214	0.7824370488739053	1.5496070333795393	0.8931232655438724
1.5190729046429967	1.0534274414107214	1.4961723080905898	1.2480825121061807	1.396936389696826	
1.419836986249233	1.2518992781982485	1.648842951773303	1.1870142546330955	1.874032151205305	1.2022813190013668
2.0496033914404252	1.286250173026859	2.2099075673072743	1.3740357931444191	2.061053689716629	
1.4045699218809617	1.9121998121259833	1.4580046471699115	1.8358644902846266	1.67174354832571	
1.8511315546528981	1.8663986190211694	1.9236501104021868	2.0152524966118146	2.0686872219007646	
2.0763207540849002	2.1870069707548674	2.1373890115579854	1.9999854322435435	2.2442584621358845	
1.7900632971798127	2.3167770178851734	1.6297591213129639	2.301509953516902	1.4503711149857759	
2.2442584621358845	1.2709831086585877	2.1144884150055785	1.1831974885410277	2.061053689716629	
1.0457939092265858	1.98853513396734	0.8740394350835333	1.9732680695990685	0.7709867505977018	1.942733940862526
0.7251855574928879	1.874032151205305	0.7137352592166843	1.7289950397067275	0.7366358557690914	
1.5954082264843534	0.7824370488739053	1.5496070333795393			
30	1.350369100348251	1.7562108858142051	0.6287833679040944	1.6287761939684373	0.7196918066372165
1.5757462713741162	0.8863572776479404	1.5378677552353153	1.0605984518864244	1.431807910046673	
1.1969611099861075	1.2992331035608697	1.3674144326107114	1.2234760712832682	1.5984733810573968	
1.178021851916707	1.784078110137521	1.2083246648277477	1.8863501037122834	1.2613545874220689	2.003773503742566
1.3257480648580304	1.8560472908012426	1.393929393907872	1.7613510004542405	1.5113527939381548	1.73862389077096
1.6818061165627587	1.7499874456126001	1.8484715875734825	1.8105930714346816	1.9961978005148058	
1.9507435811482448	2.064379129564647	2.106045497317328	2.098469794089568	1.9355921746927245	2.212105342505971
1.7348360391570798	2.3333165941501335	1.4848378326409941	2.318165187694613	1.2348396261249084	
2.231044600575371	1.1022648196391054	2.1439240134561293	0.9659021615394222	2.0681669811785275	
0.8068123937564585	2.0227127618119662	0.6893889937261758	2.0227127618119662	0.609844109834694	
1.9886220972870456	0.5568141872403728	1.871198697256763	0.5833291485375334	1.696957523018279	
0.6287833679040944	1.6287761939684373				
31	1.2788201836390511	1.7706703269458628	0.5735685707254292	1.5961809566898457	0.6867728938949218
1.5584461823000149	0.8112976493813637	1.513164453032218	0.9773306566966196	1.3773192652288269	
1.1622310512067908	1.252794509742385	1.3961866524237423	1.1848719158406893	1.5697666146169642	
1.1924188707186556	1.7093852798593385	1.249021032303402	1.8263630804678141	1.3093966713271312	
1.9282469713203574	1.3584518780339114	1.7886283060779833	1.4112805621796745	1.6641035505915414	
1.5320318402271333	1.6339157310796766	1.6603300731525583	1.649009640835609	1.7961752609559494	
1.7207057121762876	1.9584347908322222	1.8150426481508648	2.04145129448985	1.9433408810762898	
2.1056004109525626	1.8112691707118818	2.1886169146101904	1.6603300731525583	2.2980477603407	1.4641092463254377
2.328235579852565	1.2943027615711988	2.2980477603407	1.12449627681696	2.2527660310729027	0.9848776115745858
2.169749527415275	0.8829937207220424	2.109373888391546	0.7735628749915329	2.075412591440698	0.6528115969440741
2.033904339611884	0.5320603188966153	1.9773021780271376	0.5131929317016999	1.8490039451017126	
0.5358337963355984	1.6716505054695074	0.5735685707254292	1.5961809566898457		
29	1.0529115646258502	1.360921768707483	0.47619047619047616	1.1870748299319729	0.6020408163265306
1.1734693877551021	0.7346938775510204	1.153061224489796	0.8537414965986394	1.0850340136054422	
0.9795918367346939	0.9829931972789115	1.1496598639455782	0.9285714285714286	1.3061224489795917	
0.9183673469387755	1.4421768707482994	0.9455782312925171	1.5442176870748299	1.0034013605442176	
1.6394557823129252	1.0816326530612246	1.534013605442177	1.1360544217687074	1.401360544217687	
1.2074829931972788	1.336734693877551	1.3673469387755102	1.3911564625850341	1.5374149659863945	
1.4965986394557824	1.6360544217687074	1.598639455782313	1.6972789115646258	1.4863945578231292	
1.7857142857142858	1.3095238095238095	1.846938775510204	1.1156462585034013	1.816326530612245	

0.9319727891156463 1.7414965986394557 0.8401360544217688 1.6428571428571428 0.7653061224489796
1.5952380952380953 0.6836734693877551 1.5578231292517006 0.5510204081632653 1.5442176870748299
0.43537414965986393 1.5 0.3945578231292517 1.41156462585034 0.42857142857142855 1.2619047619047619
0.47619047619047616 1.1870748299319729
30 1.2004375956202644 1.6423279154472177 0.45889334565669554 1.530786011257783 0.5924518567060323
1.4862665075746706 0.7328595221681556 1.4520207355107382 0.8869654964558519 1.366406305350907
1.054769779569121 1.2088757538568173 1.2362723715079633 1.1164121692841997 1.4348978494787719
1.0684680883946942 1.599277555385648 1.082166397220267 1.7362606436413779 1.147233364141739 1.9314615444057932
1.2054511766504241 1.7876293017372766 1.2842164523974688 1.667769099513513 1.3869537685892663
1.6472216362751535 1.5342105884641761 1.6711936767199063 1.7362606436413779 1.7807801473244902
1.8561208458651417 1.955433584850546 1.9485844304377595 1.79105387894367 2.0787183642807032 1.6164004414176143
2.1814556804725007 1.4143503862404123 2.1951539892980736 1.2533952575399296 2.1540590628213545
1.0376468935371548 2.0615954782487367 0.9075129596942113 1.9896793569144784 0.8013510662960206
1.9314615444057932 0.6986137501042231 1.9143386583738269 0.5239603125781673 1.8972157723418608
0.4794408088950551 1.8424225370395688 0.45889334565669554 1.7362606436413779 0.4554687684503023
1.5924284009728615 0.45889334565669554 1.530786011257783
30 1.2143798735705695 1.6636222130768177 0.5589098866657459 1.5353187248167477 0.6733854056213806
1.4915486734513579 0.8282640489142982 1.457879403170289 0.936005713813719 1.4006416436924716
1.0605820138536743 1.319835395017906 1.2053598760622712 1.2356622193152333 1.3703393004395095
1.1952590949779505 1.5050163815637856 1.212093730118485 1.6363265356599548 1.255863781483875
1.7508020546155896 1.3097346139335853 1.8787452816836518 1.3939077896362577 1.6868304410815584
1.4174762788330062 1.6161249734913135 1.5386856518448546 1.592556484294565 1.6901973681096654
1.6161249734913135 1.8316083032901551 1.693564295137772 1.94608382224579 1.8147736681496207 1.9831200195549659
1.6497942437723825 2.0706601222857453 1.4982825275075717 2.1245309547354556 1.3400369571865474
2.1379986628478833 1.1716906057812022 2.1043293925668145 1.0134450354601778 2.016789289836035
0.898969516504543 1.939349968189576 0.7743932164645877 1.8888460627679726 0.659917697508953 1.872011427627438
0.5454421785533182 1.8450760114025828 0.5084059812441424 1.7541689816436965 0.518506762328463
1.6127580464632065 0.5589098866657459 1.5353187248167477
32 1.020854638971688 1.6468170044180588 0.3537394503538616 1.5408074135605705 0.48979308510534686
1.506794004872699 0.6088400155128965 1.4693792553160407 0.7244856050516589 1.3877470744651494
0.8061177859025501 1.2823055075327485 0.9489741023916096 1.190469304075496 1.1190411458309661
1.139449191043689 1.2482920988448771 1.1224424866997533 1.397951097071511 1.139449191043689 1.5238007092166348
1.1972719858130703 1.6496503213617586 1.272101484926387 1.4897873005287634 1.3129175753518325
1.350332324908491 1.397951097071511 1.323121597958194 1.4965899822663378 1.3061148936142584 1.6224395944114616
1.3571350066460652 1.7482892065565854 1.4659779144472536 1.8843428413080707 1.656453003099333
1.969376363027749 1.5510114361669318 2.0646139073537886 1.4183591422842337 2.1632527925486156
1.2618974623200256 2.176858156023764 1.0782250554055206 2.149647429073467 0.9217633754413125 2.102028656910447
0.7925124224274016 2.0101924534531945 0.6904721963637876 1.948968317815026 0.5816292885625994
1.918356249995942 0.44217431294232706 1.9115535682583678 0.3843515181729458 1.8979482047832192
0.3401340868787131 1.7550918882941597 0.34353542774750023 1.6258409352802488 0.3537394503538616
1.5408074135605705
28 1.3916238151716331 1.5215622628405696 0.7702658745909607 1.3885055897231793 0.8817517248607051
1.361478716930514 1.0101293706258652 1.3276951259396823 1.1486420936882749 1.2432361484626033
1.2905331758497676 1.1216152208956096 1.4898563626956742 1.023642807022198 1.7026929859379134
1.0135077297249484 1.8986378136847366 1.0574263980130294 2.0202587412517303 1.1283719390937759
1.8918810954865704 1.216209275669938 1.8209355544058239 1.3682354351286803 1.8243139135049071
1.4932347217947572 1.885124377288404 1.6452608812534997 2.047285614044396 1.7668818088204934
1.9324214046755683 1.8817460181893209 1.8074221180094914 1.9662049956663998 1.6452608812534997
1.9864751502608988 1.4898563626956742 1.9560699183691503 1.297289894047934 1.885124377288404
1.2026958392736053 1.8344489908021566 1.0776965526075284 1.7702601679195766 0.9459405477432852
1.736476576928745 0.8243196201762913 1.7297198587305787 0.7635091563927945 1.6824228313434144
0.7466173608973786 1.550666826479171 0.7668875154918776 1.425667539813094 0.7702658745909607
1.3885055897231793

30 1.057234593697837 1.559582782132805 0.31520896490515093 1.4743645132660286 0.46095074437742506
1.413356326510193 0.6202498986843293 1.3726842020063026 0.7388769284873431 1.2710038907465764
0.9117334576288775 1.1015367053136995 1.0981473616050421 1.0066350814712886 1.2947292967071793
0.9625736132587406 1.4608071384313985 0.9964670503453159 1.6336636675729328 1.0574752371011515
1.7624587285019193 1.1218727675656448 1.5218153251872342 1.2167743914080558 1.4947005755179739
1.369294858297645 1.5184259814785765 1.5590981059824671 1.603159574195015 1.6946718543287687
1.7116185728720563 1.7692374159192343 1.81668822784044 1.8132988841317823 1.6709464483681657
1.9658193510213715 1.4845325443920012 2.0641103185724403 1.274393234455234 2.0912250682417004
1.0879793304790695 2.0471636000291524 0.9185121450461926 1.9827660695646592 0.7626023344479459
1.8912537894309056 0.6744793980228498 1.8505816649270153 0.5050122125899731 1.8438029775097002
0.35927043311769896 1.823466915257755 0.30504093377917835 1.7455120099586316 0.2948729026532057
1.6370530112815904 0.3016515900705208 1.5252046688958918 0.31520896490515093 1.4743645132660286
28 1.3180577510338796 1.6952586002727361 0.773630989267721 1.5742883886408647 0.9053847385316561
1.5371270747459087 1.0371384877955911 1.4425346393769296 1.2026752496913042 1.2871327812707498
1.4796959532718856 1.2026752496913042 1.6350978113780654 1.229701659796727 1.7837430669578895
1.2702412749548608 1.908740213695469 1.3107808901129947 1.7668515606420006 1.3378073002184172
1.6722591252730215 1.432399735587396 1.6486110164307768 1.604693100009465 1.6891506315889107
1.7769864644315339 1.756716656852467 1.871578899800513 1.908740213695469 1.9323883225377136 1.797256272010601
2.0540071680121152 1.6182063050621764 2.1317080970652054 1.4019950242187957 2.1317080970652054
1.2567280699021495 2.07765527685436 1.1452441282172814 2.016845854117159 1.0675431991641915 1.9729279376958475
0.9695724625320347 1.9357666238008915 0.8682234246367 1.9154968162218247 0.7972790981099658 1.8918487073795798
0.7297130728464094 1.8209043808528456 0.7229564703200537 1.7060421379047996 0.7263347715832316
1.6114497025358208 0.773630989267721 1.5742883886408647
30 1.135664198920347 1.7499595090179145 0.49658714930343156 1.6632268219820414 0.5714153498834007
1.6122075943138805 0.6870589325978984 1.5986024669357044 0.8299127700687486 1.5509845211120876
0.9285499435605261 1.4523473476203101 1.0680024991868322 1.3401050467503564 1.248270436947667
1.2686781280149313 1.4217358110194136 1.2584742824812991 1.5952011850911603 1.306092228304916
1.7312524588729223 1.3605127378176207 1.8366921960537879 1.3945255562630612 1.649621694603865
1.4591499113093982 1.57819477586844 1.642819130914777 1.6020037487802483 1.7550614317847306 1.6734306675156734
1.911520396633757 1.826488350520156 1.9999537245919023 1.6326152853811449 2.1394062802182088
1.4727550386875745 2.2142344807981775 1.30949351014946 2.2142344807981775 1.115620445010449 2.1598139712854727
1.0033781441404952 2.0951896162391357 0.9217473798714381 2.037367824881887 0.8299127700687486
1.9999537245919023 0.717670469198795 1.9931511609028143 0.6054281683288412 1.9931511609028143
0.4931858674588875 1.97274346983555 0.4795807400807113 1.8639024508101403 0.4727781763916232
1.7414563044065545 0.49658714930343156 1.6632268219820414
32 1.0592792707744654 1.7570370871124006 0.43623915455426293 1.6006621286337186 0.5402654144864333
1.5603938989825559 0.68791558987403 1.5234813551356567 0.8456328226744174 1.399320980377905 0.9899273122577505
1.2852276630329438 1.1879127747093006 1.2181139469476727 1.3624084365310059 1.201335517926355
1.5234813551356567 1.244959433381781 1.704688388565889 1.32885157848837 1.5603938989825559 1.37583117974806
1.436233524224804 1.4731460680717032 1.4026766661821686 1.6308633008720907 1.4026766661821686
1.7248225033914704 1.4395892100290677 1.872472678779067 1.5167699835271296 1.979854624515501
1.5872393854166644 2.010055796753873 1.7483123040213153 2.046968340600772 1.5603938989825559 2.177840086967051
1.4093880377906955 2.244953803052322 1.261737862403099 2.2516651746608494 1.100664943798448 2.2113969450096866
0.9597261400193785 2.147638914728679 0.825498707848836 2.0704581412306173 0.7214724479166656 2.003344425145346
0.6241575595930223 1.9697875671027103 0.4999971848352706 1.9697875671027103 0.42617209714147225
1.949653452277129 0.3322128946220925 1.8691169929748035 0.32214583720930184 1.7449566182170517
0.36241406686046457 1.6241519292635636 0.43623915455426293 1.6006621286337186
32 0.9757520448634527 1.5373650230664337 0.3434323967428301 1.3871975240984904 0.40403811381509425
1.340059744153396 0.5252495479596225 1.340059744153396 0.6195251078498112 1.2996559327718866
0.7407365419943395 1.2020133885999054 0.9360216303383017 1.05723306448283 1.154875608654811 1.0168292531013206
1.346793712716981 1.0269302059466978 1.4410692726071694 1.080801954455377 1.5993175338514147
1.1515086243730186 1.4309683197617922 1.2255822785724526 1.3299587913080186 1.3265918070262261
1.2895549799265091 1.4680051468615092 1.3097568856172639 1.6296203923875467 1.3905645083802827

1.6935930937416035 1.4646381625797167 1.750831826532075 1.5387118167791507 1.7845016693499995
1.6296203923875467 1.8518413549858488 1.4949410211158487 1.9326489777488676 1.3265918070262261
2.020190569075471 1.1515086243730186 2.020190569075471 0.9595905203108489 1.9696858048485846
0.8047092433483961 1.8888781820855656 0.6733968563584904 1.801336590758962 0.5622863750593395
1.7541988108138675 0.47474478373273576 1.7407308736866978 0.3804692238425471 1.7373638894049053
0.32659747533386785 1.6565562666418865 0.3097625539249056 1.5218768953701884 0.31312953820669803
1.444436256888962 0.3434323967428301 1.3871975240984904
33 1.054989932885906 1.7249395973154364 0.2785234899328859 1.6140939597315436 0.3288590604026846
1.5671140939597314 0.4597315436241611 1.5369127516778522 0.6644295302013423 1.4496644295302012
0.8053691275167785 1.332214765100671 1.016778523489933 1.1979865771812082 1.1879194630872483
1.1375838926174497 1.3590604026845639 1.1375838926174497 1.5134228187919463 1.1946308724832215
1.651006711409396 1.2483221476510067 1.8120805369127517 1.3053691275167785 1.6476510067114094
1.3523489932885906 1.5604026845637584 1.4597315436241611 1.5335570469798658 1.6275167785234899
1.5503355704697988 1.7684563758389262 1.6174496644295302 1.9228187919463087 1.7046979865771812
1.9865771812080537 1.8221476510067114 2.030201342281879 1.6140939597315436 2.161073825503356
1.4731543624161074 2.238255033557047 1.25503355704698 2.2583892617449663 1.0771812080536913 2.221476510067114
0.9429530201342282 2.1577181208053693 0.8221476510067114 2.0805369127516777 0.7315436241610739
2.033557046979866 0.6174496644295302 2.0 0.48322147651006714 1.983221476510067 0.3825503355704698
1.9798657718120805 0.27181208053691275 1.9463087248322148 0.2550335570469799 1.8355704697986577
0.2651006711409396 1.7080536912751678 0.2785234899328859 1.6140939597315436
35 1.368456544368784 1.5131913060214937 0.6135557968742643 1.3559244129817996 0.6983010726856268
1.305077247494982 0.8440629470811702 1.2813485702678007 1.0338923648986222 1.2169421606511652
1.1627051841318932 1.1186376407099847 1.277958759235346 1.0203331207688042 1.45422893292298 0.9694859552819868
1.603380618350978 0.9491470890872598 1.8033794692657936 0.9898248214767137 1.9593107767587006
1.0711802862556217 2.0474458636025177 1.1355866958722571 1.955920965726246 1.1491459400020752
1.820328524428066 1.2033829165213472 1.799989658233339 1.305077247494982 1.759311925843885 1.4305002556957986
1.7762609810061576 1.5932111852536146 1.820328524428066 1.7321934375842492 1.8847349340447015
1.7830406030710666 2.0542254856674265 1.8372775795903384 1.935582099531519 1.9254126664341555
1.806769280298248 2.016937564310427 1.6610074059027045 2.071174540829699 1.5186353425396155 2.057615296699881
1.3627040350467086 2.010157942245518 1.2203319716836196 1.949141343661337 1.1186376407099847 1.874565500947338
1.0135534987038952 1.7965998472008846 0.9084693566978057 1.7491424927465216 0.7999954036592618
1.7355832486167035 0.6508437182312639 1.7220240044868855 0.6067761748093553 1.6542277838377957
0.5864373086146284 1.5220251535720701 0.5999965527444463 1.4033817674361626 0.6135557968742643
1.3559244129817996
